# Cyclopropene derivatives of aminosugars for metabolic glycoengineering

**DOI:** 10.3762/bjoc.15.54

**Published:** 2019-03-04

**Authors:** Jessica Hassenrück, Valentin Wittmann

**Affiliations:** 1 University of Konstanz, Department of Chemistry and Konstanz Research School Chemical Biology (KoRS-CB), Universitätsstr. 10, 78457 Konstanz, Germany

**Keywords:** bioorthogonal chemistry, carbohydrates, cyclopropenes, inverse electron-demand Diels–Alder reaction, metabolic engineering

## Abstract

Cyclopropenes have been proven valuable chemical reporter groups for metabolic glycoengineering (MGE). They readily react with tetrazines in an inverse electron-demand Diels–Alder (DAinv) reaction, a prime example of a bioorthogonal ligation reaction, allowing their visualization in biological systems. Here, we present a comparative study of six cyclopropene-modified hexosamine derivatives and their suitability for MGE. Three mannosamine derivatives in which the cyclopropene moiety is attached to the sugar by either an amide or a carbamate linkage and that differ by the presence or absence of a stabilizing methyl group at the double bond have been examined. We determined their DAinv reaction kinetics and their labeling intensities after metabolic incorporation. To determine the efficiencies by which the derivatives are metabolized to sialic acids, we synthesized and investigated the corresponding cyclopropane derivatives because cyclopropenes are not stable under the analysis conditions. From these experiments, it became obvious that *N*-(cycloprop-2-en-1-ylcarbonyl)-modified (Cp-modified) mannosamine has the highest metabolic acceptance. However, carbamate-linked *N*-(2-methylcycloprop-2-en-1-ylmethyloxycarbonyl)-modified (Cyoc-modified) mannosamine despite its lower metabolic acceptance results in the same cell-surface labeling intensity due to its superior reactivity in the DAinv reaction. Based on the high incorporation efficiency of the Cp derivative we synthesized and investigated two new Cp-modified glucosamine and galactosamine derivatives. Both compounds lead to comparable, distinct cell-surface staining after MGE. We further found that the amide-linked Cp-modified glucosamine derivative but not the Cyoc-modified glucosamine is metabolically converted to the corresponding sialic acid.

## Introduction

**Figure 1:**
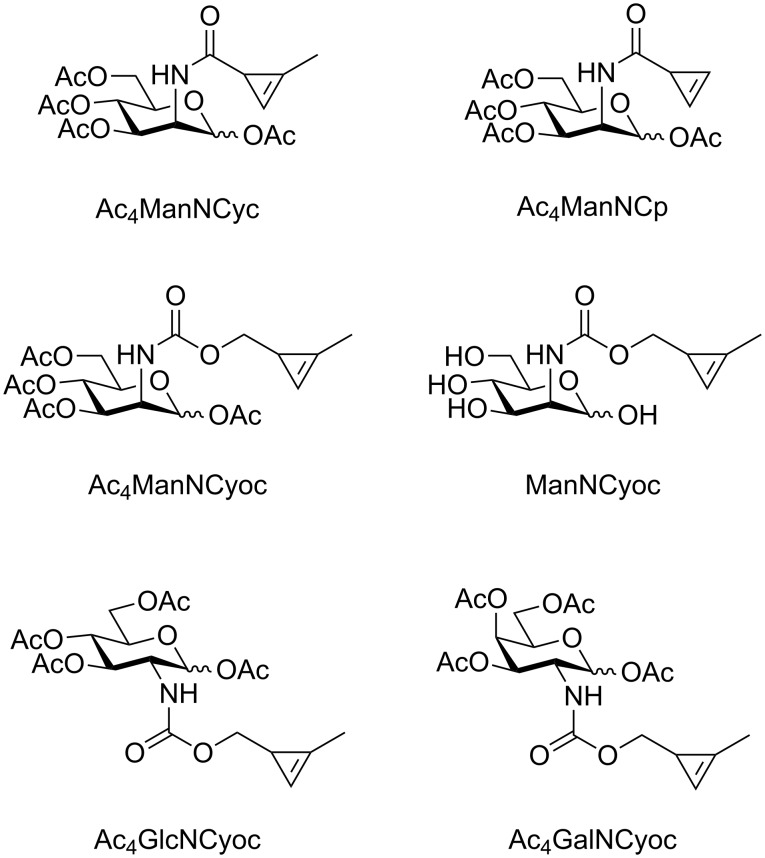
Cyclopropene-modified mannosamine, glucosamine and galactosamine derivatives employed for MGE.

Carbohydrates are an important class of biological molecules involved in many fundamental biological processes [1]. An important tool to visualize glycoconjugates in vitro and in vivo is metabolic glycoengineering (MGE) [2–4]. In this approach, cells are cultivated with an unnatural carbohydrate derivative carrying a chemical reporter group. After cellular uptake, the derivative is deacetylated, metabolized by the biosynthetic machinery and incorporated into glycoconjugates. The chemical reporter group can then be visualized using a bioorthogonal ligation reaction [5–6]. Mannosamine derivatives are of special interest because they are metabolized to sialic acids and then displayed as terminal structures on the cell surface [7]. Various carbohydrate derivatives with different reporter groups have been applied for MGE [2–4]. For example, azides and alkynes can be visualized by the Staudinger ligation [8] or the azide–alkyne cycloaddition, that can be performed either copper-catalyzed [9–10] or strain-promoted [11–12]. Another type of reporter group that has been proven to be a valuable tool are electron-rich or strained alkenes, that can be ligated through the inverse electron-demand Diels–Alder (DAinv) reaction with 1,2,4,5-tetrazines [13–17]. This reaction is advantageous since it is fast, irreversible, and does not require a toxic heavy metal catalyst. Different terminal alkenes that are connected to sugars by an amide [18], carbamate [19], or most recently a urea linkage [20] have been reported. Terminal alkenes are small which is beneficial for being accepted by the enzymes involved in glycan biosynthesis. However, they react only slowly in the DAinv reaction [20]. In contrast, ring-strained alkenes, such as norbornenes, have high DAinv reaction kinetics, but suffer from low incorporation efficiencies [21]. Cyclopropenes, that combine fast reaction kinetics and small size, turned out to be excellent reporters for application in MGE [22–27]. Three cyclopropene-derivatized mannosamine derivatives have been reported: Ac_4_ManNCyc [23], Ac_4_ManNCyoc [24–25], and Ac_4_ManNCp [27] (Figure 1). They differ in their type of linkage (amide or carbamate) and the presence/absence of a stabilizing methyl group at the double bond. Kinetic studies using model compounds revealed that a carbamate-linked cyclopropene reacts two orders of magnitude faster than an amide-linked [28] and that removal of the stabilizing methyl group results in a 9-fold second-order rate constant [27]. However, these studies have been performed with different model compounds and under different reaction conditions and, therefore, are not comparable. Additionally, the influence of the sugar derivative on the reaction rate has not been taken into account. Ac_4_ManNCyoc as well as Ac_4_ManNCp were shown to give after MGE a better membrane staining than Ac_4_ManNCyc [25,27]. A direct comparison of Ac_4_ManNCyoc and Ac_4_ManNCp in one biological experiment, however, is still unexplored.

Here we present a comparative study with all three derivatives Ac_4_ManNCyc, Ac_4_ManNCyoc, and Ac_4_ManNCp under the same conditions allowing a direct comparison of Ac_4_ManNCyoc and Ac_4_ManNCp. Our study includes the determination of second-order rate constants of the deacetylated (water-soluble) sugars, the performance of the sugars in MGE, and the assessment of their metabolic acceptance. The studies uncovered that Ac_4_ManNCp is much better accepted than Ac_4_ManNCyoc although their membrane staining intensity after MGE is comparable. The high metabolic acceptance of the Cp-modified sugar inspired us to develop novel derivatives of glucosamine and galactosamine containing this cyclopropene modification and to explore their behavior in MGE both for membrane-bound and intracellular glycoproteins.

## Results and Discussion

### Kinetic studies

**Figure 2:**
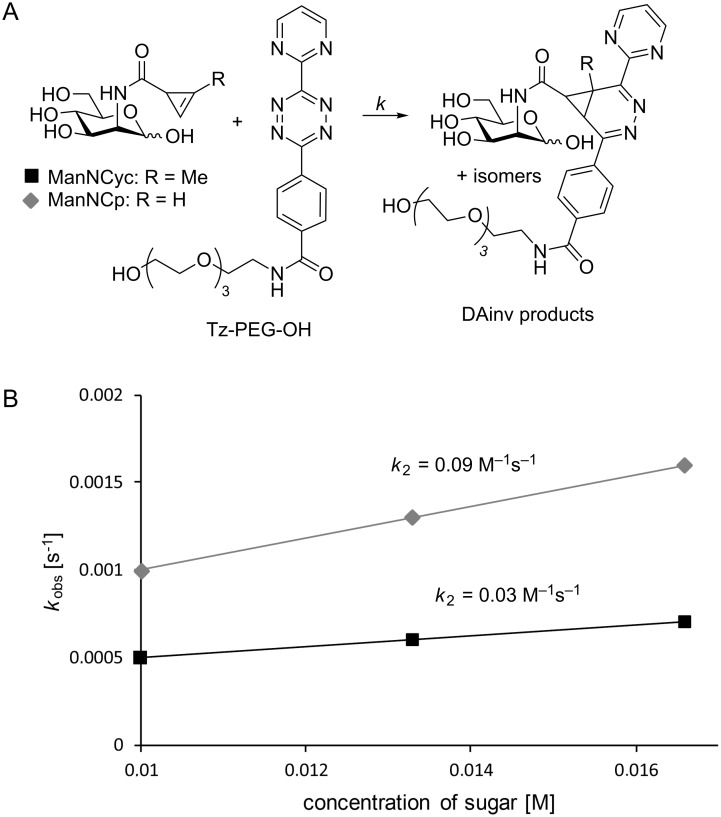
A) Reaction of ManNCyc and ManNCp, respectively, with Tz-PEG-OH to determine second-order rate constants *k*_2_. B) Plot of *k*_obs_ against the sugar concentrations. The slopes equal the second-order rate constants *k*_2_.

The second-order rate constant *k*_2_ of ManNCyoc has previously been reported to be *k*_2_ = 0.99 M^−1^s^−1^ [24]. To determine *k*_2_ of Cyc- and Cp-modified mannosamine, we synthesized ManNCyc and ManNCp according to published protocols [23,27], omitting the final peracetylation step. In this way, water-soluble compounds were obtained that allowed the determination of rate constants in aqueous solution. As reported for ManNCyoc, an excess of ManNCyc and ManNCp, respectively, was reacted with water-soluble tetrazine Tz-PEG-OH in acetate buffer (pH 4.7, Figure 2A). The decrease of absorption of Tz-PEG-OH at λ_max_ = 522 nm was measured and pseudo-first-order rate constants *k*_obs_ were determined. From these values second-order rate constants *k*_2_ were determined to be 0.03 M^−1^s^−1^ (ManNCyc) and 0.09 M^−1^s^−1^ (ManNCp) (Figure 2B). These numbers illustrate that the removal of the stabilizing methyl group results in a triplication of the rate constant. Comparison of the rate constant of ManNCyc with the published one of ManNCyoc (0.99 M^−1^s^−1^ [24]), which was determined under the same conditions, shows that the carbamate linkage instead of the amide linkage results in a 33-fold second-order rate constant. Obviously, the presence of the carbamate linkage has a higher impact on the reaction rate than the removal of the methyl group; *k*_2_ of ManNCyoc is eleven times higher than that of ManNCp. In conclusion, the three cyclopropene-modified sugars rank in the order ManNCyc, ManNCp, and ManNCyoc with the latter being the fastest.

### Metabolic glycoengineering with mannosamine derivatives

**Scheme 1:**
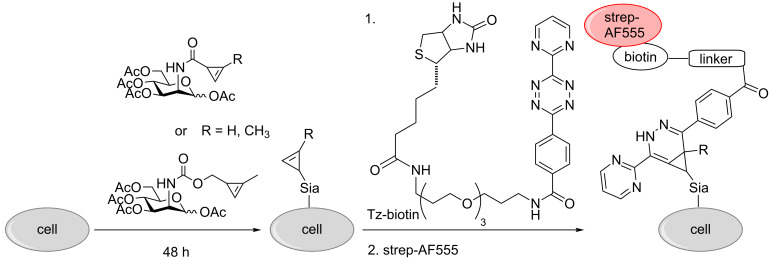
MGE with cyclopropene-modified mannosamines. Cells were grown with sugar for 48 hours and then incubated with Tz-biotin, followed by strep-AF555.

**Figure 3:**
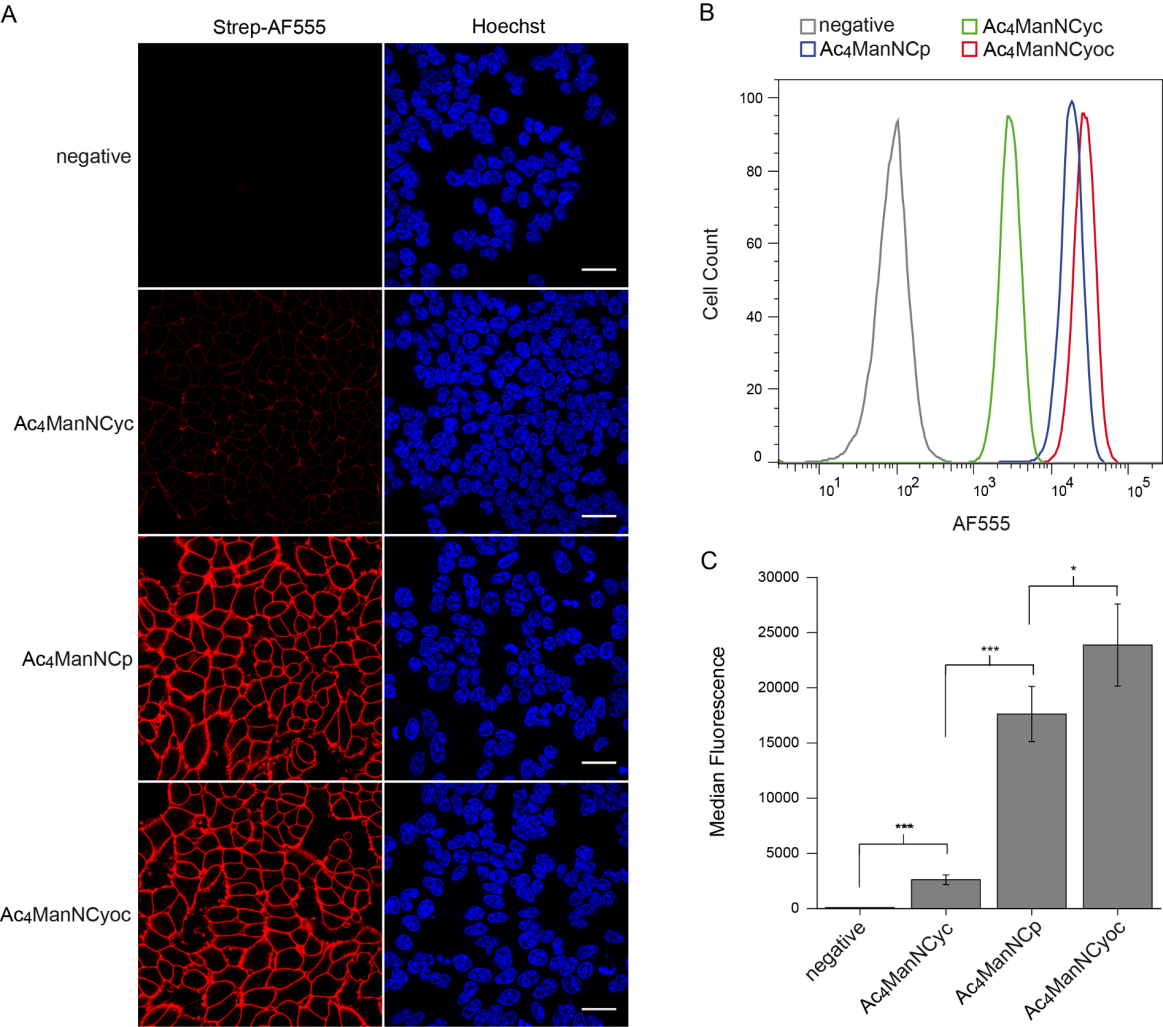
HEK 293T cells were grown with 100 μM Ac_4_ManNCyc, Ac_4_ManNCp, Ac_4_ManNCyoc or DMSO only (negative control) for 48 h. Cells were incubated with Tz-biotin (100 μM) for 1 h (A) or 30 min (B,C) at 37 °C followed by incubation with strep-AF555. A) Results from confocal fluorescence microscopy. Nuclei were stained with Hoechst 33342. Scale bar: 30 μm. B) Histogram from flow cytometry experiments. C) Median fluorescence from five independent flow cytometry experiments.

All three mannosamine derivatives Ac_4_ManNCyc, Ac_4_ManNCp, and Ac_4_ManNCyoc were employed in metabolic glycoengineering. To this end, HEK 293T cells were cultivated for 48 h in the presence of the respective sugar or DMSO only as negative control. Subsequently, the cells were incubated with Tz-biotin, followed by incubation with streptavidin-AlexaFluor 555 (strep-AF555) for visualization (Scheme 1). In confocal fluorescence microscopy experiments, all sugars showed a distinct cell membrane staining in comparison to the negative control (Figure 3A). As expected [25,27], the staining intensity obtained with Ac_4_ManNCyc was much lower than that of Ac_4_ManNCyoc and Ac_4_ManNCp and required different microscope settings to become clearly visible (Figure S1, Supporting Information File 1). Surprisingly, Ac_4_ManNCp and Ac_4_ManNCyoc resulted in a similar staining intensity although Ac_4_ManNCyoc reacts significantly faster in the DAinv reaction.

To verify and to quantify these findings, we also analyzed the labeled cells by flow cytometry. We used the same conditions for MGE as described above (Scheme 1), but after incubation with strep-AF555, cells were released with trypsin, resuspended in buffer, and then subjected to flow cytometry analysis. The obtained results coincided with those of the fluorescence microscopy experiments (Figure 3B,C). Ac_4_ManNCyc gave a significantly higher fluorescence intensity than the negative control, which, however, is exceeded by far from that of Ac_4_ManNCp and Ac_4_ManNCyoc. The experiments further revealed that Ac_4_ManNCyoc results in a slightly though significantly brighter staining than Ac_4_ManNCp. The similar fluorescence intensity of cells engineered with either Ac_4_ManNCp or Ac_4_ManNCyoc suggests that Ac_4_ManNCp with its much lower DAinv reactivity is much more efficiently metabolized and converted to the corresponding sialic acid than Ac_4_ManNCyoc.

### Determination of incorporation efficiencies

To confirm the hypothesis of different metabolization efficiencies of the mannosamine derivatives, we intended to quantify the proportion of cellular sialic acids that are labeled with a cyclopropene residue after MGE (i.e., the incorporation efficiency, *IE*). After the MGE experiments, we released the sialic acids from the cells by acetic acid treatment at elevated temperature and labeled them by addition of 1,2-diamino-4,5-methylenedioxybenzene (DMB) [29–31]. As described earlier [20], DMB selectively reacts with α-keto acids such as *N*-acetylneuraminic acid (Neu5Ac), the most abundant natural sialic acid in human cells [1], forming a fluorophore. Analysis by RP-HPLC equipped with a fluorescence detector allows the detection of natural and modified sialic acids. The incorporation efficiency *IE* can be calculated from the integrals *I* of the RP-HPLC signals of DMB-labeled Neu5Ac (*I*_Neu5Ac_) and the respective DMB-labeled modified sialic acid (*I*_Neu5R_) according to the formula *IE* = *I*_Neu5R_ (*I*_Neu5R_ + *I*_Neu5Ac_)^−1^ 100%. Unfortunately, it turned out that cyclopropene derivatives were not stable under these conditions, an observation that has also been made by Ye and co-workers [27]. Therefore, we decided to investigate the corresponding cyclopropane derivatives instead. We expected them to be stable under the DMB labeling conditions and during the preparation of reference compounds. Furthermore, their structure was expected to resemble that of the cyclopropenes as close as possible providing valuable information on the metabolic acceptance although it has to be kept in mind that (methyl)cyclopropanes are not plane in contrast to cyclopropenes.

**Scheme 2:**
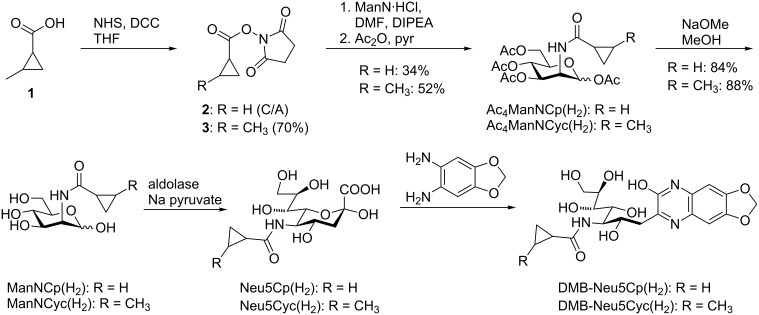
Synthesis of Ac_4_ManNCp(H_2_) and Ac_4_ManNCyc(H_2_) and the corresponding DMB-labeled sialic acids. C/A = commercially available.

Scheme 2 shows the synthesis of the mannosamine derivatives Ac_4_ManNCp(H_2_) and Ac_4_ManNCyc(H_2_) (H_2_ indicates the cyclopropane moiety, i.e., the formal hydrogenation of the corresponding cyclopropene) as well as their transformation into the DMB-labeled sialic acids that served as reference compounds for the DMB labeling experiments. For the synthesis of Ac_4_ManNCyc(H_2_), we activated the free acid **1** with *N*-hydroxysuccinimide (NHS) and *N,N'*-dicyclohexylcarbodiimide (DCC) to obtain active ester **3**. The synthesis of Ac_4_ManNCp(H_2_) started from the commercially available activated cyclopropane **2**. In the next step, mannosamine hydrochloride (ManN·HCl) was neutralized with Hünig’s base (diisopropylethylamine, DIPEA) in DMF and reacted with the activated cyclopropene derivatives, followed by peracetylation with acetic anhydride in pyridine. Ac_4_ManNCp(H_2_) could be obtained in 34% yield and Ac_4_ManNCyc(H_2_) in 52% yield. Since the stereoisomers resulting from the chiral centers at the methylcyclopropyl (and also methylcyclopropenyl) residues were not readily separable, we always used mixtures of isomers. Small amounts of the ManN derivatives were deacetylated with sodium methoxide in methanol and a subsequent sialic acid aldolase reaction delivered the corresponding sialic acids. After RP-HPLC purification, they were labeled with DMB and the final reference compounds were analyzed by RP-HPLC-MS (Figures S2 and S3, Supporting Information File 1).

**Scheme 3:**
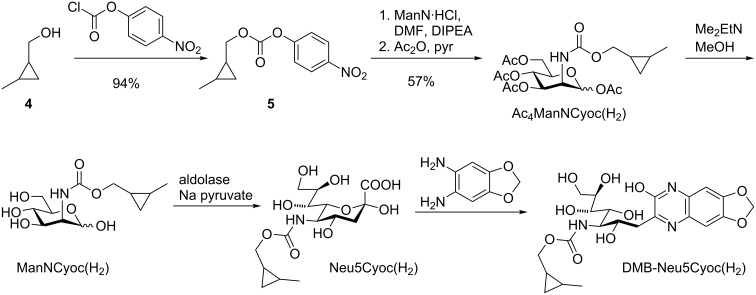
Synthesis of Ac_4_ManNCyoc(H_2_) and the corresponding DMB-labeled sialic acid.

The synthesis of cyclopropane derivative Ac_4_ManNCyoc(H_2_) is shown in Scheme 3. Alcohol **4** was activated with 4-nitrophenyl chloroformate, and the obtained carbonate **5** reacted with neutralized mannosamine and peracetylated as described above to give Ac_4_ManNCyoc(H_2_) in a yield of 57%. Deacetylation with *N*,*N*-ethyldimethylamine in methanol and further aldolase reaction and DMB labeling gave reference compound DMB-Neu5Cyoc(H_2_) that was analyzed by RP-HPLC-MS (Figure S4, Supporting Information File 1). Additionally, we synthesized the literature known DMB derivatives of the natural sialic acid Neu5Ac [29] and of sodium pyruvate [30] as reference compounds to determine their retention times with the chosen gradients (Figures S5–S8, Supporting Information File 1).

We next performed MGE experiments with cyclopropane derivatives. HEK 293T cells were grown with Ac_4_ManNCyc(H_2_), Ac_4_ManNCp(H_2_), Ac_4_ManNCyoc(H_2_), or DMSO only (solvent control) for two days. Subsequently, cells were harvested and treated with acetic acid to cleave the sialic acids. These were then labeled with DMB and analyzed by RP-HPLC using a fluorescence detector (*λ*_ex_ = 372 nm, *λ*_em_ = 456 nm) (for solvent control see Figures S9 and S10, Supporting Information File 1). Both amide-linked derivatives were efficiently incorporated into cellular sialic acids (Ac_4_ManNCyc(H_2_): *IE* = (50.0 ± 2.1)%, Ac_4_ManNCp(H_2_): *IE* = (71.7 ± 12.8)%) (Figures S11 and S12, Supporting Information File 1). This demonstrates that the additional methyl group has a significant impact on the incorporation efficiency although that of Ac_4_ManNCyc(H_2_) is still very high. However, as indicated above, it has to be kept in mind that a methylcyclopropane has an angled structure in contrast to methylcyclopropene. For Ac_4_ManNCyoc(H_2_) an incorporation efficiency of only (4.9 ± 1.9)% was determined (Figure S13, Supporting Information File 1) showing that this larger modification is much less well accepted by the enzymatic machinery. The different incorporation efficiencies of Ac_4_ManNCp(H_2_) and Ac_4_ManNCyoc(H_2_) readily explain our observation that the corresponding cyclopropene derivatives result is a similar staining intensity (Figure 3). Obviously, the lower DAinv reactivity of Ac_4_ManNCp is compensated by its higher incorporation efficiency.

### MGE with Ac_4_GlcNCp and Ac_4_GalNCp

**Scheme 4:**
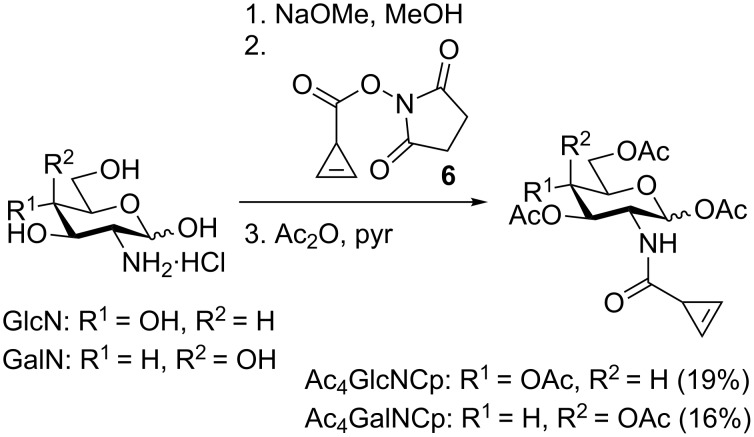
Synthesis of Ac_4_GlcNCp and Ac_4_GalNCp.

Recently, the investigation of intracellular glycoproteins gained increasing attention. Therefore, the development of glucosamine and galactosamine derivatives suitable for MGE is of high importance. Until now, the carbamate-linked methylcyclopropenes Ac_4_GlcNCyoc and Ac_4_GalNCyoc are the only cyclopropene derivatives that were examined in this context [25–26]. Ac_4_GlcNCyoc was used to visualize protein-specific glycosylation inside living cells [32]. However, this compound is cytotoxic when applied in higher concentrations. Thus, novel glucosamine derivatives with improved properties would be beneficial. Based on the findings described above, especially the excellent incorporation efficiency of Ac_4_ManNCp(H_2_), we hypothesized, that also the corresponding glucosamine derivative Ac_4_GlcNCp might be better incorporated than Ac_4_GlcNCyoc. Consequently, we synthesized Ac_4_GlcNCp and Ac_4_GalNCp (Scheme 4). Glucosamine hydrochloride and galactosamine hydrochloride, respectively, were neutralized with sodium methoxide and then reacted with activated cyclopropene **6** followed by peracetylation. Ac_4_GlcNCp was obtained in 19% yield and Ac_4_GalNCp in 16% yield over two steps.

**Figure 4:**
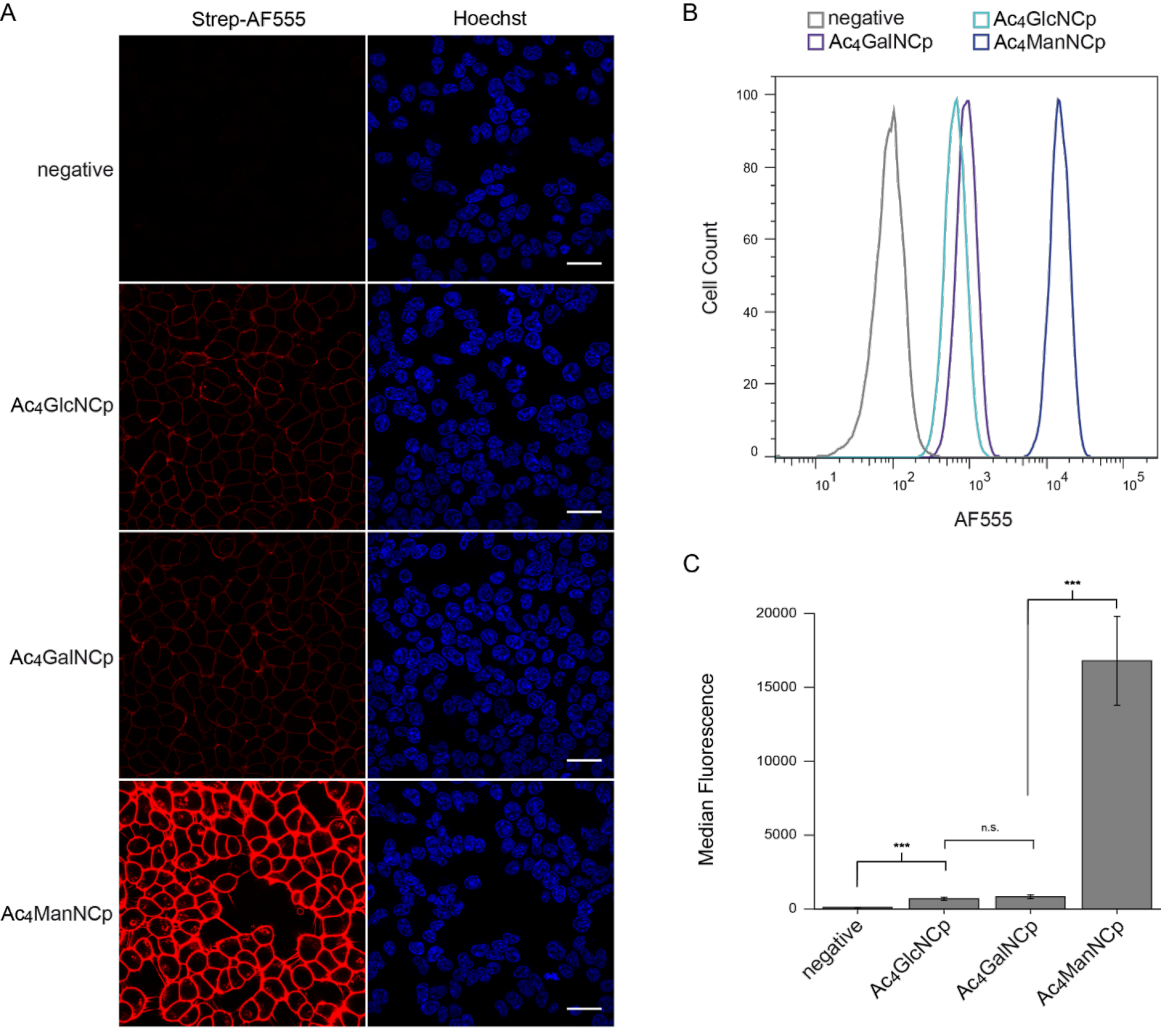
HEK 293T cells were grown with 100 μM Ac_4_ManNCp, Ac_4_GlcNCp, Ac_4_GalNCp or DMSO only (negative control) for 48 h. Cells were incubated with Tz-biotin (A: 500 μM, B/C: 100 μM) for 3 h (A) or 30 min (B/C) at 37 °C followed by incubation with strep-AF555. A) Results from confocal fluorescence microscopy. Nuclei were stained with Hoechst 33342. Scale bar: 30 μm. B) Histogram from flow cytometry experiments. C) Median fluorescence from three independent flow cytometry experiments.

**Figure 5:**
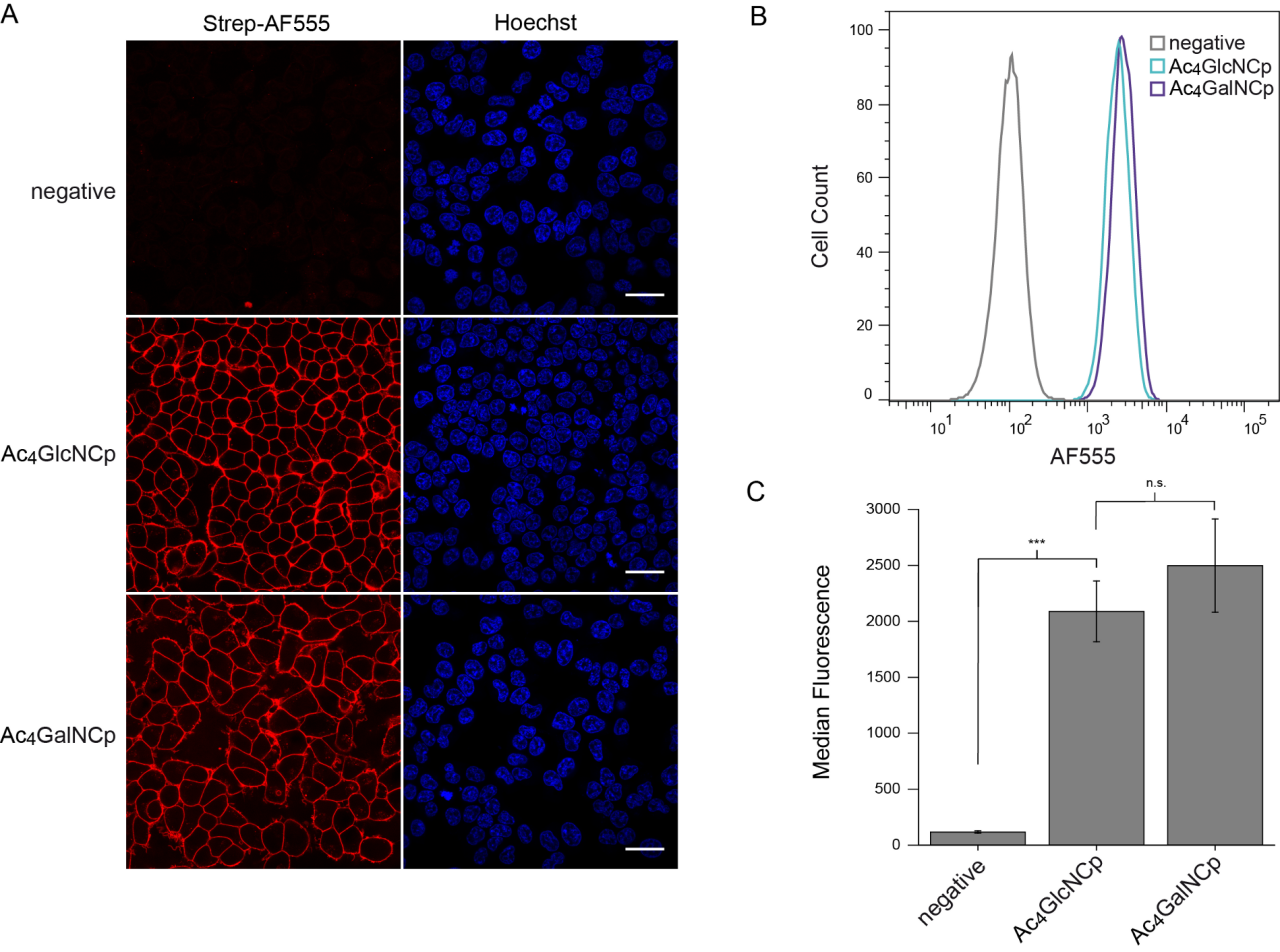
HEK 293T cells were grown with 100 μM Ac_4_GlcNCp, Ac_4_GalNCp or DMSO only (negative control) for 48 h. Cells were incubated with Tz-biotin (500 μM) for 3 h (A) or 1 h (B/C) at 37 °C followed by incubation with strep-AF555. A) Results from confocal fluorescence microscopy. Nuclei were stained with Hoechst 33342. Scale bar: 30 μm. B) Histogram from flow cytometry experiments. C) Median fluorescence from three independent flow cytometry experiments.

We next explored the suitability of Ac_4_GlcNCp and Ac_4_GalNCp in MGE. Applying the same protocol used for the mannosamine derivatives, we first performed fluorescence microscopy experiments after MGE. As a positive control, we included Ac_4_ManNCp to enable comparison studies. The microscopy images showed a distinct membrane staining for Ac_4_GlcNCp and Ac_4_GalNCp, that was clearly weaker than that for Ac_4_ManNCp (Figure 4). These results are similar to those obtained with the Cyoc-sugars [25–26]. Adjustment of the reaction conditions and microscopy settings resulted in a bright staining for Ac_4_GlcNCp and Ac_4_GalNCp well over that of the negative control (Figure 5). These results were confirmed by flow cytometry (Figure 4 and Figure 5). Interestingly, we did not observe cytotoxicity of Ac_4_GlcNCp up to a concentration of 100 μM.

### Comparison of glucosamine and galactosamine derivatives

**Figure 6:**
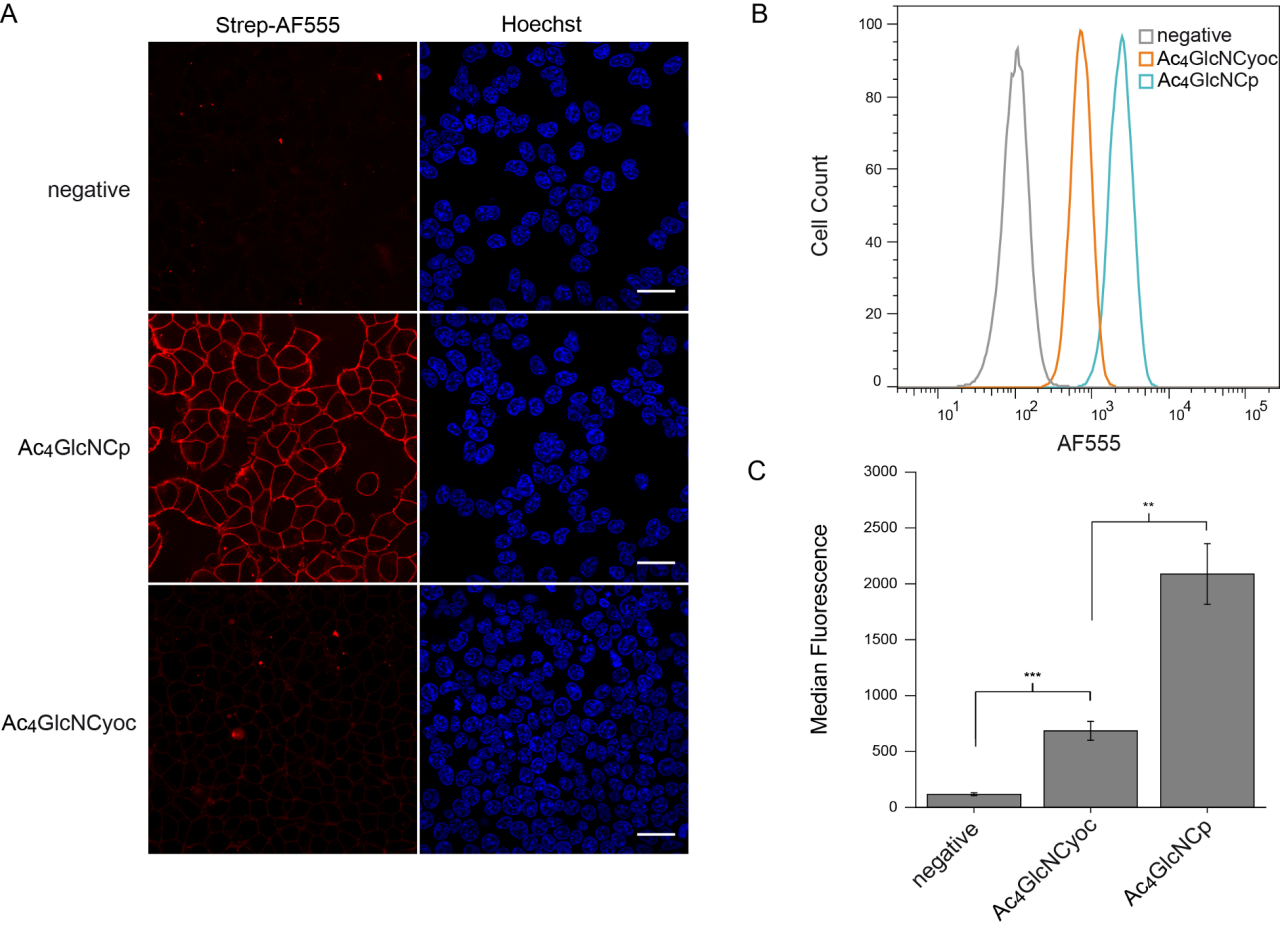
HEK 293T cells were grown with 50 μM (A) or 100 μM (B) Ac_4_GlcNCp, Ac_4_GlcNCyoc or DMSO only (negative control) for 48 h. Cells were incubated with Tz-biotin (500 μM) for 3 h (A) or 1 h (B/C) at 37 °C followed by incubation with strep-AF555. A) Results from confocal fluorescence microscopy. Nuclei were stained with Hoechst 33342. Scale bar: 30 μm. B) Histogram from flow cytometry experiments. C) Median fluorescence from three independent flow cytometry experiments.

Having proven the suitability of Ac_4_GlcNCp for MGE, we next compared it with Ac_4_GlcNCyoc. First, we investigated the staining intensity on the cell surface by confocal fluorescence microscopy. Owing to the cytotoxicity of Ac_4_GlcNCyoc, a concentration of 50 μM was used for both sugars. In contrast to the corresponding mannosamine derivatives, Ac_4_GlcNCp resulted in a much brighter staining compared to Ac_4_GlcNCyoc (Figure 6A). Flow cytometry experiments confirmed these results and revealed that the median fluorescence of Ac_4_GlcNCp is three times that of Ac_4_GlcNCyoc (Figure 6B,C).

**Figure 7:**
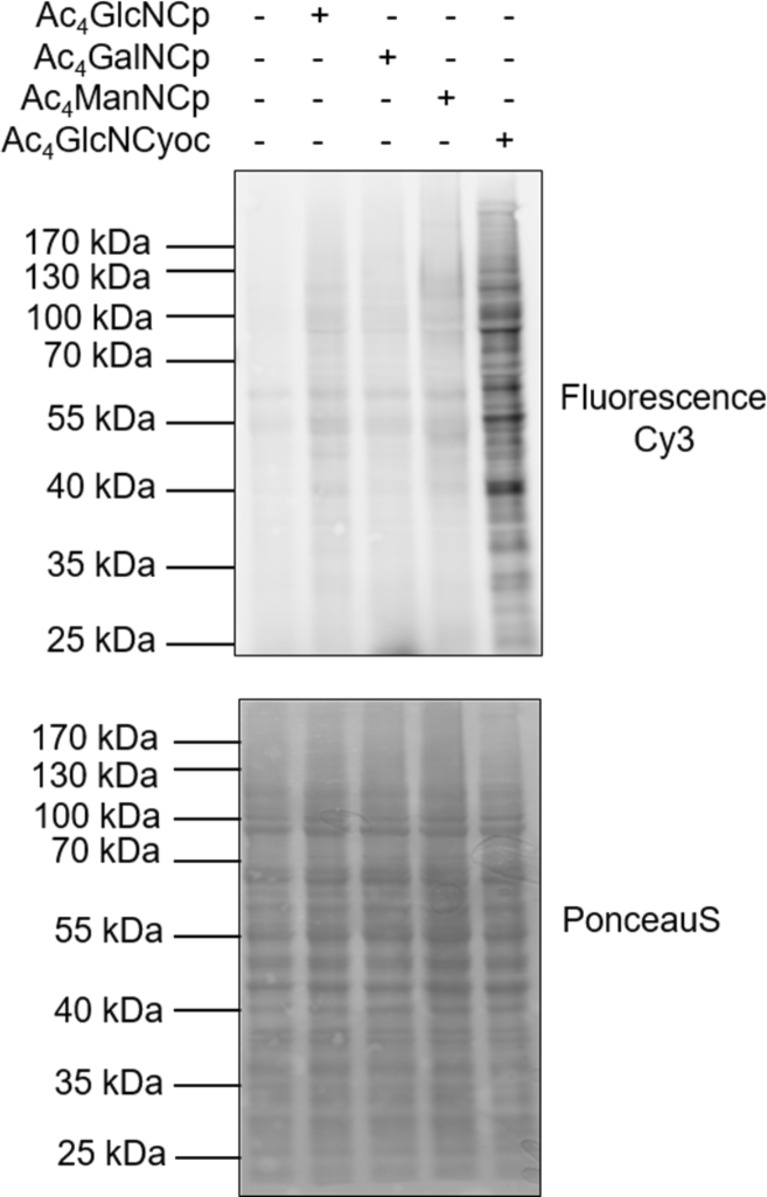
Western blot analysis of soluble glycoproteins. HEK 293T cells were grown for 48 h with 100 μM Ac_4_ManNCp, Ac_4_GlcNCp, Ac_4_GalNCp, Ac_4_GlcNCyoc or DMSO only (negative control), lysed, and the cleared lysate was reacted with Tz-Cy3 (10 μM, 90 min, 24 °C). Ponceau S staining was used as loading control.

MGE with glucosamine and galactosamine derivatives is of interest to investigate O-GlcNAcylation of intracellular glycoproteins [32–35]. To include intracellular proteins in our analysis, we performed Western blot analysis of cell lysates. HEK 293T cells were cultivated with Ac_4_ManNCp, Ac_4_GlcNCp, Ac_4_GalNCp, or Ac_4_GlcNCyoc for 48 h. Subsequently, cells were harvested, lysed and the lysate was cleared by centrifugation resulting in an enrichment of soluble proteins. After labeling with Tz-Cy3 the proteins were separated by gel electrophoresis and blotted. Equal protein loading was verified by Ponceau S staining. As observed earlier [26], Ac_4_GlcNCyoc resulted in a significant staining of proteins (Figure 7). In contrast, Ac_4_GlcNCp as well as the mannosamine and galactosamine derivatives showed only weakly labeled protein bands. The observation that Ac_4_GlcNCyoc results in stronger staining of soluble proteins than Ac_4_GlcNCp whereas Ac_4_GlcNCp gives a stronger cell surface staining suggests that Ac_4_GlcNCyoc is better accepted by the enzymes producing intracellular glycoproteins while Ac_4_GlcNCp is better accepted by the enzymes involved in the biosynthesis of membrane glycoconjugates. However, many processes are responsible for the staining intensity of either intracellular or cell-surface proteins including cellular uptake of the carbohydrate derivative used for MGE, its metabolization, transport, speed of the ligation reaction, and the occurrence of alternative glycosylation pathways [36]. Since the elucidation of the exact background of our observation requires an in-depth analysis far beyond the scope of this article, we focus here on one of these aspects, i.e., the conversion of glucosamine into mannosamine derivatives resulting in a possible increase of the staining intensity on the cell surface.

### Are Ac_4_GlcNCyoc and Ac_4_GlcNCp converted into sialic acids during MGE?

**Scheme 5:**
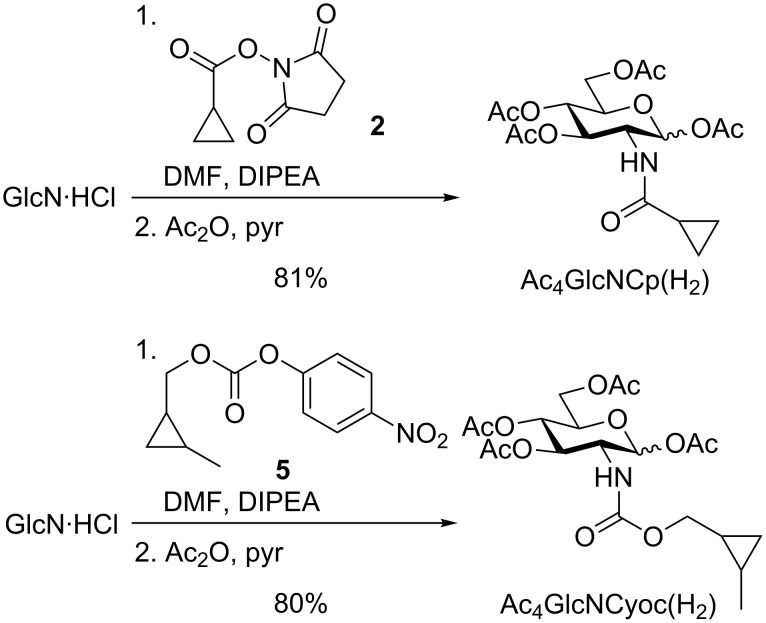
Synthesis of Ac_4_GlcNCp(H_2_) and Ac_4_GlcNCyoc(H_2_).

It is well established that carbohydrate derivatives can be interconverted into each other by epimerases. For example, both GlcNAc and UDP-GlcNAc can be converted to ManNAc [37–38] thereby joining the sialic acid biosynthesis pathway. Thus, a possible explanation of the staining of cell surfaces after MGE with glucosamine derivatives is their conversion into sialic acid derivatives and further into sialo glycoconjugates. To investigate this possibility, we carried out MGE experiments with the cyclopropane derivatives Ac_4_GlcNCp(H_2_) and Ac_4_GlcNCyoc(H_2_) followed by DMB labeling of sialic acids. Their synthesis started from glucosamine hydrochloride (Scheme 5) as described for the mannosamine analogues. After MGE with Ac_4_GlcNCp(H_2_) followed by DMB labeling we found that (3.5 ± 0.4)% of the sialic acids are modified as Neu5Cp(H_2_) (Figure S14, Supporting Information File 1). After MGE with Ac_4_GlcNCyoc(H_2_) on the other hand we could not detect the corresponding sialic acid on the cell surface (Figure S15, Supporting Information File 1). Thus, the cell surface staining observed after MGE with Ac_4_GlcNCp could at least in part be caused by the corresponding sialic acid Neu5Cp being a possible explanation for the higher staining intensity obtained with Ac_4_GlcNCp compared to Ac_4_GlcNCyoc.

## Conclusion

Cyclopropene derivatives have proven to be suitable chemical reporter groups for MGE. In this investigation, we compared various aminosugar derivatives carrying three different cyclopropene moieties for this purpose. The Cyc and Cp residues, which differ by the presence or absence of a methyl group at the double bond, are connected by an amide-linkage to the aminosugar. The Cyoc moiety is connected by a carbamate-linkage. All three cyclopropene derivatives easily undergo DAinv reactions. Kinetic studies revealed that the carbamate derivative ManNCyoc has the highest reaction rate, followed by ManNCp and finally ManNCyc with the slowest reaction kinetics. Performing MGE experiments with the mannosamine derivatives followed by visualization of cell-surface labeling using the DAinv reaction demonstrated that Ac_4_ManNCyc produced only a weak staining, whereas Ac_4_ManNCp and Ac_4_ManNCyoc yielded in a comparably strong staining. Obviously, the lower DAinv reactivity of the Cp derivative in comparison to the Cyoc derivative is compensated by its high metabolic acceptance as suggested by investigation of the corresponding cyclopropane derivatives. Previously, it was speculated that the lower staining intensity obtained with Ac_4_ManNCyc in comparison to Ac_4_ManNCyoc is caused in part by its low incorporation efficiency due to branching in the α-position of the carbonyl group [25]. Our results with the corresponding cyclopropane derivatives, however, indicate that the main reason for the low labeling intensity is the sluggish DAinv reactivity of the Cyc reporter.

Based on the high incorporation efficiency of Ac_4_ManNCp, we synthesized two new derivatives, Ac_4_GlcNCp and Ac_4_GalNCp, which are both suitable for MGE resulting in cell-surface staining of comparable intensity. Interestingly, Ac_4_GlcNCp – in contrast to Ac_4_GlcNCyoc – showed only weakly labeled protein bands in a Western blot whereas its staining intensity on the cell surface was considerably stronger. MGE experiments with the cyclopropane analogs and subsequent DMB labeling of cellular sialic acids suggest that the amide-linked Ac_4_GlcNCp but not the carbamate-linked Ac_4_GlcNCyoc is converted to the corresponding sialic acid thus contributing to cell-surface labeling. In conclusion, we expanded the MGE toolbox by novel cyclopropene-modified glucosamine and galactosamine derivatives that offer interesting options for metabolic labeling.

## Experimental

### General methods

Ac_4_ManNCyc [23], Ac_4_ManNCp [27], Ac_4_ManNCyoc [24–25], and Ac_4_GlcNCyoc [25–26] were synthesized according to published procedures. AlexaFluor 555-labeled streptavidin and Hoechst 33342 were purchased from Invitrogen. Reactions were monitored by TLC using aluminum sheets pre-coated with silica gel 60 F254 (Merck) with detection by UV light (λ = 254 nm). Additionally, acidic ethanolic *p*-anisaldehyde solution or basic KMnO_4_ solution, followed by gentle heating, were used for visualization. Preparative column chromatography was performed by flash column chromatography using silica gel 60 M from Macherey-Nagel or with an MPLC-Reveleris X2 system (Büchi). NMR spectra were recorded at room temperature with an Avance III 400 or an Avance III 600 instrument from Bruker. Chemical shifts are reported in ppm relative to solvent signals (CDCl_3_: δ_H_ = 7.26 ppm, δ_C_ = 77.16 ppm). Signal assignments were carried out by two-dimensional ^1^H,^1^H and ^1^H,^13^C correlation spectroscopy (COSY, HSQC, and HMBC). Analytical RP-HPLC-MS was performed on an LCMS2020 prominence system (pumps LC-20AD, column oven CTO-20AC, UV–vis detector SPD-20A, RF-20A Prominence fluorescence detector (λ_ex_ = 372 nm, λ_em_ = 456 nm), controller CBM-20A, ESI detector, software LC-solution) from Shimadzu under the following conditions. Column: EC125/4 Nucleodur C_18_ from Macherey-Nagel, flow: 0.4 mL min^−1^; mobile phase: gradient of acetonitrile with 0.1% formic acid (solvent B) in water with 0.1% formic acid (solvent A). Semi-preparative HPLC was performed on a LC20A Prominence system (high-pressure pumps LC-20AT, auto sampler SIL-20A, column oven CTO-20AC, diode array detector SPDM20A, controller CBM-20A, software LC-solution) from Shimadzu under the following conditions. Column: Nucleodur 100-5 C18ec from Macherey Nagel (21.1 × 250 mm), flow: 9 mL min^−1^, mobile phase: gradient of acetonitrile with 0.1% formic acid (solvent B) in water with 0.1% formic acid (solvent A). UV–vis absorption for kinetic measurements was measured with a Cary 50 instrument from Varian and Cary WinUV scanning kinetics software. High-resolution mass spectra (HRMS) were recorded on a micrOTOF II instrument from Bruker in positive and negative mode. The ionization method was electrospray (ESI) and for detection the time of flight (TOF) method was used. Analysis of recorded mass spectra was performed using the software Xcalibur by Thermo Fischer Scientific*.*

**2,5-Dioxopyrrolidin-1-yl 2-methylcyclopropane-1-carboxylate (3):** *N*-Hydroxysuccinimide (16.09 g, 139.83 mmol) and *N,N'*-dicyclohexylcarbodiimide (24.73 g, 119.86 mmol) were dissolved under nitrogen atmosphere in dry THF (300 mL). 2-Methylcyclopropanecarboxylic acid (**1**, 10.0 g, 99.88 mmol) was added and the milky reaction mixture was stirred overnight. The precipitate was filtered off and the filtrate evaporated under reduced pressure. The crude product was purified by column chromatography (petroleum ether/ethyl acetate 2:1) to obtain **3** as a white solid (13.75 g, 70%) as a mixture of isomers (indicated as a and b). *R*_f_ = 0.39 (petroleum ether/ethyl acetate 2:1); ^1^H NMR (400 MHz, CDCl_3_) δ 2.81 (s, 4H, CH_2_CH_2_), 1.99–1.90 (m, 1H, C(O)CH-b), 1.68–1.52 (m, 2H, C*H*CH_3_, C(O)CH-a), 1.42–1.35 (m, 1H, CH_2_-a), 1.32–1.26 (m, 1H, CH_2_-b), 1.23 (d, *J* = 6.2 Hz, 3H, CH_3_-b), 1.19 (d, *J* = 5.7 Hz, 3H, CH_3_-a), 1.09–1.00 (m, 1H, CH_2_-b), 1.00–0.91 (m, 1H, CH_2_-a); ^13^C NMR (101 MHz, CDCl_3_) δ 169.6 (C=O), 169.3 (C=O), 168.1 (C=O), 25.6 (CH_2_CH_2_), 19.8 (CH_2_-a), 18.8 (*C*HCH_3_), 18.4 (CH_2_-b), 18.1 (CH_3_-a), 17.7 (C(O)*C*H-a), 16.6 (CH_2_-b), 15.7 (C(O)*C*H-b), 12.0 (CH_3_-b).

**Ac_4_ManNCp(H_2_):** Mannosamine hydrochloride (500 mg, 2.32 mmol) was suspended under a nitrogen atmosphere in dry DMF (10 mL) and diisopropylethylamine (1.1 mL, 6.33 mmol) was added. After 1 h, 2,5-dioxopyrrolidin-1-yl cyclopropanecarboxylate (386 mg, 2.11 mmol) was added and the reaction mixture was stirred at room temperature for 3 days. The solvent was removed under reduced pressure and the residue dissolved in pyridine (2 mL) and acetic anhydride (2 mL). After two days at room temperature, the solvents were removed under reduced pressure and coevaporated with ethanol. The brown residue was dissolved in dichloromethane (20 mL) and washed with 10% aq KHSO_4_ (1 × 20 mL), sat. aq NaHCO_3_ (1 × 20 mL) and brine (1 × 20 mL). The organic layer was dried over MgSO_4_ and the solvent removed under reduced pressure. The crude product was purified by column chromatography (petroleum ether/ethyl acetate 1:1) to yield Ac_4_ManNCp(H_2_) (302 mg, 34%) as a mixture of anomers as a colorless solid. Whereas the α-anomer could be partially separated by column chromatography, semi-preparative RP-HPLC (50–65% B over 20 min) was required to obtain pure β-anomer (*t*_R_ = 10.0 min). *R*_f_ = 0.50 (petroleum ether/ethyl acetate 1:2); α-isomer: ^1^H NMR (400 MHz, CDCl_3_) δ 6.05 (d, *J* = 1.9 Hz, 1H, H-1), 5.87 (d, *J* = 9.2 Hz, 1H, NH), 5.32 (dd, *J* = 10.2, 4.5 Hz, 1H, H-3), 5.21 (‘t‘, *J* = 10.2 Hz, 1H, H-4), 4.67 (ddd, *J* = 9.2, 4.5, 1.9 Hz, 1H, H-2), 4.29 (dd, *J* = 12.6, 5.0 Hz, 1H, H-6), 4.14–3.97 (m, 2H, H-5, H-6), 2.17 (s, 3H, OAc), 2.10 (s, 3H, OAc), 2.07 (s, 3H, OAc), 1.98 (s, 3H, OAc), 1.49–1.36 (m, 1H, CH), 1.06–0.89 (m, 2H, CH_2_), 0.91–0.74 (m, 2H, CH_2_); ^13^C NMR (101 MHz, CDCl_3_) δ 173.9 (C=O), 170.7 (C=O), 170.1 (C=O), 169.9 (C=O), 168.3 (C=O), 92.0 (C-1), 70.3 (C-5), 69.1 (C-3), 65.7 (C-4), 62.3 (C-6), 49.5 (C-2), 21.0 (OAc), 20.9 (OAc), 20.8 (OAc), 20.8 (OAc), 14.9 (CH), 8.2 (CH_2_), 8.1 (CH_2_); β-isomer: ^1^H NMR (400 MHz, CDCl_3_) δ 5.98 (d, *J* = 9.1 Hz, 1H, NH), 5.90–5.77 (m, 1H, H-1), 5.15 (“t“, *J* = 9.8 Hz, 1H, H-4), 5.03 (ddd, *J* = 9.9, 4.1, 1.3 Hz, 1H, H-3), 4.85–4.73 (m, 1H, H-2), 4.29 (dd, *J* = 12.4, 5.3 Hz, 1H, H-6), 4.10 (dd, *J* = 12.6, 2.0 Hz, 1H, H-6), 3.86–3.72 (m, 1H, H-5), 2.10 (s, 3H, OAc), 2.09 (s, 3H, OAc), 2.06 (s, 3H, OAc), 1.96 (s, 3H, OAc), 1.54–1.39 (m, 1H, CH), 1.04–0.90 (m, 2H, CH_2_), 0.90–0.67 (m, 2H, CH_2_); ^13^C NMR (101 MHz, CDCl_3_) δ 174.4 (C=O), 170.7 (C=O), 170.2 (C=O), 169.9 (C=O), 168.5 (C=O), 90.9 (C-1), 73.6 (C-5), 71.5 (C-3), 65.6 (C-4), 62.1 (C-6), 49.6 (C-2), 20.9 (OAc), 20.9 (OAc), 20.8 (OAc), 20.8 (OAc), 15.0 (CH), 8.1 (CH_2_), 7.9 (CH_2_); HRMS *m/z*: [M + Na]^+^ calcd for C_18_H_25_NO_10_, 438.1371; found, 438.1366.

**Ac_4_ManNCyc(H_2_)**: Mannosamine hydrochloride (500 mg, 2.32 mmol) was suspended under nitrogen atmosphere in dry *N*,*N*-dimethylformamide (10 mL) and diisopropylethylamine (1.1 mL, 6.33 mmol) was added. After 1 h the activated cyclopropane **3** (416 mg, 2.11 mmol) was added and the reaction mixture was stirred at room temperature for 3 days. The solvent was removed under reduced pressure and the residue dissolved in pyridine (2 mL) and acetic anhydride (2 mL). After two days at room temperature, the solvents were removed under reduced pressure and coevaporated with ethanol. The brown residue was dissolved in dichloromethane (20 mL) and washed with 10% aq KHSO_4_ (1 × 20 mL), sat. aq NaHCO_3_ (1 × 20 mL) and brine (1 × 20 mL). The organic layer was dried over MgSO_4_ and the solvent removed under reduced pressure. The crude product was purified by column chromatography (petroleum ether/ethyl acetate 1:1) to yield Ac_4_ManNCyc(H_2_) (473 mg, 52%) as a mixture of isomers (anomers as well as cyclopropane isomers indicated as a and b) as a colorless solid. Whereas the α-anomers could be partially separated by column chromatography, semi-preparative RP-HPLC (50–55% B over 20 min) was required to obtain β-anomers (*t*_R_ = 12.3 min). *R*_f_ = 0.54 (petroleum ether/ethyl acetate 1:2); α-isomer: ^1^H NMR (400 MHz, CDCl_3_) δ 6.07 (d, *J* = 1.8 Hz, 1H, H-1a), 6.04 (d, *J* = 1.9 Hz, 1H, H-1b), 5.86–5.72 (m, 1H, NH), 5.36–5.28 (m, 1H, H-3), 5.28–5.12 (m, 1H, H-4), 4.69–4.57 (m, 1H, H-2), 4.36–4.25 (m, 1H, H-6), 4.12–3.94 (m, 2H, H-5, H-6), 2.17 (s, 3H, OAc), 2.11 (s, 3H, OAc), 2.07 (s, 3H, OAc), 1.99 (s, 3H, OAc-b), 1.97 (s, 3H, OAc-a), 1.47–1.27 (m, 1H, C(O)CH), 1.20–1.06 (m, 5H, CHC*H*_3_,CH_2_), 0.74–0.53 (m, 1H, C*H*CH_3_); ^13^C NMR (101 MHz, CDCl_3_) δ 173.59 (C=O), 173.56 (C=O), 170.7 (C=O), 170.1 (C=O), 169.94 (C=O), 169.88 (C=O), 168.3 (C=O), 92.0 (C-1a), 91.97 (C-1b), 70.3 (C-5), 69.05 (C-3a), 69.02 (C-3b), 65.8 (C-4b), 65.7 (C-4a), 62.30 (C-6b), 62.26 (C-6a), 49.52 (C-2b), 49.45 (C-2a), 23.6 (CH_2_-a), 23.50 (CH_2_-b), 21.0 (OAc), 20.89 (OAc), 20.9 (OAc), 20.82 (OAc), 20.80 (OAc), 18.03 (CH*C*H_3_-a), 18.01 (CH*C*H_3_-b), 16.8 (*C*HCH_3_-b), 16.59 (*C*HCH_3_-a), 16.56 (C(O)*C*H-a), 16.52 (C(O)*C*H-b); β-isomer: ^1^H NMR (400 MHz, CDCl_3_) δ 5.96–5.87 (m, 1H, NH), 5.87–5.79 (m, 1H, H-1), 5.18–5.09 (m, 1H, H-4), 5.08–4.97 (m, 1H, H-3), 4.80–4.70 (m, 1H, H-2), 4.33–4.17 (m, 1H, H-6), 4.15–4.05 (m, 1H, H-6), 3.83–3.71 (m, 1H, H-5), 2.12–2.08 (m, 6H, OAc), 2.06–2.03 (m, 3H, OAc), 1.97 (s, 3H, OAc-a), 1.95 (s, 3H, OAc-b), 1.39–1.27 (m, 1H, C(O)CH), 1.24–1.06 (m, 5H, CHC*H*_3_,CH_2_), 0.68–0.53 (m, 1H, C*H*CH_3_); ^13^C NMR (101 MHz, CDCl_3_) δ 174.13 (C=O), 174.09 (C=O), 170.6 (C=O), 170.2 (C=O), 168.5 (C=O), 90.9 (C-1), 73.6 (C-5), 71.5 (C-3b), 71.4 (C-3a), 65.65 (C-4a), 65.57 (C-4b), 62.2 (C-6a), 62.1 (C-6b), 49.6 (C-2a), 49.5 (C-2b), 23.6 (CH_2_-b), 23.5 (CH_2_-a), 20.92 (OAc), 20.89 (OAc), 20.84 (OAc), 20.79 (OAc), 20.75 (OAc), 18.05 (CH*C*H_3_-a), 17.99 (CH*C*H_3_-b), 16.5, 16.4, 16.2 (C(O)*C*H, *C*HCH_3_); HRMS *m/z*: [M + Na]^+^ calcd for C_19_H_27_NO_10_, 452.1527; found, 452.1522.

**(2-Methylcyclopropyl)methyl (4-nitrophenyl) carbonate (5):** 2-Methylcyclopropanemethanol (**4**, 0.57 mL, 5.81 mmol) was dissolved under nitrogen atmosphere in dry dichloromethane (80 mL) and dry pyridine (2.8 mL). The solution was cooled to 4 °C and 4-nitrophenyl chloroformate (2.57 g, 12.77 mmol) was added. After 18 h at room temperature, the reaction mixture was diluted with water until complete solution of the precipitate. The aqueous phase was extracted with dichloromethane, the organic phases were combined, dried over MgSO_4_ and the solvent removed under reduced pressure. The crude product was purified by silica gel chromatography (petroleum ether/ethyl acetate 5:1) and **5** (1.37 g, 94%) was obtained as a mixture of isomers (indicated as a and b) as a colorless liquid. *R*_f_ = 0.70 (petroleum ether/ethyl acetate 5:1); ^1^H NMR (400 MHz, CDCl_3_) δ 8.32–8.12 (m, 2H, H_ar_), 7.53–7.28 (m, 2H, H_ar_), 4.52–4.39 (m, 2H, OCH_2_-b), 4.21–3.96 (m, 2H, OCH_2_-a), 1.30–1.19 (m, 1H, CH_2_C*H*-b), 1.14 (d, *J* = 6.2 Hz, 3H, CH_3_-b), 1.09 (d, *J* = 6.0 Hz, 1H, CH_3_-a), 1.07–1.02 (m, 1H, CH_2_-b), 1.01–0.91 (m, 1H, CH_2_C*H*-a), 0.89–0.72 (m, 1H, CH_3_C*H*), 0.59–0.50 (m, 1H, CH_2_-a), 0.49–0.36 (m, 1H, CH_2_-a), 0.16–0.08 (m, 1H, CH_2_-b); ^13^C NMR (101 MHz, CDCl_3_) δ 155.7 (C_quart_), 152.6 (C_quart_), 145.3 (C_quart_), 125.3 (C_ar_), 121.8 (C_ar_), 74.0 (OCH_2_), 70.7 (OCH_2_), 18.2 (CH_3_-a), 17.9 (CH_2_*C*H-a), 14.0 (CH_2_*C*H-b), 13.3 (CH_3_-b), 12.0 (CH_2_-a), 11.7 (CH_3_*C*H-a), 11.2 (CH_3_*C*H-b), 10.3 (CH_2_-b).

**Ac_4_ManNCyoc(H_2_):** Mannosamine hydrochloride (500 mg, 2.32 mmol) was suspended under nitrogen atmosphere in dry *N*,*N*-dimethylformamide (10 mL) and diisopropylethylamine (1.1 mL, 6.33 mmol) was added. After 20 min the activated cyclopropane **5** (530 mg, 2.11 mmol) was added and the reaction mixture was stirred at room temperature for 4 days. The solvent was removed under reduced pressure and the residue dissolved in pyridine (2 mL) and acetic anhydride (2 mL). After 2 days at room temperature, the solvents were removed under reduced pressure and coevaporated with ethanol. The brown residue was dissolved in dichloromethane (25 mL) and washed with 10% aq KHSO_4_ (1 × 25 mL), sat. aq NaHCO_3_ (1 × 25 mL) and brine (1 × 25 mL). The organic layer was dried over MgSO_4_ and the solvent removed under reduced pressure. The crude product was purified by column chromatography (petroleum ether/ethyl acetate 2:1) to yield Ac_4_ManNCyoc(H_2_) (550 mg, 57%) as a colorless solid. Anomers could be separated by column chromatography and were obtained as isomeric mixtures (indicated as a and b). *R*_f_ = 0.38 (petroleum ether/ethyl acetate 3:2); α-isomer: ^1^H NMR (400 MHz, CDCl_3_) δ 6.14–6.02 (m, 1H, H-1), 5.29 (dd, *J* = 10.3, 4.3 Hz, 1H, H-3), 5.19 (‘t‘, *J* = 10.1 Hz, 1H, H-4), 5.10 (d, *J* = 9.2 Hz, 1H, NH), 4.36–4.29 (m, 1H, H-2), 4.24 (dd, *J* = 12.3, 4.5 Hz, 1H, H-6), 4.09–3.99 (m, 2H, H-6, H-5), 3.97–3.79 (m, 2H, OCH_2_), 2.16 (s, 3H, OAc), 2.09 (s, 3H, OAc), 2.04 (s, 3H, OAc), 2.00 (s, 3H, OAc), 1.12–0.96 (m, 4H, CHC*H*_3_, OCH_2_C*H*-b), 0.99–0.90 (m, 1H, C*H*CH_3_-b), 0.89–0.78 (m, 1H, OCH_2_C*H*-a), 0.78–0.73 (m, 1H, CH_2_-b), 0.72–0.64 (m, 1H, C*H*CH_3_-a), 0.49–0.39 (m, 1H, CH_2_-a), 0.37–0.24 (m, 1H, CH_2_-a), 0.02–0.04 (m, 1H, CH_2_-b); ^13^C NMR (101 MHz, CDCl_3_) δ 170.6 (C=O), 170.1 (C=O), 169.6 (C=O), 168.1 (C=O), 156.2 (C=O), 91.9 (C-1), 70.2, 70.0 (C-5 and OCH_2_), 69.2 (C-3), 65.4 (C-4), 62.0 (C-6), 51.1 (C-2), 20.9 (OAc), 20.7 (OAc), 20.6 (OAc), 18.4, 18.3 (CH*C*H_3_-a and OCH_2_*C*H),13.2 (CH*C*H_3_-b), 11.63, 11.61, 11.55, 11.48 (*C*HCH_3_-a and CH_2_), 10.0 (CHCH_3_-b). β-isomer: ^1^H NMR (400 MHz, CDCl_3_) δ (ppm) 5.84 (d, *J* = 1.9 Hz, 1H, H-1), 5.21–5.08 (m, 2H, NH, H-4), 5.02 (dd, *J* = 9.8, 3.8 Hz, 1H, H-3), 4.51–4.43 (m, 1H, H-2), 4.29–4.19 (m, 1H, H-6, OCH_2_-b), 4.10 (dd, *J* = 12.4, 2.5 Hz, 1H, H-6), 4.04–3.84 (m, 2H, OCH_2_-a), 3.78 (ddd, *J* = 9.6, 5.0, 2.6 Hz, 1H, H-5), 2.13–2.11 (m, 3H, OAc), 2.10 (s, 3H, OAc), 2.05 (s, 3H, OAc), 2.04–2.01 (m, 3H, OAc), 1.17–1.02 (m, 3H, CHC*H*_3_, OCH_2_C*H*-b), 1.00–0.92 (m, 1H, C*H*CH_3_-b), 0.89–0.80 (m, 1H, OCH_2_C*H*-a), 0.80–0.66 (m, 1H, CH_2_-b, CHCH_3_-a), 0.52–0.40 (m, 1H, CH_2_-a), 0.36–0.22 (m, 1H, CH_2_-a), 0.05–0.00 (m, 1H, CH_2_-b); ^13^C NMR (101 MHz, CDCl_3_) δ 170.6 (C=O), 170.1 (C=O), 169.6 (C=O), 168.5 (C=O), 156.8 (C=O), 90.7 (C-1), 73.3 C-5), 71.5 (C-3), 69.8 (OCH_2_), 65.3 (C-3), 61.9 (C-6), 51.2 (C-2), 20.78 (OAc), 20.76 (OAc), 20.71 (OAc), 20.68 (OAc), 20.64 (OAc), 18.4 (CH*C*H_3_-a), 18.3 (OCH_2_*C*H-a), 14.3 (OCH_2_*C*H-b), 13.2 (CH*C*H_3_-b), 11.6, 11.5, 11.4 (CH*C*H_2_), 11.0 (CHCH_3_-a), 9.9 (CHCH_3_-b); HRMS *m/z*: [M + Na]^+^ calcd for C_20_H_29_NO_11_, 482.1633; found, 482.1623.

**Ac_4_GlcNCp:** Glucosamine hydrochloride (0.50 g, 2.32 mmol) was suspended under argon atmosphere in dry methanol (40 mL) and sodium methoxide in methanol (0.5 M, 4.7 mL, 2.34 mmol) was added. After 20 min, 2,5-dioxopyrrolidin-1-yl cycloprop-2-ene-1-carboxylate (**6**, 0.63 g, 3.48 mmol) was added and the reaction was stirred at room temperature for 16 h, turning the solution from colorless to yellow. The solvent was removed under reduced pressure and the residue dissolved in pyridine (30 mL) and acetic anhydride (6 mL). After 18 h at room temperature, the solvents were removed under reduced pressure, the brown residue was dissolved in dichloromethane (100 mL) and washed with 10 % aq KHSO_4_ (1 × 75 mL), sat. aq NaHCO_3_ (1 × 75 mL) and brine (1 × 75 mL). The organic layer was dried over MgSO_4_ and the solvent removed under reduced pressure. The crude product was purified by column chromatography (petroleum ether/ethyl acetate 1:2) to yield Ac_4_GlcNCp (183 mg, 19%) as a colorless solid. *R*_f_ = 0.48 (ethyl acetate); α-isomer: ^1^H NMR (400 MHz, CDCl_3_) δ 6.96–6.93 (m, 2H, HC=C*H*), 6.14 (d, *J* = 3.6 Hz, 1H, H-1), 5.48 (d, *J* = 9.1 Hz, 1H, NH), 5.28–5.16 (m, 2H, H-3, H-4), 4.56–4.45 (m, 1H, H-2), 4.25 (dd, *J* = 12.5, 4.2 Hz, 1H, H-6), 4.06 (dd, *J* = 12.5, 2.4 Hz, 1H, H-6), 4.02–3.94 (m, 1H, H-5), 2.18 (s, 3H, OAc), 2.09 (s, 3H, OAc), 2.05–2.03 (m, 7H, OAc, CH); ^13^C NMR (101 MHz, CDCl_3_) δ 175.5 (C=O), 171.8 (C=O), 170.8 (C=O), 169.2 (C=O), 168.7 (C=O), 105.3 (H*C*=CH), 105.1 (HC=*C*H), 91.0 (C-1), 70.9 (C-3), 69.9 (C-5), 67.6 (C-4), 61.7 (C-6), 51.3 (C-2), 21.18 (OAc), 21.02 (OAc), 20.99 (OAc), 20.86 (OAc), 19.0 (*C*H); HRMS *m/z*: [M + Na]^+^calcd for C_18_H_23_NO_10_, 436.1214; found, 436.1212.

**Ac_4_GalNCp:** Galactosamine hydrochloride (0.50 g, 2.32 mmol) was suspended under argon atmosphere in dry methanol (40 mL) and sodium methoxide in methanol (0.5 M, 4.7 mL, 2.34 mmol) was added. After 20 min, 2,5-dioxopyrrolidin-1-yl cycloprop-2-ene-1-carboxylate (**6**, 0.63 g, 3.48 mmol) was added and the reaction was stirred at room temperature for 27 h turning the solution from colorless to yellow. The solvent was removed under reduced pressure and the residue dissolved in pyridine (30 mL) and acetic anhydride (6 mL). After 16 h at room temperature, the solvents were removed under reduced pressure, the brown residue was dissolved in dichloromethane (100 mL), and washed with 10 % aq KHSO_4_ (1 × 100 mL), sat. aq NaHCO_3_ (1 × 100 mL) and brine (1 × 100 mL). The organic layer was dried over MgSO_4_ and the solvent removed under reduced pressure. The crude product was purified by column chromatography (petroleum ether/ethyl acetate 1:2 to pure ethyl acetate) to yield Ac_4_GalNCp (157 mg, 16%) as a colorless solid. *R*_f_ = 0.44 (ethyl acetate); α-isomer: ^1^H NMR (400 MHz, CDCl_3_) δ 6.95 (s, 1H, *H*C=CH), 6.93 (s, 1H, HC=C*H*), 6.18 (d, *J* = 3.6 Hz, 1H, H-1), 5.41 (m, 1H, H-4), 5.38 (d, *J* = 9.3 Hz, 1H, NH), 5.22 (dd, *J* = 11.6, 3.2 Hz, 1H, H-3), 4.75 (ddd, *J* = 11.6, 9.2, 3.7 Hz, 1H, H-2), 4.23 (t, *J* = 6.8 Hz, 1H, H-5), 4.16–4.02 (m, 2H, H-6), 2.16 (s, 6H, 2 x OAc), 2.04 (s, 1H, CH-C=C), 2.03 (s, 3H, OAc), 2.02 (s, 3H, OAc); ^13^C NMR (101 MHz, CDCl_3_) δ 175.9 (C=O), 171.5 (C=O), 170.7 (C=O), 170.5 (C=O), 169.1 (C=O), 105.3 (H*C*=CH), 105.1 (HC=*C*H), 91.7 (C-1), 68.75 (C-5), 68.1 (C-3), 66.9 (C-4), 61.5 (C-6), 47.2 (C-2), 21.2 (OAc), 21.1 (OAc), 20.98 (OAc), 20.95 (OAc), 19.2 (*C*H). HRMS *m*/*z*: [M + Na]^+^ calcd for C_18_H_23_NO_10_, 436.1214; found, 436.1210.

**Ac_4_GlcNCp(H_2_):** Glucosamine hydrochloride (500 mg, 2.32 mmol) was suspended under nitrogen atmosphere in dry *N*,*N*-dimethylformamide (10 mL) and diisopropylethylamine (1.1 mL, 6.33 mmol) was added. After 45 min, 2,5-dioxopyrrolidin-1-yl cyclopropanecarboxylate (**2**, 386 mg, 2.11 mmol) was added and the reaction mixture was stirred at room temperature for 3 days. The solvent was removed under reduced pressure and the residue dissolved in pyridine (4 mL) and acetic anhydride (4 mL). After 1 day at room temperature, the solvents were removed under reduced pressure and coevaporated with ethanol. The brown residue was dissolved in dichloromethane (20 mL) and washed with 10% aq KHSO_4_ (1 × 20 mL), sat. aq NaHCO_3_ (1 × 20 mL) and brine (1 × 20 mL). The organic layer was dried over MgSO_4_ and the solvent removed under reduced pressure. The crude product was purified by column chromatography (petroleum ether/ethyl acetate 1:1) to yield Ac_4_GlcNCp(H_2_) (712 mg, 81%) as a mixture of anomers as a colorless solid. Whereas the α-anomer could be partially separated by column chromatography, semi-preparative RP-HPLC (50–55% B over 20 min) was required to obtain the pure β-anomer (*t*_R_ = 9.5 min). *R*_f_ = 0.41 (petroleum ether/ethyl acetate 1:2); α-isomer: ^1^H NMR (400 MHz, CDCl_3_) δ 6.15 (d, *J* = 3.6 Hz, 1H, H-1), 5.70 (d, *J* = 9.1 Hz, 1H, NH), 5.36–5.07 (m, 2H, H-3, H-4)), 4.49 (ddd, *J* = 10.7, 9.1, 3.7 Hz, 1H, H-2), 4.24 (dd, *J* = 12.4, 4.2 Hz, 1H, H-6), 4.05 (dd, *J* = 12.4, 2.4 Hz, 1H, H-6), 3.99 (ddd, *J* = 9.9, 4.2, 2.4 Hz, 1H, H-5), 2.19 (s, 3H, OAc), 2.07 (s, 3H, OAc), 2.03 (d, *J* = 1.2 Hz, 6H, OAc), 1.33–1.21 (m, 1H, CH), 1.00–0.87 (m, 2H, CH_2_), 0.79–0.68 (m, 2H, CH_2_); ^13^C NMR (101 MHz, CDCl_3_) δ 173.7 (C=O), 171.8 (C=O), 170.8 (C=O), 169.2 (C=O), 168.7 (C=O), 90.9 (C-1), 70.9 (C-3), 69.8 (C-5), 67.6 (C-4), 61.7 (C-6), 51.2 (C-2), 21.0 (OAc), 20.8 (OAc), 20.7 (OAc), 14.6 (CH), 7.8 (CH_2_), 7.7 (CH_2_); β-isomer: ^1^H NMR (400 MHz, CDCl_3_) δ 5.74 (d, *J* = 9.6 Hz, 1H, NH), 5.70 (d, *J* = 8.7 Hz, 1H, H-1), 5.24–5.03 (m, 2H, H-3, H-4), 4.38–4.22 (m, 2H, H-2, H-6), 4.12 (dd, *J* = 12.5, 2.2 Hz, 1H, H-6), 3.89–3.71 (m, 1H, H-5), 2.10 (s, 3H, OAc), 2.08 (s, 3H, OAc), 2.04 (s, 6H, OAc), 1.33–1.22 (m, 1H, CH), 0.98–0.85 (m, 2H, CH_2_), 0.82–0.62 (m, 2H, CH_2_); ^13^C NMR (101 MHz, CDCl_3_) δ 173.9 (C=O), 171.4 (C=O), 170.8 (C=O), 169.7 (C=O), 169.4 (C=O), 92.9 (C-1), 73.2 (C-5), 72.8 (C-3), 68.0 (C-4), 61.9 (C-6), 53.2 (C-2), 21.0 (OAc), 20.9 (OAc), 20.8 (OAc), 20.7 (OAc), 14.8 (CH), 7.64 (CH_2_), 7.58 (CH_2_); HRMS *m/z*: [M + Na]^+^ calcd for C_18_H_25_NO_10_, 438.1371; found, 438.1366.

**Ac_4_GlcNCyoc(H_2_)**: Glucosamine hydrochloride (500 mg, 2.32 mmol) was suspended under nitrogen atmosphere in dry *N*,*N*-dimethylformamide (10 mL) and diisopropylethylamine (1.1 mL, 6.33 mmol) was added. After 45 min, the activated cyclopropane **5** (530 mg, 2.11 mmol) was added and the reaction mixture was stirred at room temperature for 4 days. The solvent was removed under reduced pressure and the residue dissolved in pyridine (2 mL) and acetic anhydride (2 mL). After 3 days at room temperature, the solvents were removed under reduced pressure and coevaporated with ethanol. The brown residue was dissolved in dichloromethane (20 mL) and washed with 10% aq KHSO_4_ (1 × 20 mL), sat. aq NaHCO_3_ (1 × 20 mL) and brine (1 × 20 mL). The organic layer was dried over MgSO_4_ and the solvent removed under reduced pressure. The crude product was purified by column chromatography (petroleum ether/ethyl acetate 2:1) to yield Ac_4_GlcNCyoc(H_2_) (771 mg, 80%) as a colorless solid. Anomers were separated by RP-HPLC (60–70% B over 20 min) and obtained as mixture of isomers. Retention time β-anomer: 12.6 min, α-anomer: 13.4 min. *R*_f_ = 0.30 (petroleum ether/ethyl acetate 3:2); α-isomer: ^1^H NMR (400 MHz, CDCl_3_) δ 6.19 (d, *J* = 3.7 Hz, 1H, H-1), 5.36–5.12 (m, 2H, H-3, H-4), 4.77 (d, *J* = 9.5 Hz, 1H, NH), 4.26 (dd, *J* = 12.5, 4.1 Hz, 1H, H-6), 4.23–4.13 (m, 1H, H-2), 4.05 (dd, *J* = 12.6, 2.2 Hz, 1H, H-6), 4.02–3.95 (m, 1H, H-5), 3.96–3.73 (m, 2H, OCH_2_), 2.18 (s, 3H, OAc), 2.08 (s, 3H, OAc), 2.05 (s, 3H, OAc), 2.03 (s, 3H, OAc), 1.03 (d, *J* = 6.0 Hz, 3H, CHC*H*_3_), 0.83–0.73 (m, 1H, OCH_2_C*H*), 0.73–0.59 (m, 1H, C*H*CH_3_), 0.53–0.34 (m, 1H, CH_2_), 0.34–0.23 (m, 1H, CH_2_); ^13^C NMR (101 MHz, CDCl_3_) δ 171.2 (C=O), 170.6 (C=O), 169.2 (C=O), 168.6 (C=O), 155.9 (C=O), 90.9 (C-1), 70.7 (C-3), 69.9 (OCH_2_), 69.7 (C-5), 67.7 (C-4), 61.6 (C-6), 52.7 (C-2), 20.9 (OAc), 20.7 (OAc), 20.5 (OAc), 18.4 (CH*C*H_3_), 18.3 (OCH_2_*C*H), 11.6 (CH_2_), 11.4 (*C*HCH_3_); β-isomer: ^1^H NMR (600 MHz, CDCl_3_) δ 5.70 (d, *J* = 8.7 Hz, 1H, H-1), 5.18 (‘t‘, *J* = 9.9 Hz, 1H, H-3), 5.11 (‘t‘, *J* = 9.6 Hz, 1H, H-4), 4.76–4.61 (m, 1H, NH), 4.29 (dd, *J* = 12.5, 4.6 Hz, 1H, H-6), 4.11 (dd, *J* = 12.5, 2.3 Hz, 1H, H-6), 3.97–3.84 (m, 3H, H-2, OCH_2_), 3.84–3.76 (m, 1H, H-5), 2.12 (s, 3H, OAc), 2.09 (s, 3H, OAc), 2.05 (s, 3H, OAc), 2.03 (s, 3H, OAc), 1.03 (d, *J* = 6.0 Hz, 3H, CH_3_), 0.84–0.75 (m, 1H, OCH_2_C*H*), 0.72–.64 (m, 1H, C*H*CH_3_), 0.49–0.37 (m, 1H, CH_2_), 0.31–0.21 (m, 1H, CH_2_); ^13^C NMR (151 MHz, CDCl_3_) δ 170.8 (C=O), 169.5 (C=O), 169.5 (C=O), 156.2 (C=O), 92.8 (C-1), 92.7 (C-1), 73.0 (C-5), 72.5 (C-3), 72.4 (C-3), 69.9 (OCH_2_), 68.1 (C-4), 61.8 (C-6), 55.0 (C-2), 21.0 (OAc), 20.9 (OAc), 20.8 (OAc), 20.7 (OAc), 18.6 (CH*C*H_3_), 18.5 (OCH_2_*C*H), 11.71, 11.68, 11.6 (CH_2_, *C*HCH_3_); HRMS *m/z*: [M + Na]^+^ calcd for C_20_H_29_NO_11_, 482.1633; found, 482.1624.

**ManNCp(H_2_)**: Ac_4_ManNCp(H_2_) (80 mg, 0.19 mmol) was dissolved under nitrogen atmosphere in dry methanol (5 mL) and sodium methoxide (0.5 M, 0.06 mL) was added. After stirring overnight, Amberlite IR 120 was added for neutralization. The resin was filtered off and the solvent was removed under reduced pressure to obtain ManNCp(H_2_) as slightly yellow solid (40 mg, 84%) which was used without further purification for the aldolase reaction.

**ManNCyc(H_2_):** Ac_4_ManNCyc(H_2_) (67 mg, 0.16 mmol) was dissolved under nitrogen atmosphere in dry methanol (4.5 mL) and sodium methoxide (0.5 M, 0.05 mL) was added. After stirring overnight, Amberlite IR 120 was added for neutralization. The resin was filtered off and the solvent was removed under reduced pressure to obtain ManNCyc(H_2_) as slightly colorless solid (37 mg, 88%) which was used without further purification for the aldolase reaction.

**ManNCyoc(H_2_):** Ac_4_ManNCyoc(H_2_) (70.9 mg, 0.16 mmol) was dissolved in methanol (3.2 mL) and *N*,*N*-dimethylethylamine (0.7 mL, 6.82 mmol) was added. After stirring for eight days, the solvents were removed under reduced pressure and ManNCyoc(H_2_) was obtained as colorless solid (45 mg, quant.) which was used without further purification for the aldolase reaction.

**Sialic acid aldolase reaction:** In a polypropylene vial, the sugar derivatives ManNCp(H_2_), ManNCyc(H_2_) and ManNCyoc(H_2_), respectively, were dissolved in phosphate buffer (100 mM, pH 7.16) to a final concentration of 0.1 M. Sodium pyruvate (15 equiv.) and sialic acid aldolase (a spatula tip) were added. After stirring for 17 days the mixture was concentrated under reduced pressure, diluted with ethanol and filtered through cotton. The solvents were removed under reduced pressure and the crude product purified via RP-HPLC.

**Neu5Cp(H_2_):** RP-HPLC (5–10% over 20 min): *t*_R_ = 9.1 min, HRMS *m/z*: [M − H]^−^ calcd for C_13_H_21_NO_9_, 334.1144; found, 334.1219.

**Neu5Cyc(H_2_):** RP-HPLC (5–10% over 20 min): *t*_R_ = 15.0 min, HRMS *m/z*: [M − H]^−^ calcd for C_14_H_23_NO_9_, 348.1300; found, 348.1381.

**Neu5Cyoc(H_2_):** RP-HPLC (5–20% over 20 min): *t*_R_ = 13.4 min, HRMS *m/z*: [M − H]^−^ calcd for C_15_H_25_NO_10_, 378.1405; found, 378.1492.

**Preparation of DMB labeling solution:** The stock solution for DMB labeling was prepared with Na_2_S_2_O_4_ (18 mM), 2-mercaptoethanol (1 M) and TFA (40 mM) in Milli-Q water and was stored at 8 °C. 1,2-Diamino-4,5-methylenedioxybenzene dihydrochloride (DMB·2HCl) was added on the day of the experiment to a final concentration of 5.3 mM.

**DMB labeling of reference compounds:** The sialic acid derivatives Neu5Cp(H_2_), Neu5Cyc(H_2_), Neu5Cyoc(H_2_) (0.1–0.2 mg), respectively, were dissolved in DMB labeling solution (265 μL) and incubated for 2.5 h at 56 °C in a thermomixer (300 rpm). The mixture was cooled on ice for 10 minutes and neutralized with sodium hydroxide (0.5 M, 25 μL). The solutions were analyzed via RP-HPLC-MS. For fluorescence detection (λ_ex_ = 372 nm, λ_em_ = 456 nm), they were diluted with Milli-Q water (1:400).

To determine their retention times, the literature known compounds DMB-Neu5Ac and DMB-Sodium pyruvate were synthesized following the above-mentioned protocol as well.

**DMB-Neu5Cp(H_2_):** Analytical RP-HPLC (10–25% over 40 min): *t*_R_ = 17.2 min, MS *m/z*: [M + H]^+^ calcd for C_20_H_25_N_3_O_9_, 452.17; found, 452.10.

**DMB-Neu5Cyc(H_2_):** Analytical RP-HPLC (10–25% over 40 min): *t*_R_ = 24.0; 24,4, MS *m/z*: [M + H]^+^ calcd for C_21_H_27_N_3_O_9_, 466.18; found, 466.15.

**DMB-Neu5Cyoc(H_2_):** Analytical RP-HPLC (10–40% over 40 min): *t*_R_ = 24.9; 25.2; 25.7, MS *m/z*: [M + H]^+^ calcd for C_22_H_29_N_3_O_10_, 496.19; found, 496.20.

**Kinetic measurements:** For kinetic studies, ManNCyc [23] and ManNCp [27] were synthesized according to the literature excluding the peracetylation step. Stock solutions of Tz-PEG-OH and sugar were prepared in acetate buffer (pH 4.8) and mixed in a quartz cuvette to give final concentrations of 1 mM Tz-PEG-OH and 10 mM, 13.3 mM and 16.6 mM, respectively, of ManCyc or ManCp. The reaction was monitored by measuring the absorption of the tetrazine at 522 nm. Pseudo-first-order rate constants were determined for every concentration of ManNCyc and ManNCp, respectively, by plotting ln(*A*_0_/*A*_t_) versus time. For the determination of *A*_0,_ a 1 mM solution of Tz-PEG-OH was used. *A*_t_ is the absorption of the reaction at time point *t*. Analysis by linear regression provided pseudo-first-order rate constants. Second-order-rate constants were determined by plotting the pseudo-first-order rate constants versus the corresponding sugar concentration, followed by linear regression and determination of the slope. All measurements were carried out in triplicate.

**Cell growth conditions:** HEK 293T (human embryonic kidney) cells were grown in Dulbecco’s Modified Eagle’s Medium (DMEM) containing fetal bovine serum (FBS, 10%) and penicillin and streptomycin (each 100 U mL^−1^). Cells were incubated under carbon dioxide (5%) in a water-saturated incubator at 37 °C. The cells were diluted every 3 to 4 days by washing with PBS buffer and detaching with trypsin and EDTA.

**Sugar stock solutions:** The sugars were prepared as stock solutions (100 mM) in DMSO and stored at −20 °C. They were freshly diluted into media on the day of the experiment.

**Fluorescence microscopy:** In an approach similar to that used in previously described experiments [24], HEK 293T cells (18000 cells cm^−1^) were seeded in a 4-well ibiTreat µ-Slides (ibidi) Ph+ coated with poly-L-lysine (0.0025%, 1 h at 37 °C or overnight at 4 °C) and allowed to attach for 20 h. Cells were then incubated with Ac_4_ManNCyc (100 μM), Ac_4_ManNCp (100 μM), Ac_4_ManNCyoc (100 μM), Ac_4_GlcNCyoc (50 μM), Ac_4_GlcNCp (100 μM or 50 μM), and Ac_4_GalNCp (100 μM) for 48 h. DMSO only was added as solvent control. Cells were washed twice with PBS and then treated with Tz-biotin (100 μM or 500 μM) for 1–3 h at 37 °C. After two washes with PBS, cells were incubated with streptavidin-AlexaFluor 555 (6.6 μL mL^−1^) and Hoechst 33342 (10 μg mL^−1^) for 20 min at 37 °C in the dark. Cells were washed thrice with PBS and DMEM was added for microscopy. Confocal fluorescence microscopy was performed with a Zeiss LSM 880 instrument equipped with a 40×1.4 NA Plan-Apochromat oil immersion objective and a GaAsP-detector array for spectral imaging. The obtained data were analyzed with image J software version 1.51.

**Western blot analysis:** Western Blot analysis was performed by a modified version of the previously described protocol [26,32]. HEK 293T cells were seeded (800000 cells/10 cm dish), and allowed to attach for 20 h. Cells were then incubated with Ac_4_ManNCp (100 μM), Ac_4_GalNCp (100 μM), Ac_4_GlcNCp (100 μM), and Ac_4_GlcNCyoc (100 μM) for 48 h. DMSO only was added as solvent control. Cells were trypsinated, resuspended in PBS (10 mL), and pelleted by centrifugation (5 min, 400*g*). The supernatant was discarded, and the pellet was resuspended in PBS (1 mL) and pelleted by centrifugation (5 min, 400*g*). The cells were lysed in lysis buffer (180 μL) containing Triton X-100 (0.5%), DNase (30 μg mL^−1^), RNase (30 μg mL^−1^), β-glycerophophate (20 mM), sodium fluoride (20 mM), sodium orthovanadate (0.3 mM), complete X protease inhibitor (Roche; 1×), NaCl (300 mM), Tris·HCl (pH 7.4, 25 mM), EDTA (5 mM), and 2-acetamido-2-deoxy-D-glucopyranosylidenamino *N*-phenylcarbamate [PUGNAc (*O*-GlcNAc-β-*N*-acetylglucosaminidase inhibitor to maintain *O*-GlcNAcylation during lysis), Sigma-Aldrich, 100 μM], and incubation was carried out at 4 °C for 30 min. The lysate was cleared by centrifugation (20000*g*, 30 min, 4 °C). Tz-Cy3 (3-(*p*-Benzylamino)-1,2,4,5-tetrazine-Cy3, Jena Bioscience) was added to the sample to afford a final concentration of 10 μM. The samples were incubated for 90 min at 24 °C, SDS-sample buffer (4×) was added and the sample was heated at 95 °C for 10 min. Proteins were separated by SDS-polyacrylamide gel electrophoresis with 10% polyacrylamide gels and transferred to nitrocellulose membranes (BioRad). Transfer efficiency and equal loading was analyzed by Ponceau S staining. The Cy3 fluorescence was detected with an Amersham Imager 600 using a 520 nm long pass filter.

**Flow cytometry analysis:** For flow cytometry analysis, the previously described protocol [21] was modified. HEK 293T cells were seeded in 12-well plates (150000 cells/well) coated with poly-L-lysine (0.0025%, 1 h at 37 °C or overnight at 4 °C). After 20 h cells were incubated with Ac_4_ManNCyc (100 μM), Ac_4_ManNCp (100 μM), Ac_4_ManNCyoc (100 μM), Ac_4_GlcNCyoc (50 μM), Ac_4_GlcNCp (100 μM or 50 μM), or Ac_4_GalNCp (100 μM) for 48 h. DMSO only was added as solvent control. Cells were washed twice with PBS and then treated with Tz-biotin (100 or 500 μM) for 30 min or 1 h at 37 °C. After two washes with PBS, cells were incubated with streptavidin-Alexa Fluor 555 (6.6 μL mL^−1^) for 20 min at 37 °C in the dark. Cells were washed twice with PBS, released with trypsin-EDTA (200 μL/well), and resuspended in flow cytometry staining buffer (thermo fisher scientific) (600 μL/well). 10000 cells were counted per measurement. For flow cytometry analysis, BD LSRFortessa was used and the obtained data were evaluated with FlowJo Software version 8.8.7. Experiments were performed in triplicate.

**DMB labeling of sialic acids released from engineered cells:** In an approach similar to that described previously [20], HEK 293T cells were seeded in 6 cm dishes (400000 cells/dish). After 20 h cells were incubated with Ac_4_ManNCp(H_2_) (100 μM), Ac_4_ManNCyc(H_2_) (100 μM), Ac_4_ManNCyoc(H_2_) (100 μM), Ac_4_GlcNCp(H_2_) (100 μM), or Ac_4_GlcNCyoc(H_2_) (100 μM). DMSO was added as solvent control. After 2 days, the media, except for 1 mL, was discarded. The cells were harvested in the leftover media, transferred to an Eppendorf tube and pelleted by centrifugation (5 min, 500*g*). The supernatant was discarded and the pellet was washed twice by resuspension in PBS (800 μL) and centrifugation (5 min, 500*g*). Cells were resuspended in PBS (1 mL), counted and transferred in a new Eppendorf tube (400000 cells/tube). The cells were pelleted again and the supernatant discarded. The pellet was resuspended in AcOH (3 M, 300 μL) and incubated for 90 min at 80 °C. The mixture was diluted with Milli-Q water and neutralized with aq. NH_3_ (25%, 20 μL). The solvents were removed under reduced pressure using a SpeedVac and the residue was coevaporated with ethanol (3×) to obtain a colorless solid. At this point, the samples could be stored for a few days at −20 °C. For DMB labeling, the pellets were dissolved in DMB labeling solution (265 μL) and incubated for 2.5 h at 56 °C in a thermomixer (300 rpm). The mixture was cooled on ice for 10 min and neutralized with sodium hydroxide (0.5 M, 25 μL). Analysis was performed by analytical RP-HPLC using a fluorescence detector (λ_ex_ = 372 nm, λ_em_ = 456 nm).

## Supporting Information

File 1 Additional figures and ^1^H and ^13^C NMR spectra of new compounds.
