# Supplementary material for: Cyclopropene derivatives of aminosugars for metabolic glycoengineering
Source: Beilstein J Org Chem. 2019 Mar 4;15:584–601. doi: 10.3762/bjoc.15.54 (PMC6423581; doi:10.3762/bjoc.15.54)
Supplement: File 1 — Additional figures and 1H and 13C NMR spectra of new compounds. [file Beilstein_J_Org_Chem-15-584-s001.pdf]

BEILSTEIN JOURNAL OF ORGANIC CHEMISTRY

Supporting Information

for

Cyclopropene derivatives of aminosugars for metabolic glycoengineering

Jessica Hassenrück and Valentin Wittmann

Beilstein J. Org. Chem. **2019,** *15,* 584–601. doi:10.3762/bjoc.15.54

Additional figures and ^{1}H and ^{13}C NMR spectra of new compounds

License and Terms: This is a supporting information file under the terms of the Creative Commons Attribution License (http://creativecommons.org/licenses/by/4.0). Please note that the reuse, redistribution and reproduction in particular requires that the authors and source are credited. The license is subject to the *Beilstein Journal of Organic Chemistry* terms and conditions: (https://www.beilstein-journals.org/bjoc)

Contents

Additional confocal fluorescence microscopy images.. S2

RP-HPLC analysis of DMB-labeled sialic acids.. S3

RP-HPLC analysis of DMB-labeled sialic acids released from engineered cells.. S7

NMR spectra... S13

Additional confocal fluorescence microscopy images

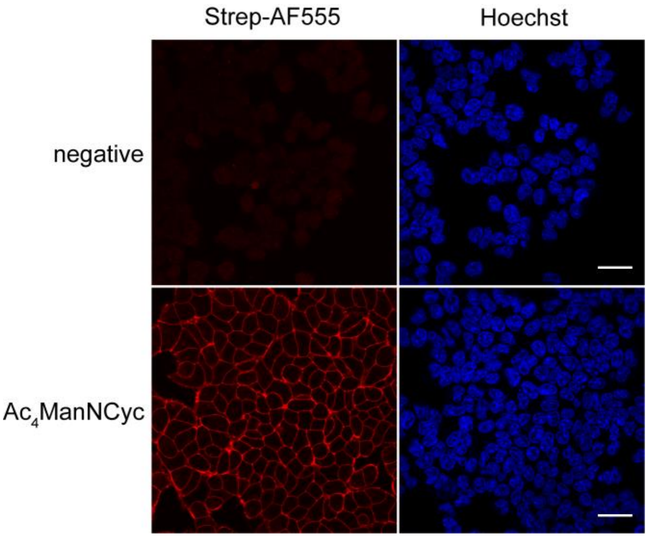

Figure S1: HEK 293T cells were grown with 100 μM Ac_4ManNCyc or DMSO only (negative control) for 48 h. Cells were incubated with Tz-biotin (100 μM) for 1 h at 37 °C followed by incubation with streptavidin-AlexaFluor 555. Nuclei were stained with Hoechst 33342. Scale bar: 30 μm.

RP-HPLC analysis of DMB-labeled sialic acids

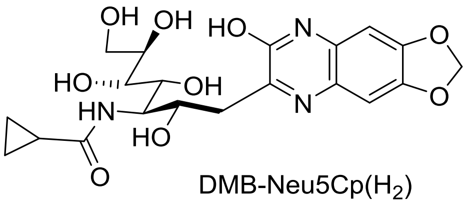

DMB-Neu5Cp(H_2)

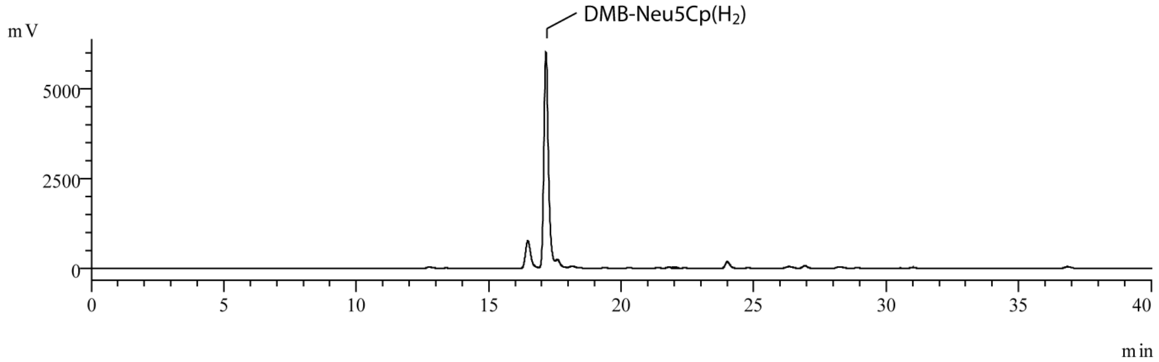

Figure S2: Analysis of DMB-Neu5Cp(H_2) by RP-HPLC (10–25% B in 40 min) with a fluorescence detector (λ_{ex} = 372 nm, λ_{em} = 456 nm). t_R = 17.2 min.

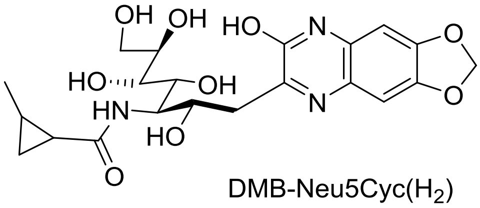

DMB-Neu5Cyc(H_2)

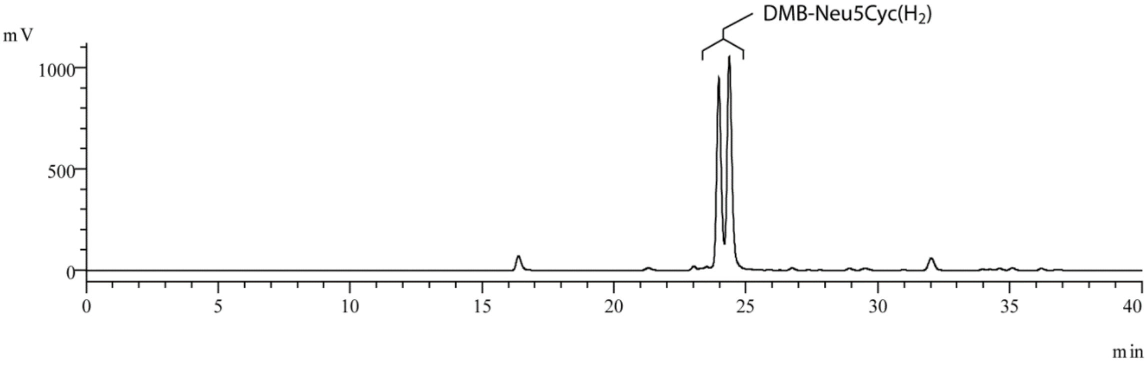

Figure S3: Analysis of DMB-Neu5Cyc(H_2) by RP-HPLC (10–25% B in 40 min) with a fluorescence detector (λ_{ex} = 372 nm, λ_{em} = 456 nm). t_R = 24.0; 24,4 min.

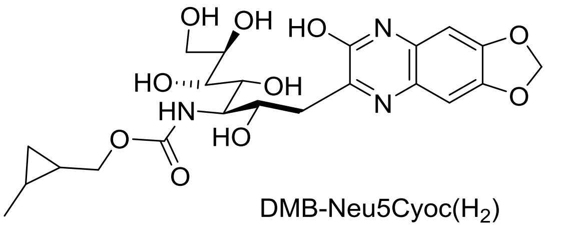

DMB-Neu5Cyoc(H_2)

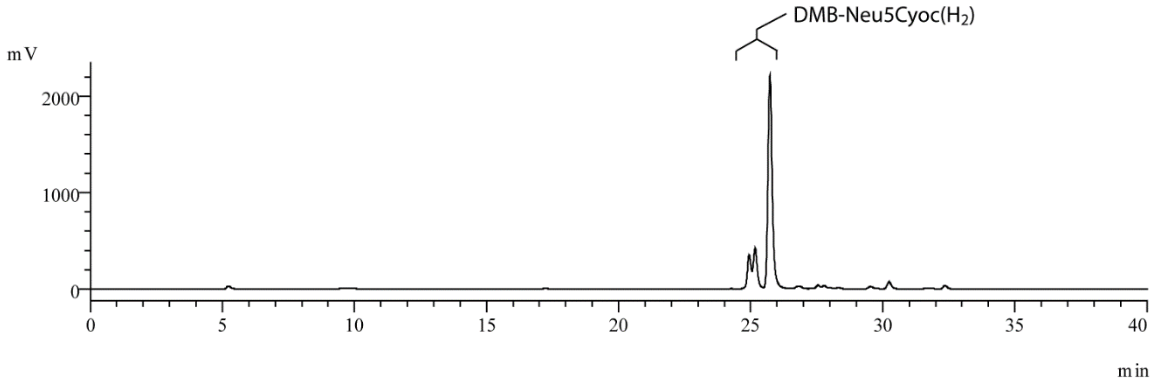

Figure S4: Analysis of DMB-Neu5Cyoc(H_2) by RP-HPLC (10–40% B in 40 min) with a fluorescence detector (λ_{ex} = 372 nm, λ_{em} = 456 nm). t_R = 24.9; 25.2; 25.7 min.

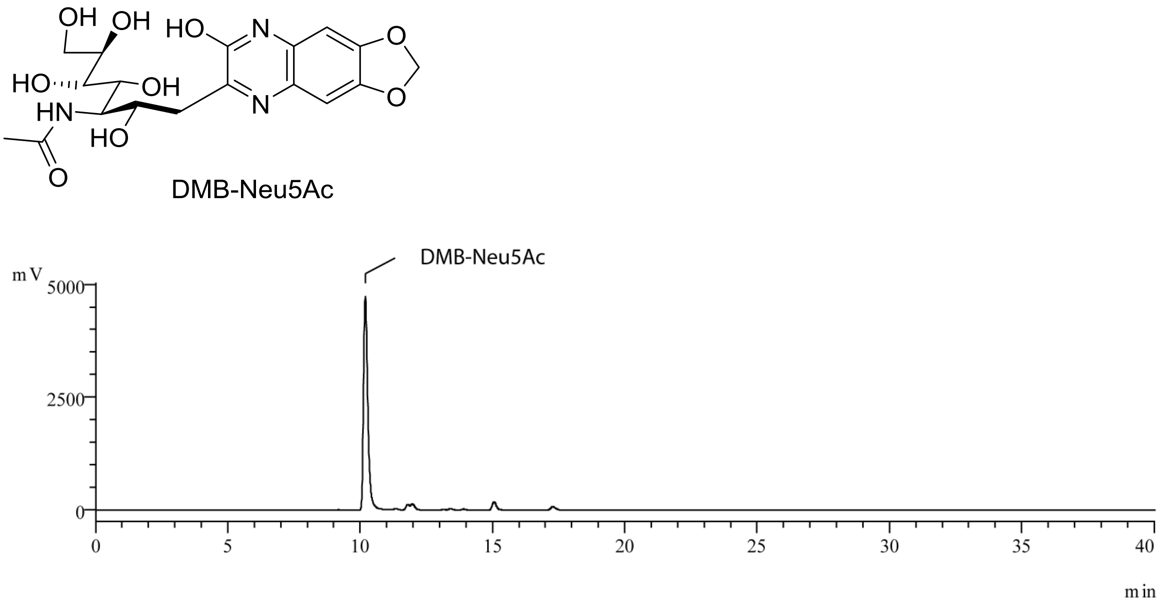

Figure S5: Analysis of DMB-Neu5Ac by RP-HPLC (10–25% B in 40 min) with a fluorescence detector (λ_{ex} = 372 nm, λ_{em} = 456 nm). t_R = 10.2 min.

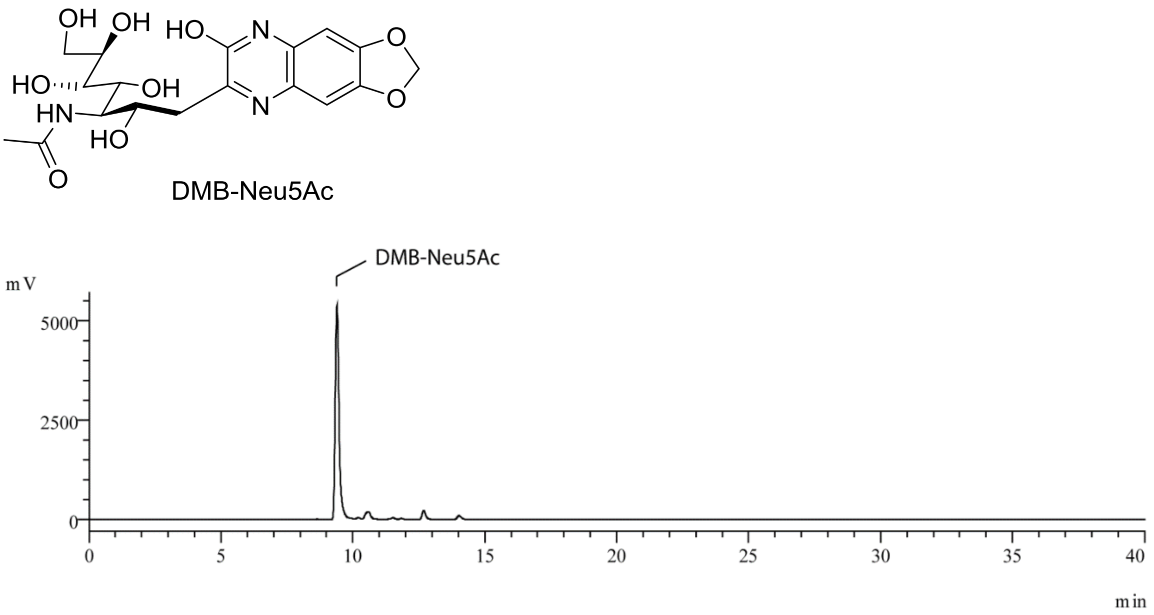

Figure S6: Analysis of DMB-Neu5Ac by RP-HPLC (10–40% B in 40 min) with a fluorescence detector (λ_{ex} = 372 nm, λ_{em} = 456 nm). t_R = 9.4 min.

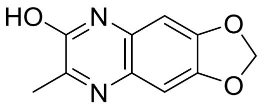

DMB-Pyruvate

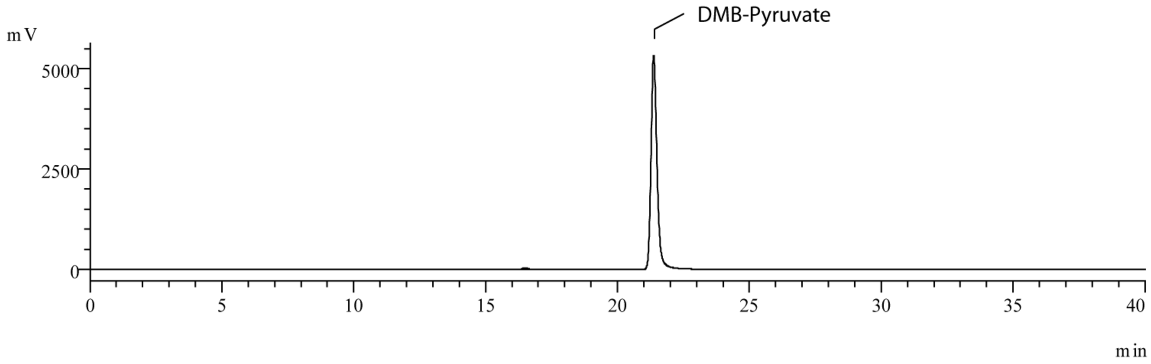

Figure S7: Analysis of DMB-Pyruvate by RP-HPLC (10–25% B in 40 min) with a fluorescence detector (λ_{ex} = 372 nm, λ_{em} = 456 nm). t_R = 21.4 min.

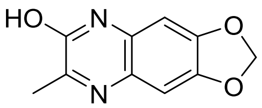

DMB-Pyruvate

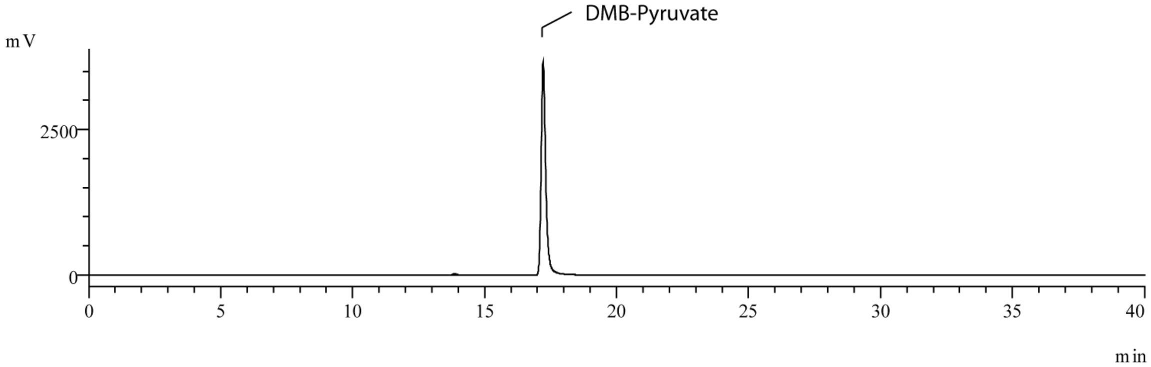

Figure S8: Analysis of DMB-Pyruvat by RP-HPLC (10–40% B in 40 min) with a fluorescence detector (λ_{ex} = 372 nm, λ_{em} = 456 nm). t_R = 17.2 min.

RP-HPLC analysis of DMB-labeled sialic acids released from engineered cells

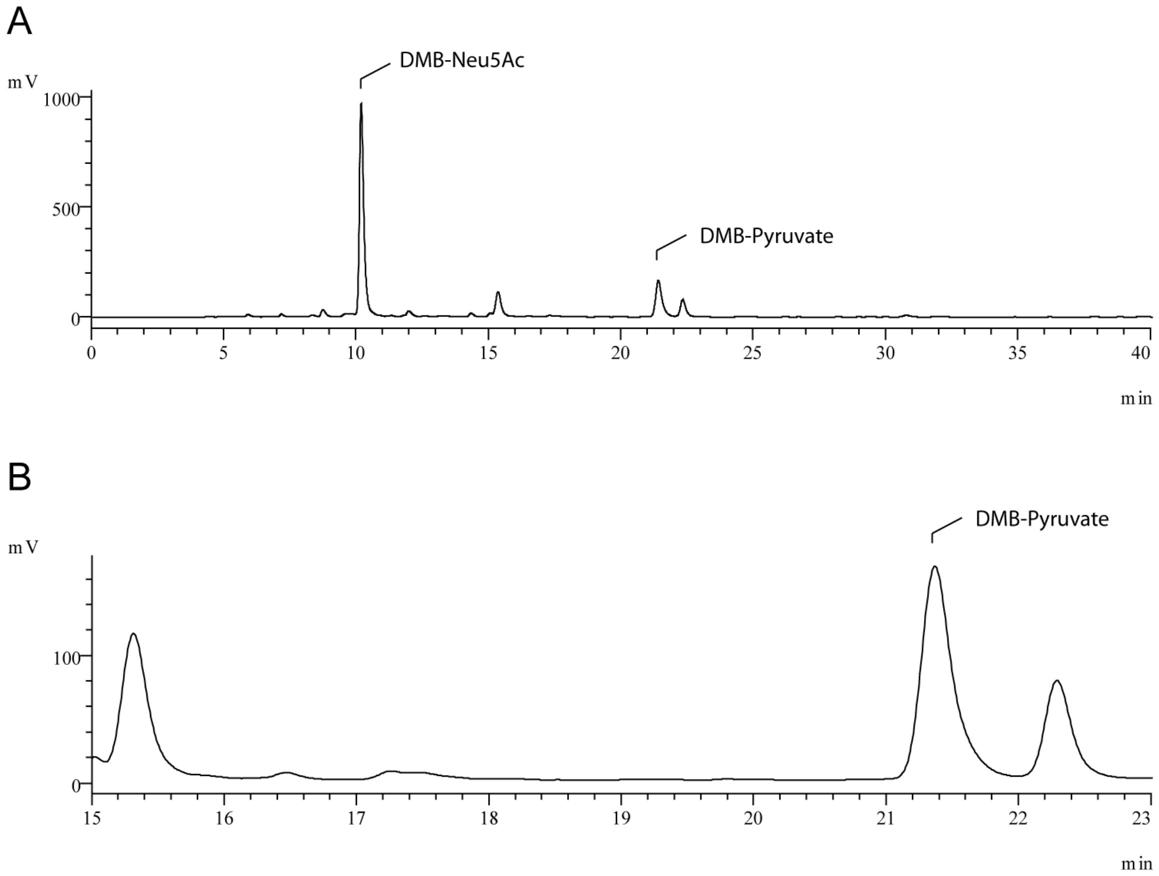

Figure S9: Analysis of DMB-labeled sialic acids released from cells grown without additional sugar (DMSO only, solvent control) by RP-HPLC (10–25% B in 40 min) with a fluorescence detector (λ_{ex} = 372 nm, λ_{em} = 456 nm). A) Complete chromatogram. B) Enlarged region.

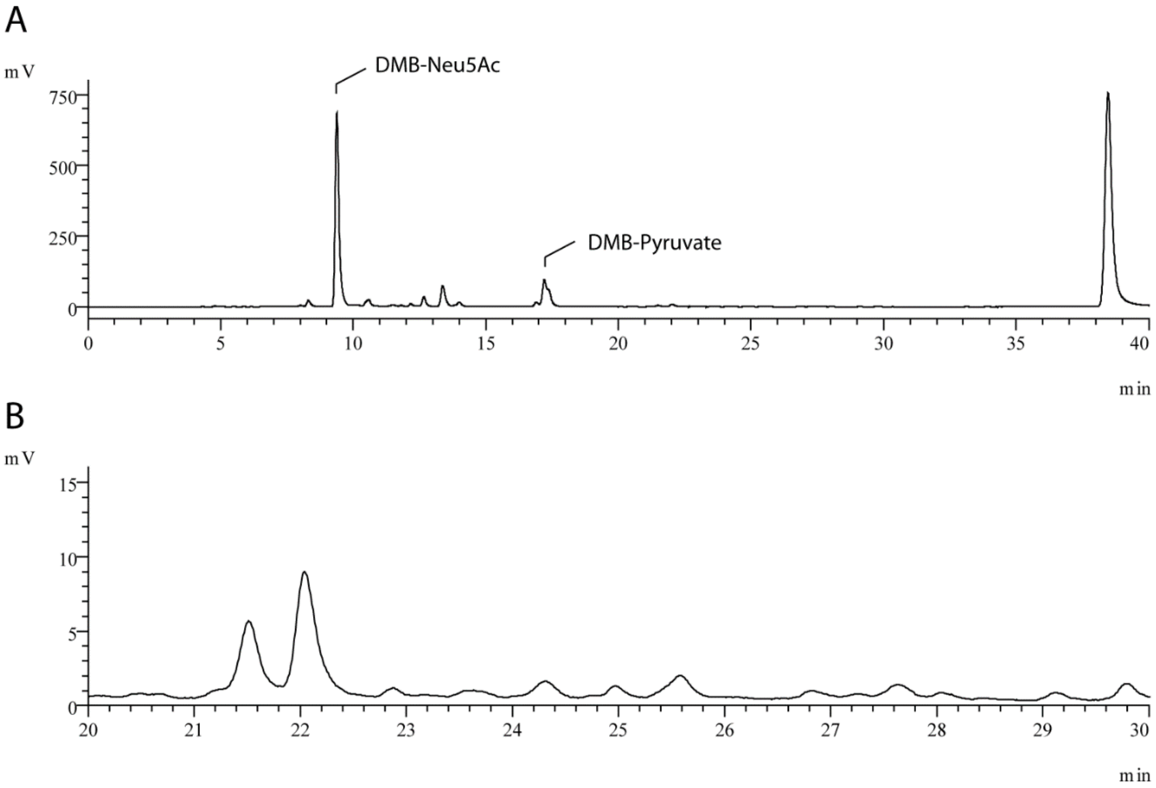

Figure S10: Analysis of DMB-labeled sialic acids released from cells grown without additional sugar (DMSO only, solvent control) by RP-HPLC (10–40% B in 40 min) with a fluorescence detector (λ_{ex} = 372 nm, λ_{em} = 456 nm). A) Complete chromatogram. B) Enlarged region.

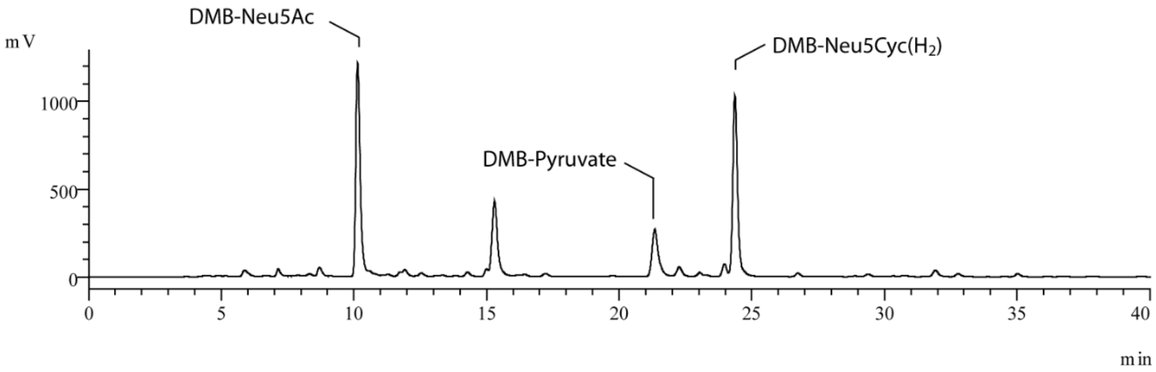

Figure S11: Analysis of DMB-labeled sialic acids released from cells grown with Ac_4ManNCyc(H_2) by RP-HPLC (10–25% B in 40 min) with a fluorescence detector (λ_{ex} = 372 nm, λ_{em} = 456 nm).

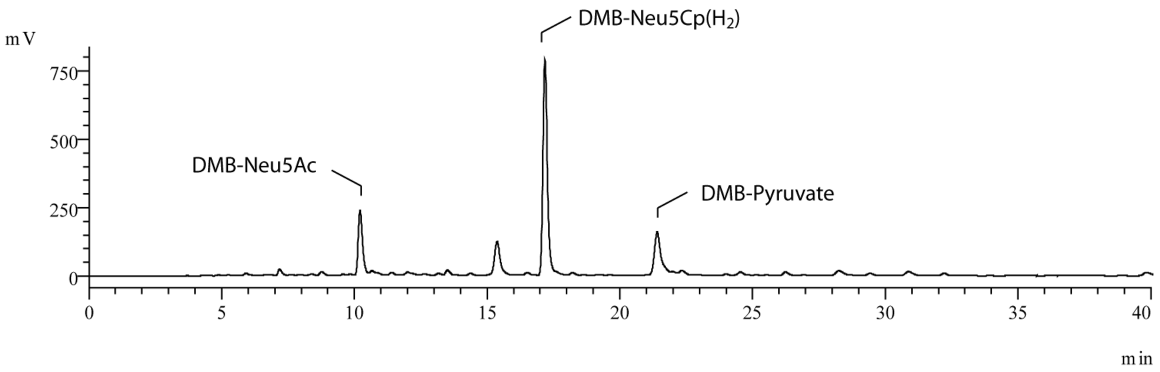

Figure S12: Analysis of DMB-labeled sialic acids released from cells grown with Ac_4ManNCp(H_2) by RP-HPLC (10–25% B in 40 min) with a fluorescence detector (λ_{ex} = 372 nm, λ_{em} = 456 nm).

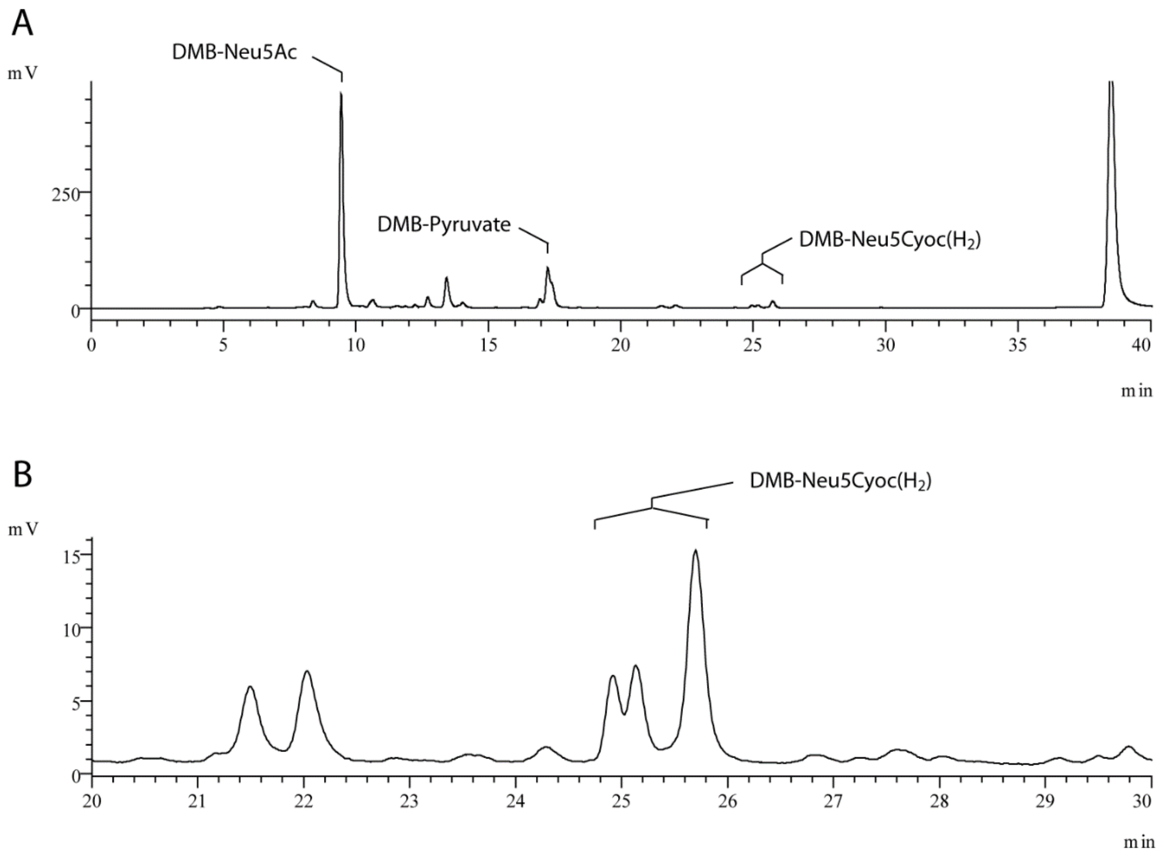

Figure S13: Analysis of DMB-labeled sialic acids released from cells grown with Ac_4ManNCyoc(H_2) by RP-HPLC (10–40% B in 40 min) with a fluorescence detector (λ_{ex} = 372 nm, λ_{em} = 456 nm). A) Complete chromatogram. B) Enlarged region.

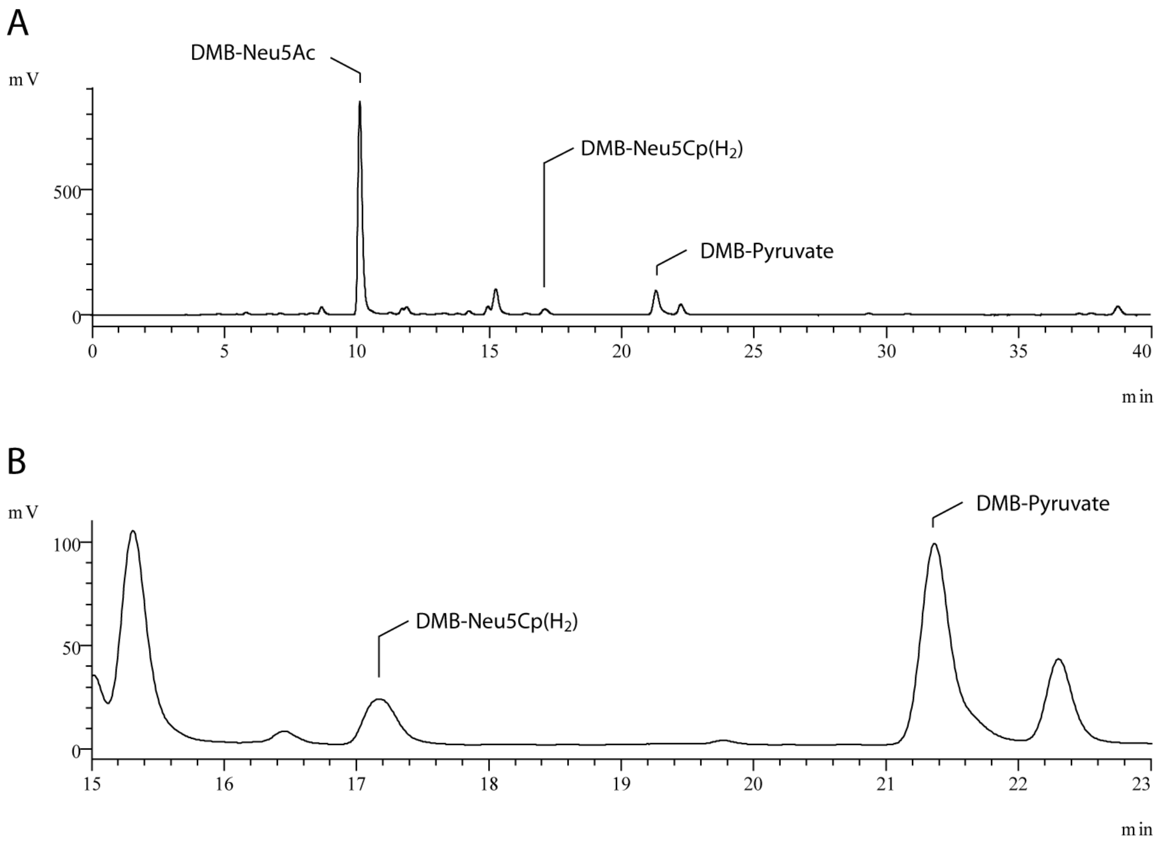

Figure S14: Analysis of DMB-labeled sialic acids released from cells grown with $Ac_4GlcNCp(H_2)$ by RP-HPLC (10–25% B in 40 min) with a fluorescence detector (λ_{ex} = 372 nm, λ_{em} = 456 nm). A) Complete chromatogram. B) Enlarged region.

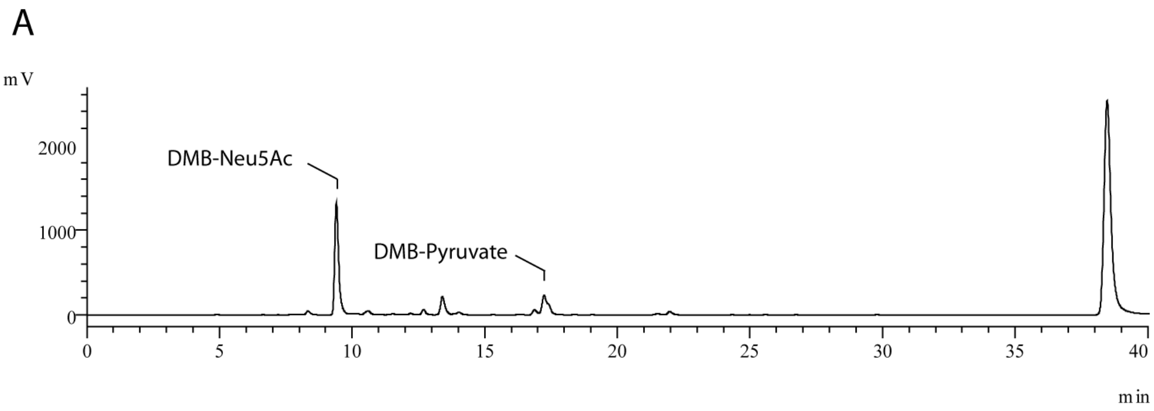

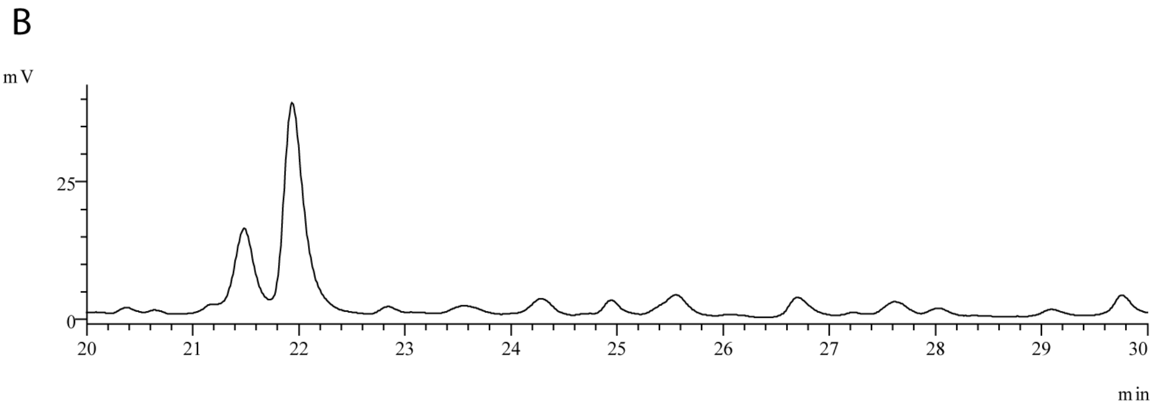

Figure S15: Analysis of DMB-labeled sialic acids released from cells grown with $Ac_4GlcNCyoc(H_2)$ by RP-HPLC (10–40% B in 40 min) with a fluorescence detector (λ_{ex} = 372 nm, λ_{em} = 456 nm). A) Complete chromatogram. B) Enlarged region.

NMR spectra

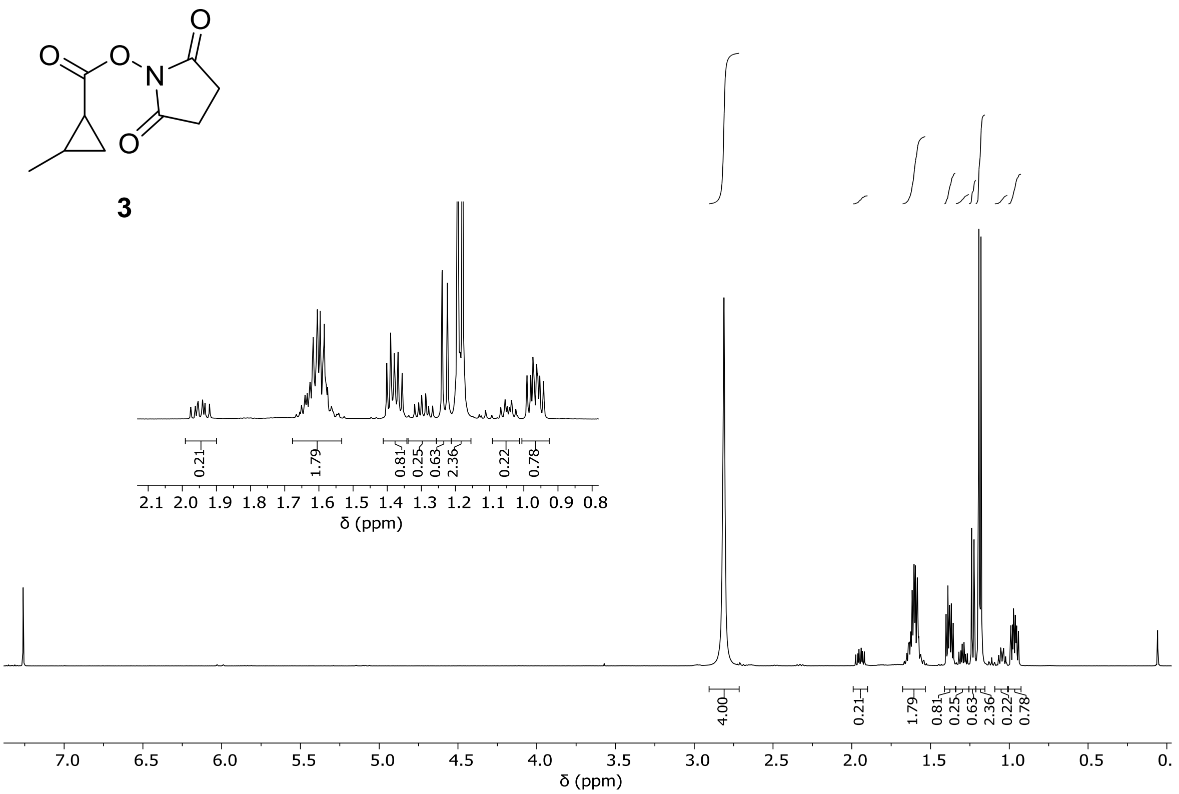

^{1}H NMR spectrum ($CDCl_3$, 400 MHz) of **3**.

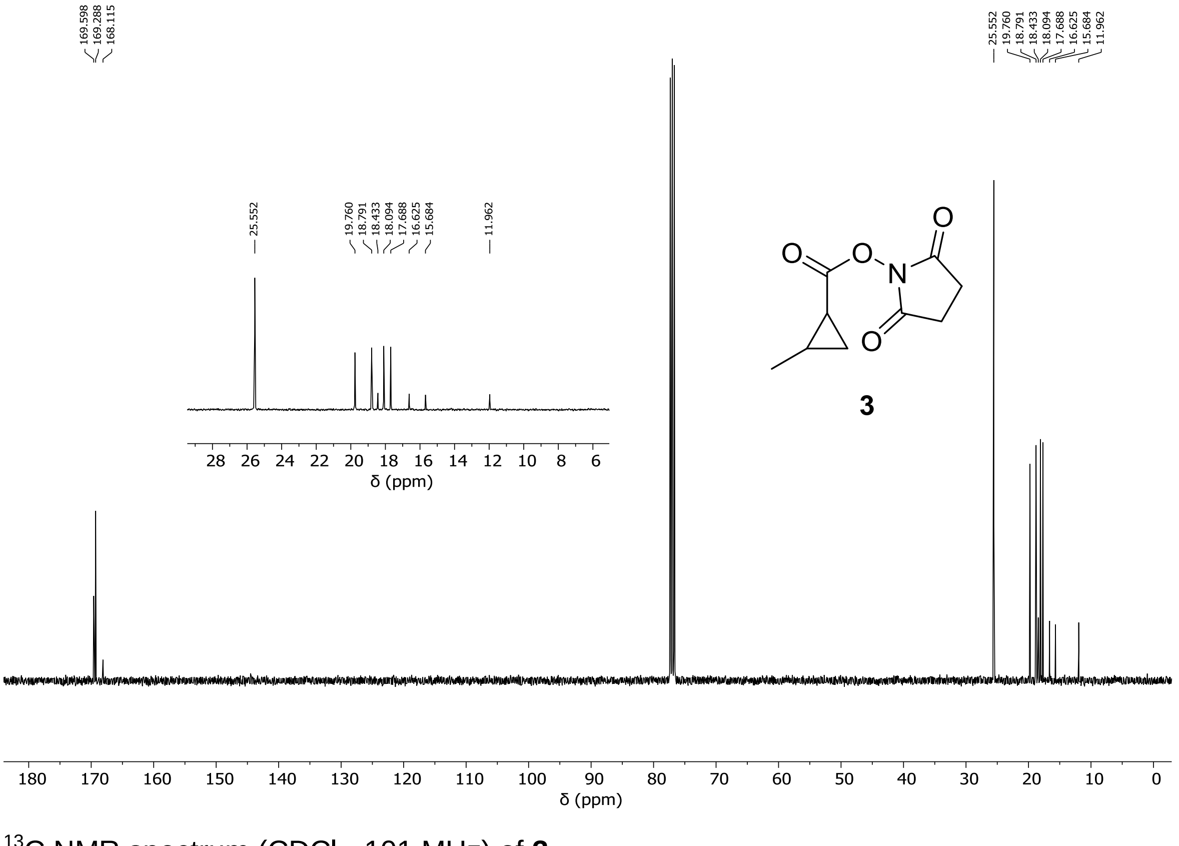

^{13}C NMR spectrum ($CDCl_3$, 101 MHz) of **3**.

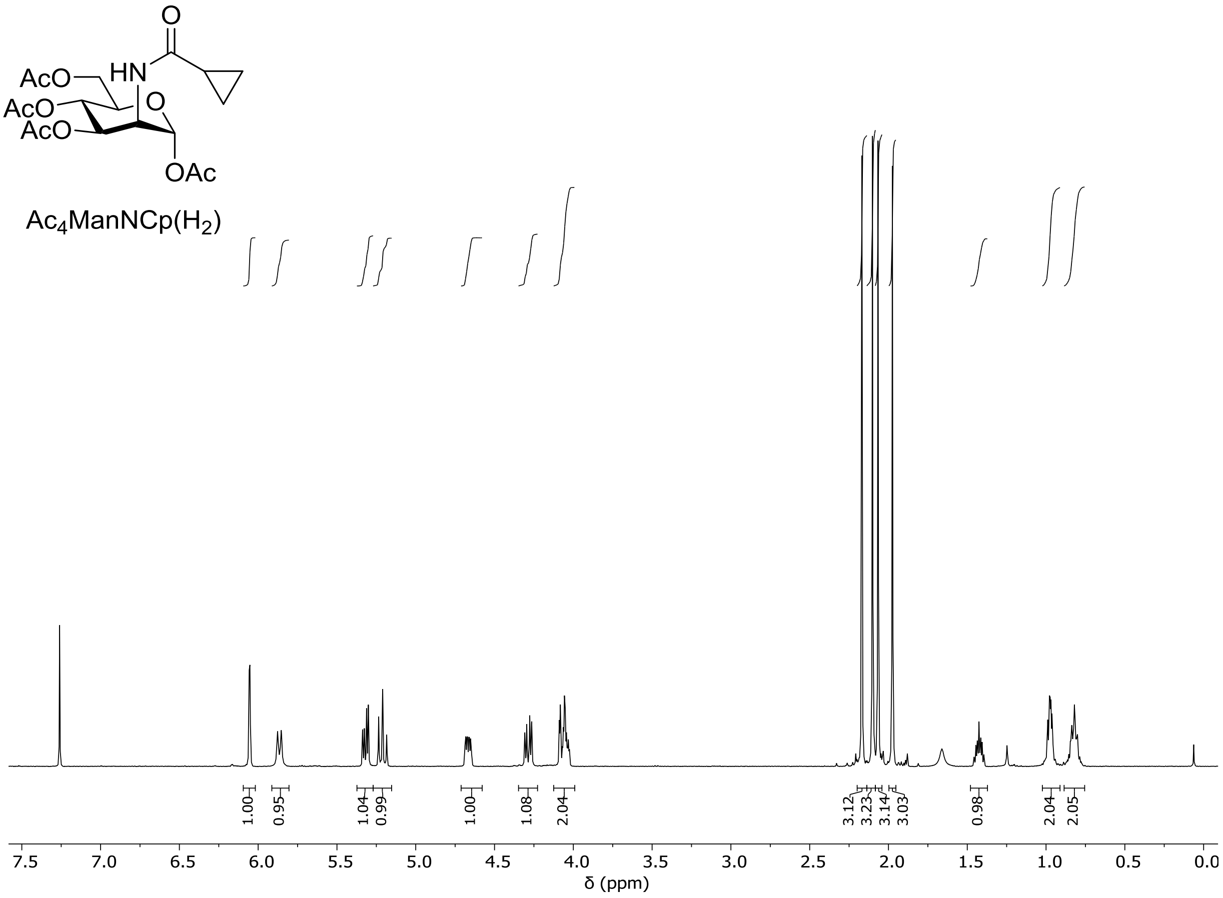

^{1}H NMR spectrum ($CDCl_3$, 400 MHz) of the α-anomer of $Ac_4ManNCp(H_2)$.

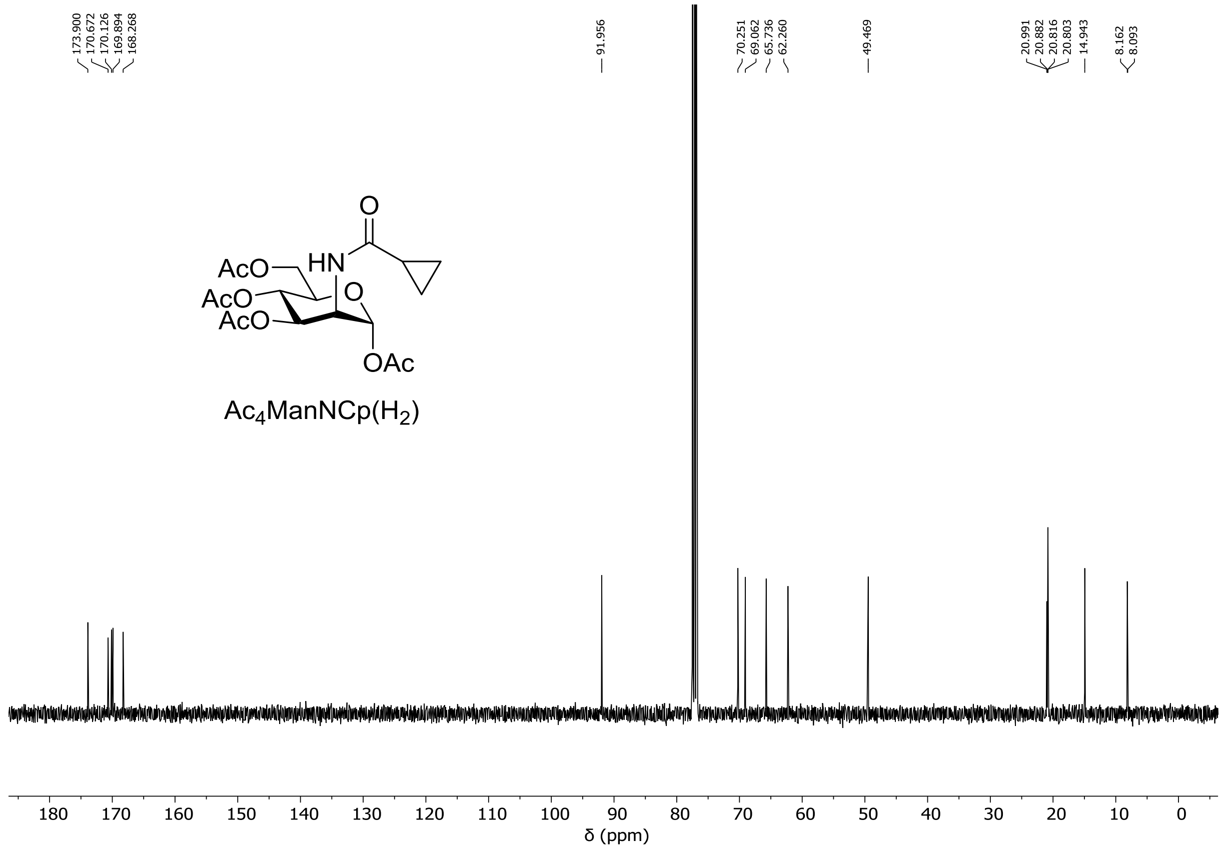

^{13}C NMR spectrum ($CDCl_3$, 101 MHz) of the α-anomer of $Ac_4ManNCp(H_2)$.

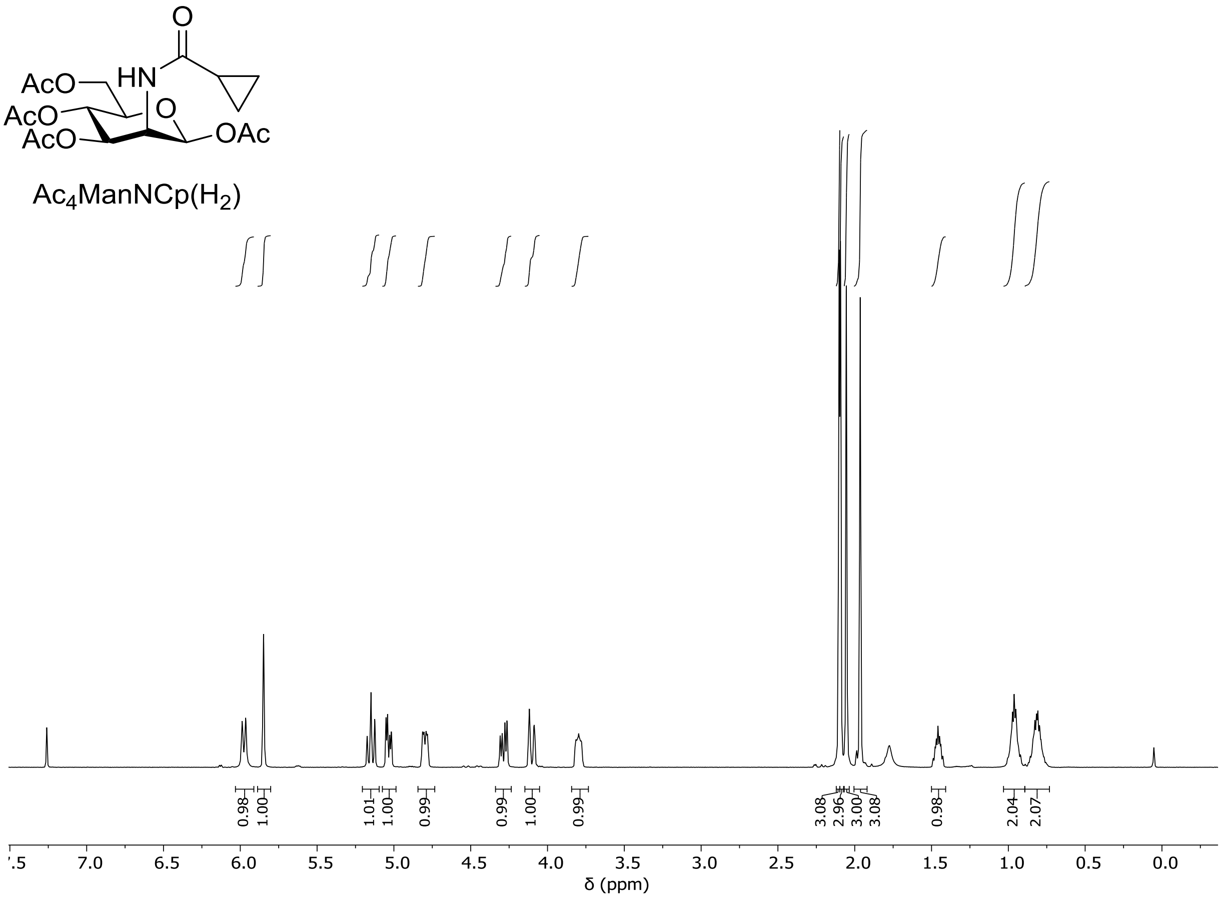

^{1}H NMR spectrum ($CDCl_3$, 400 MHz) of the β-anomer of $Ac_4ManNCp(H_2)$.

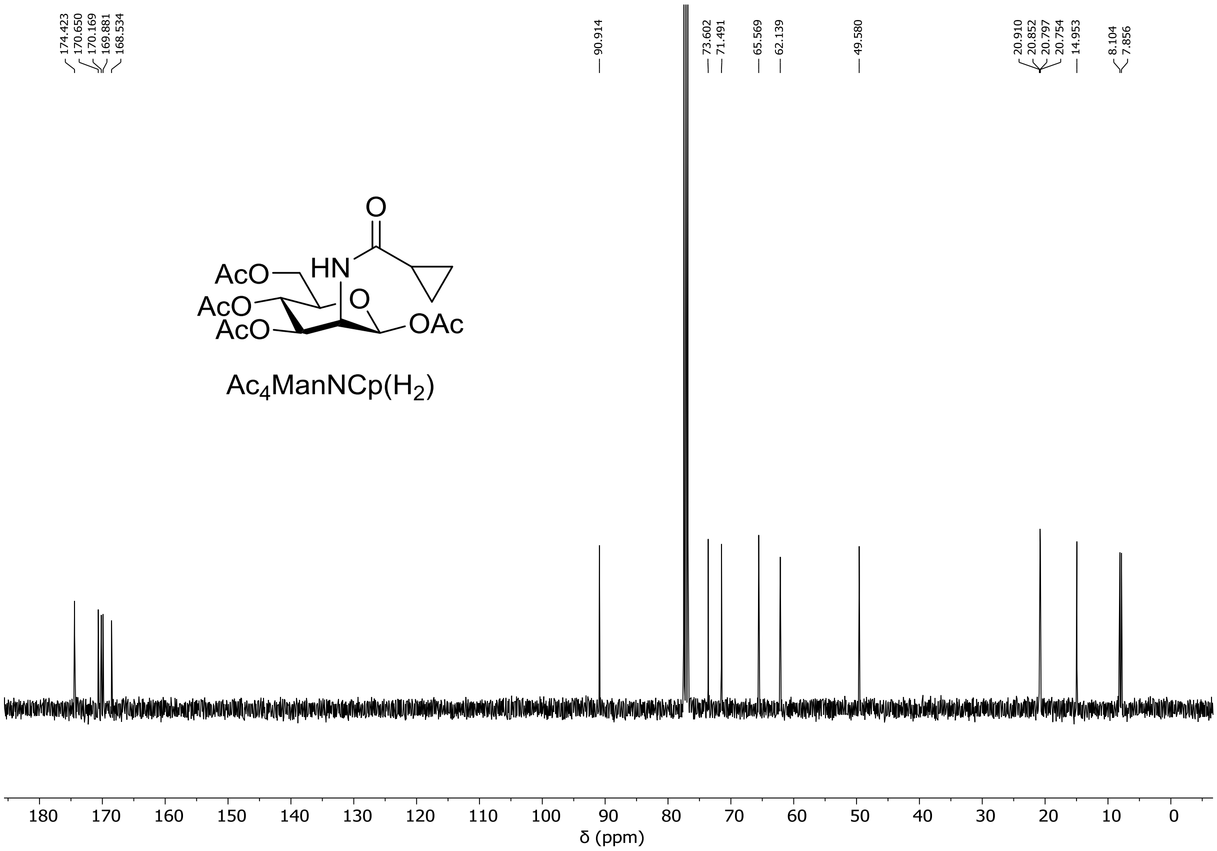

^{13}C NMR spectrum ($CDCl_3$, 101 MHz) of the β-anomer of $Ac_4ManNCp(H_2)$.

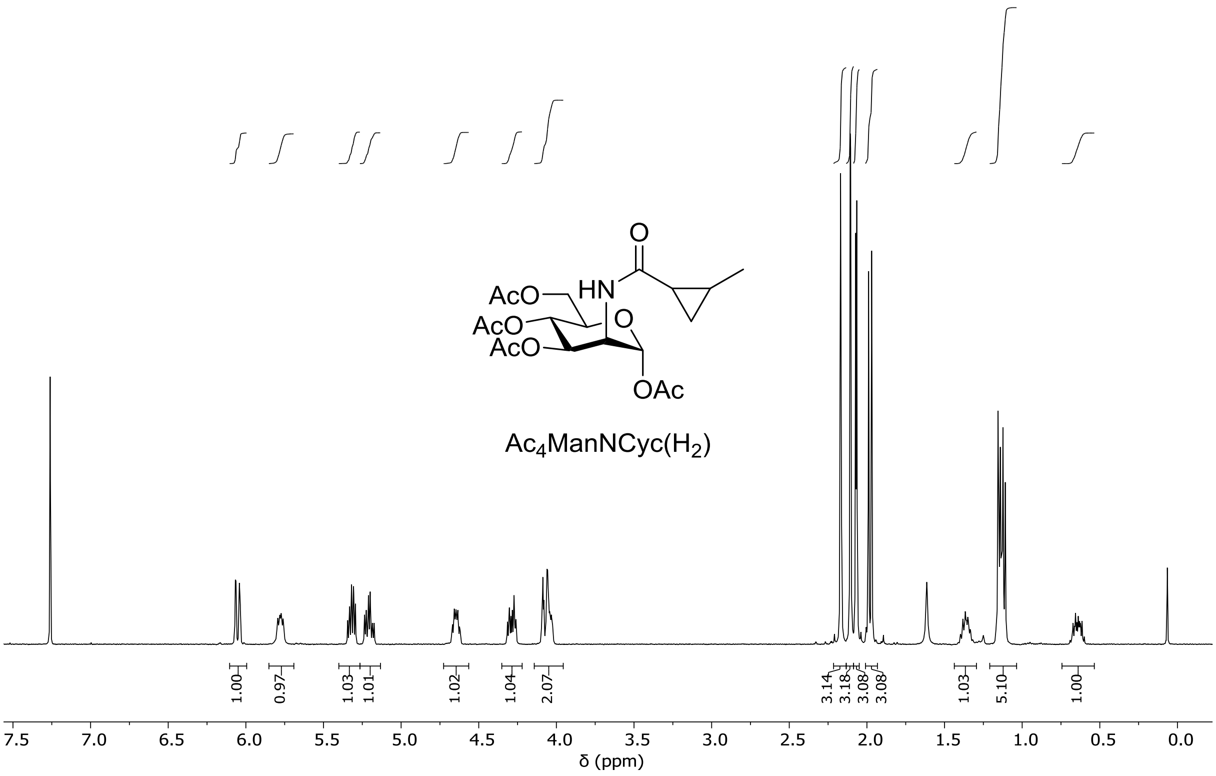

^{1}H NMR spectrum ($CDCl_3$, 400 MHz) of the α-anomer of $Ac_4ManNCyc(H_2)$.

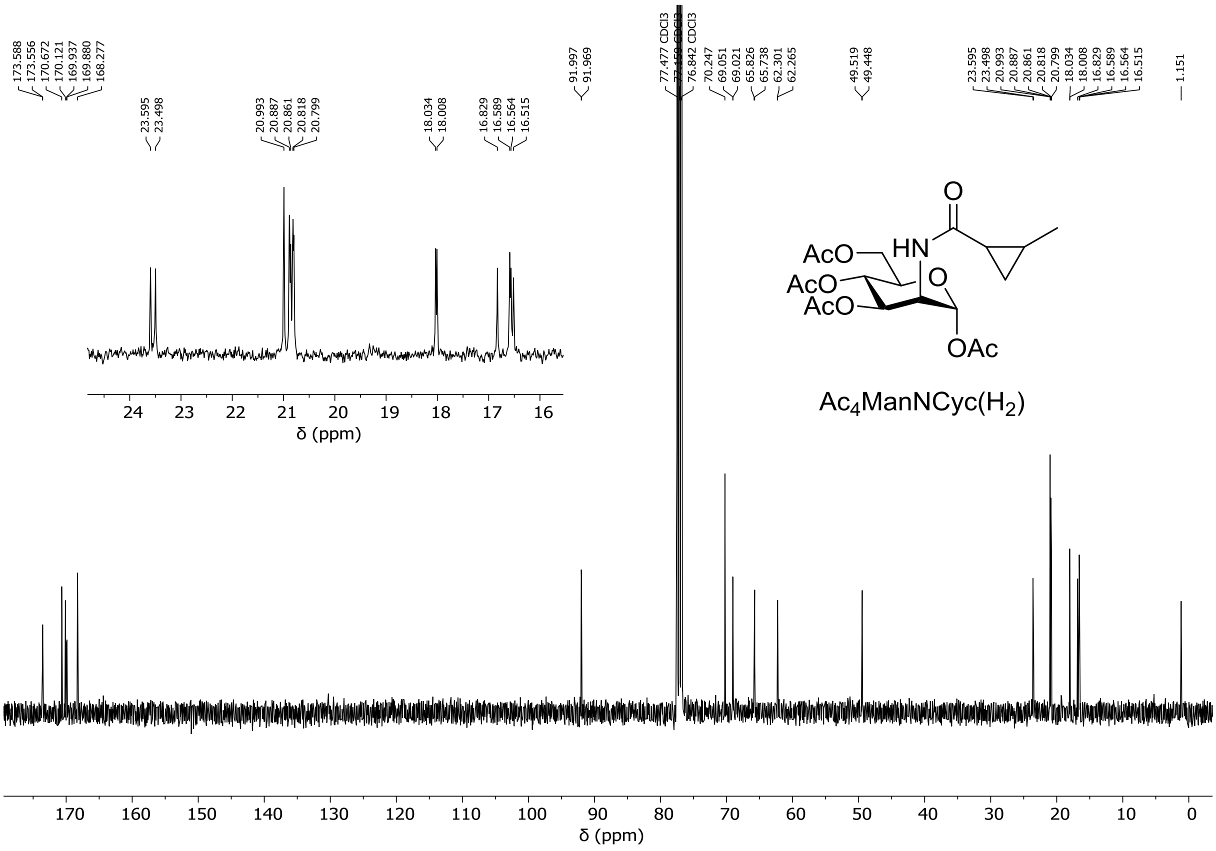

^{13}C NMR spectrum ($CDCl_3$, 101 MHz) of the α-anomer of $Ac_4ManNCyc(H_2)$.

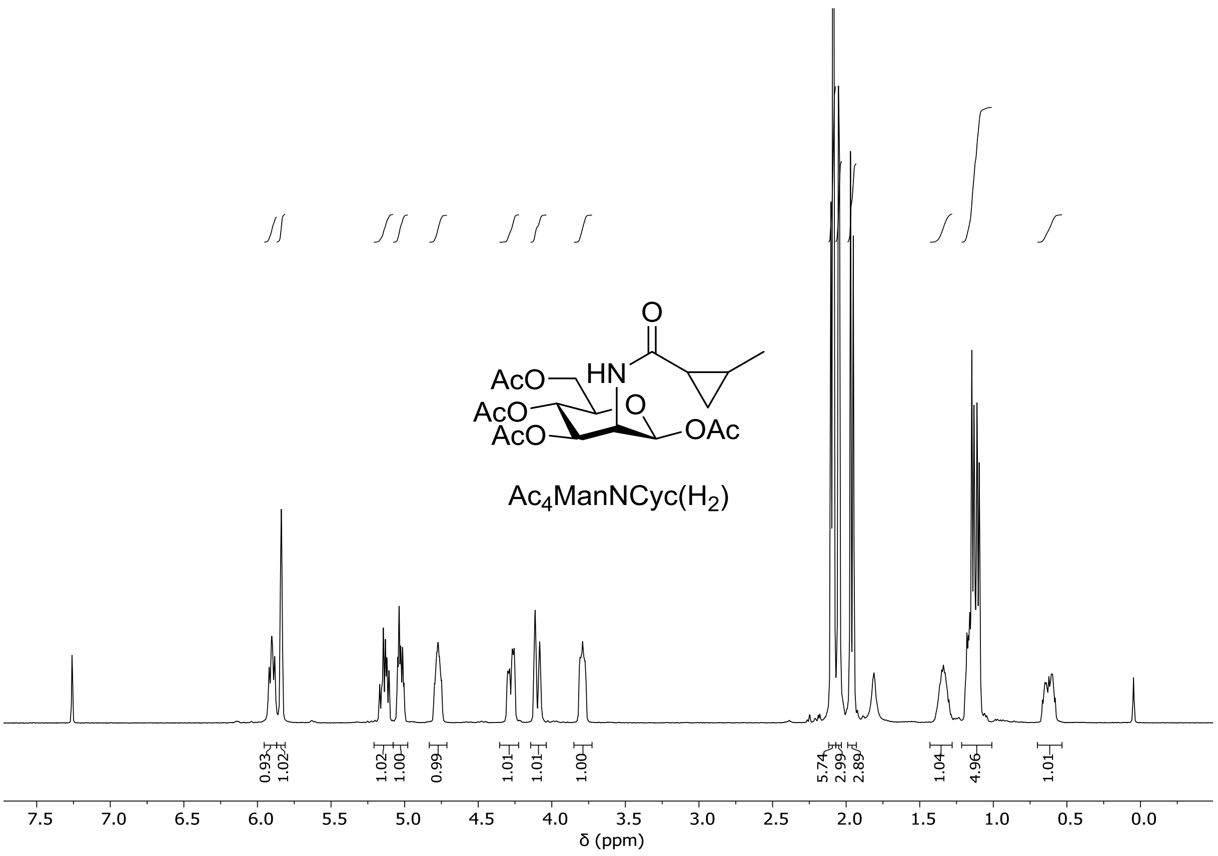

^{1}H NMR spectrum ($CDCl_3$, 400 MHz) of the β-anomer of $Ac_4ManNCyc(H_2)$.

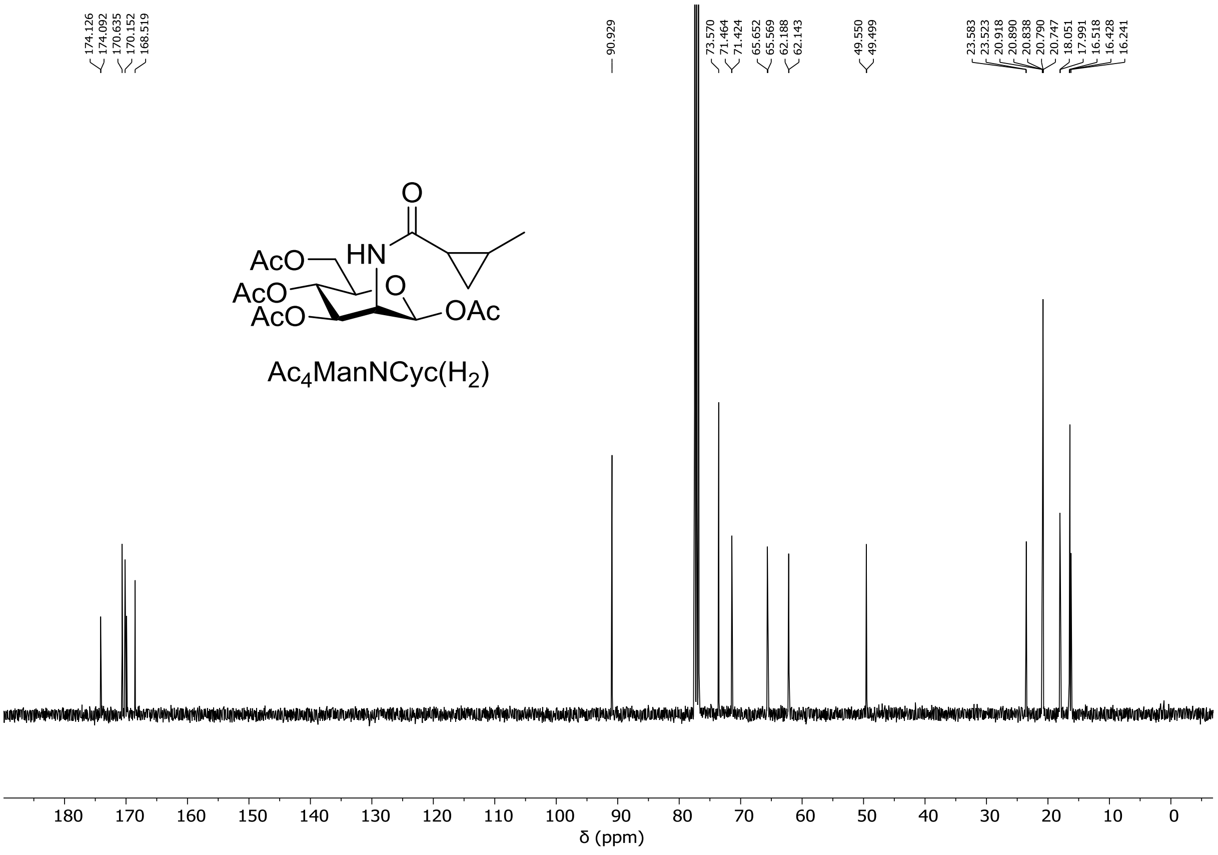

^{13}C NMR spectrum ($CDCl_3$, 101 MHz) of the β-anomer of $Ac_4ManNCyc(H_2)$.

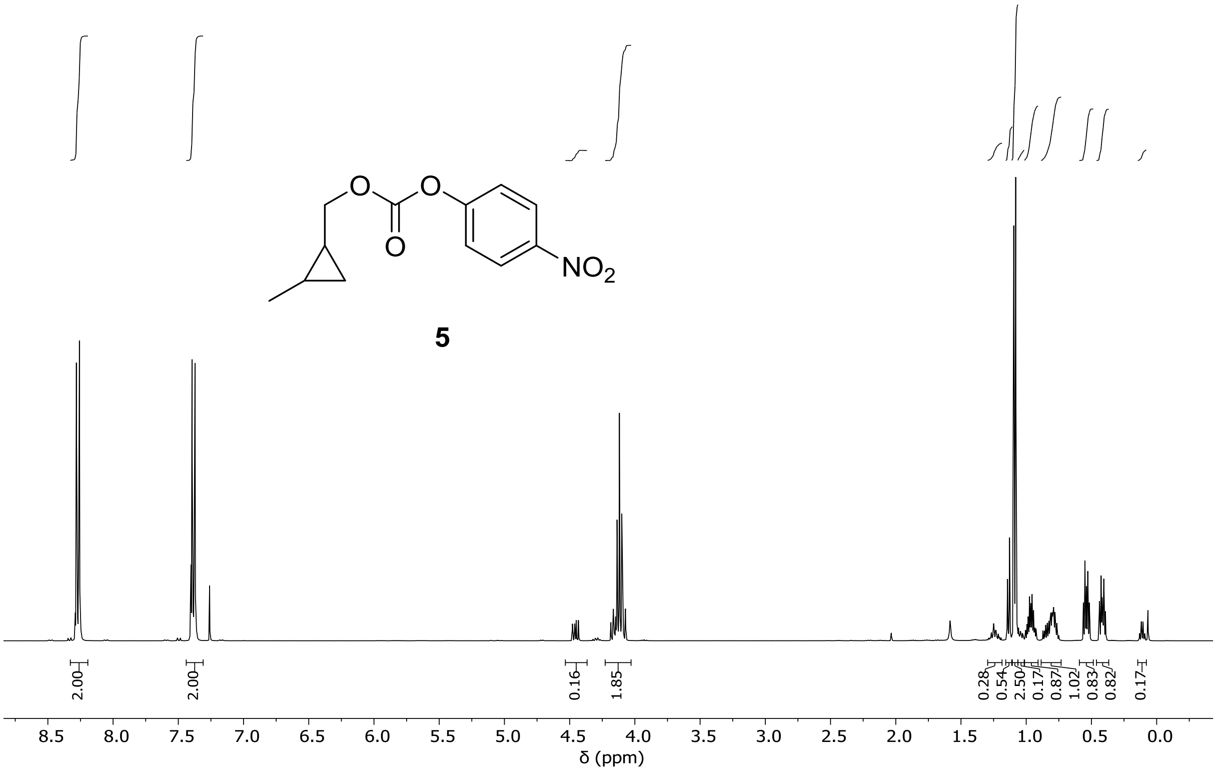

^{1}H NMR spectrum ($CDCl_3$, 400 MHz) of **5**.

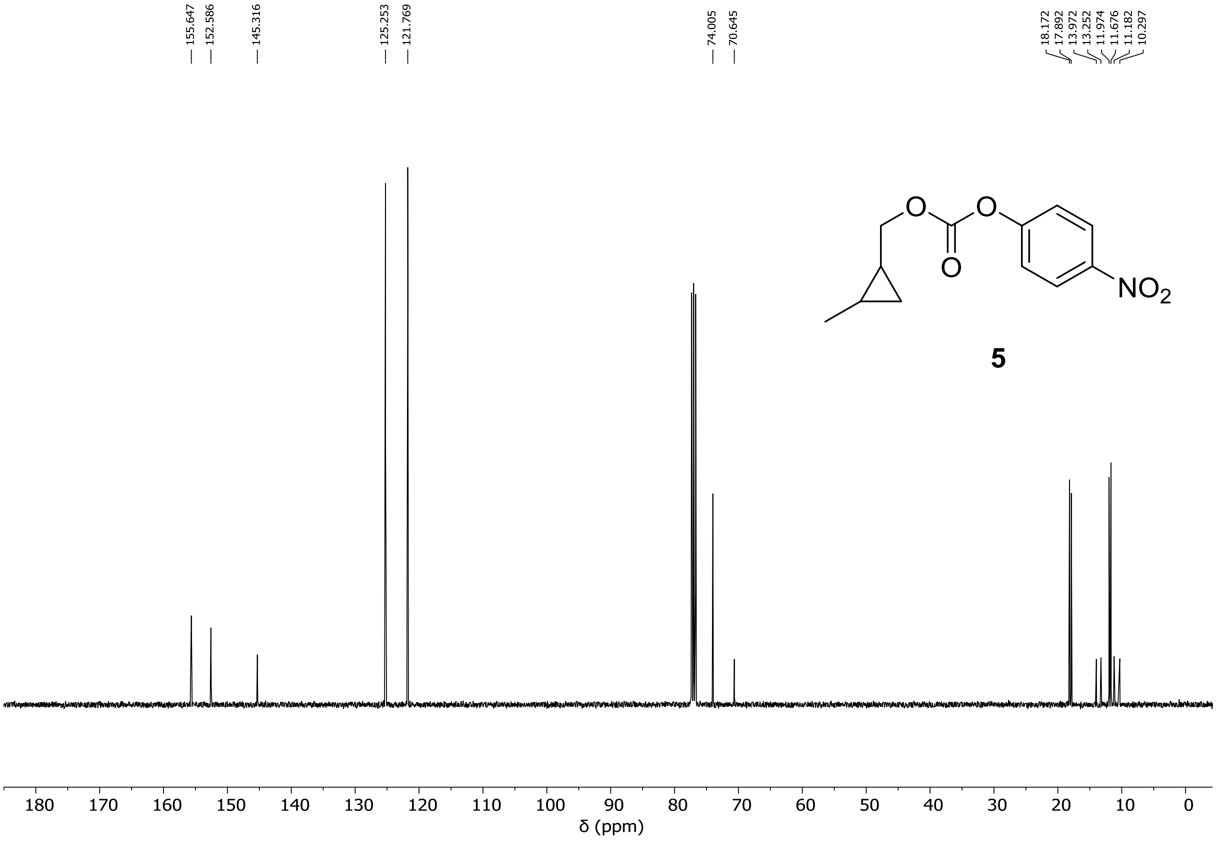

^{13}C NMR spectrum ($CDCl_3$, 101 MHz) of **5**.

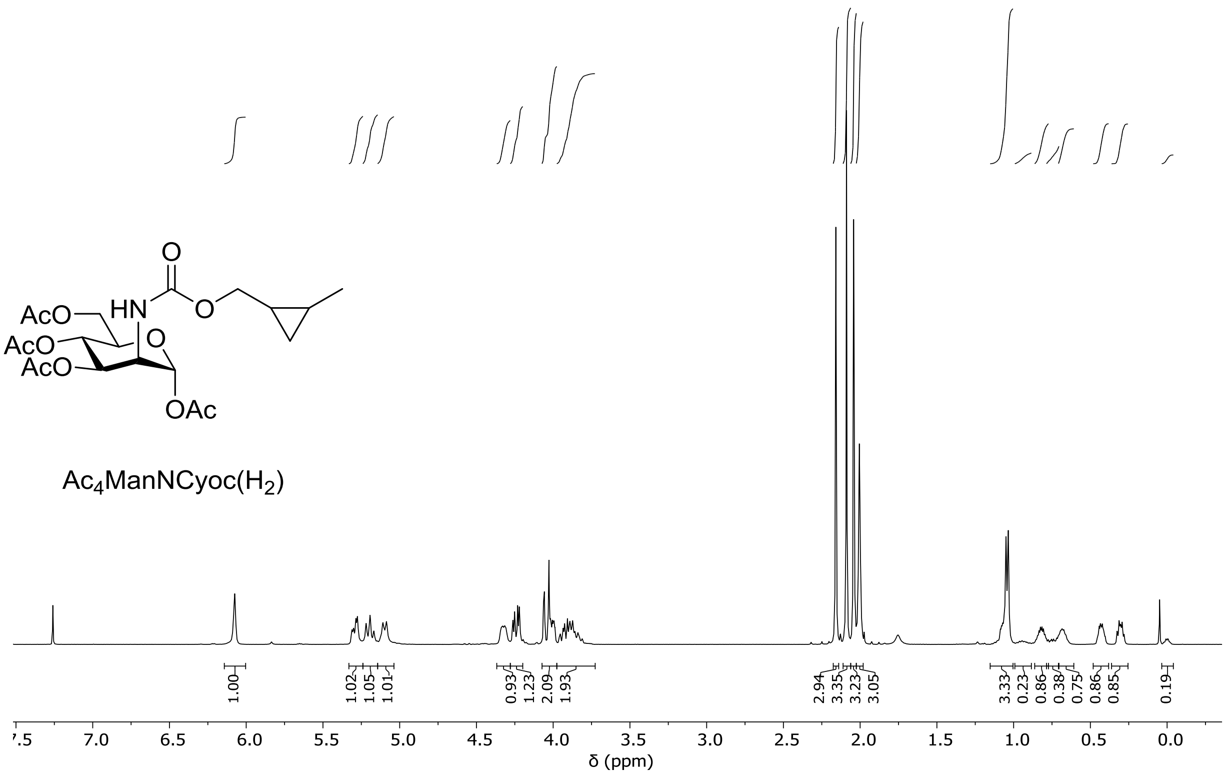

^{1}H NMR spectrum ($CDCl_3$, 400 MHz) of the α-anomer of $Ac_4ManNCyoc(H_2)$.

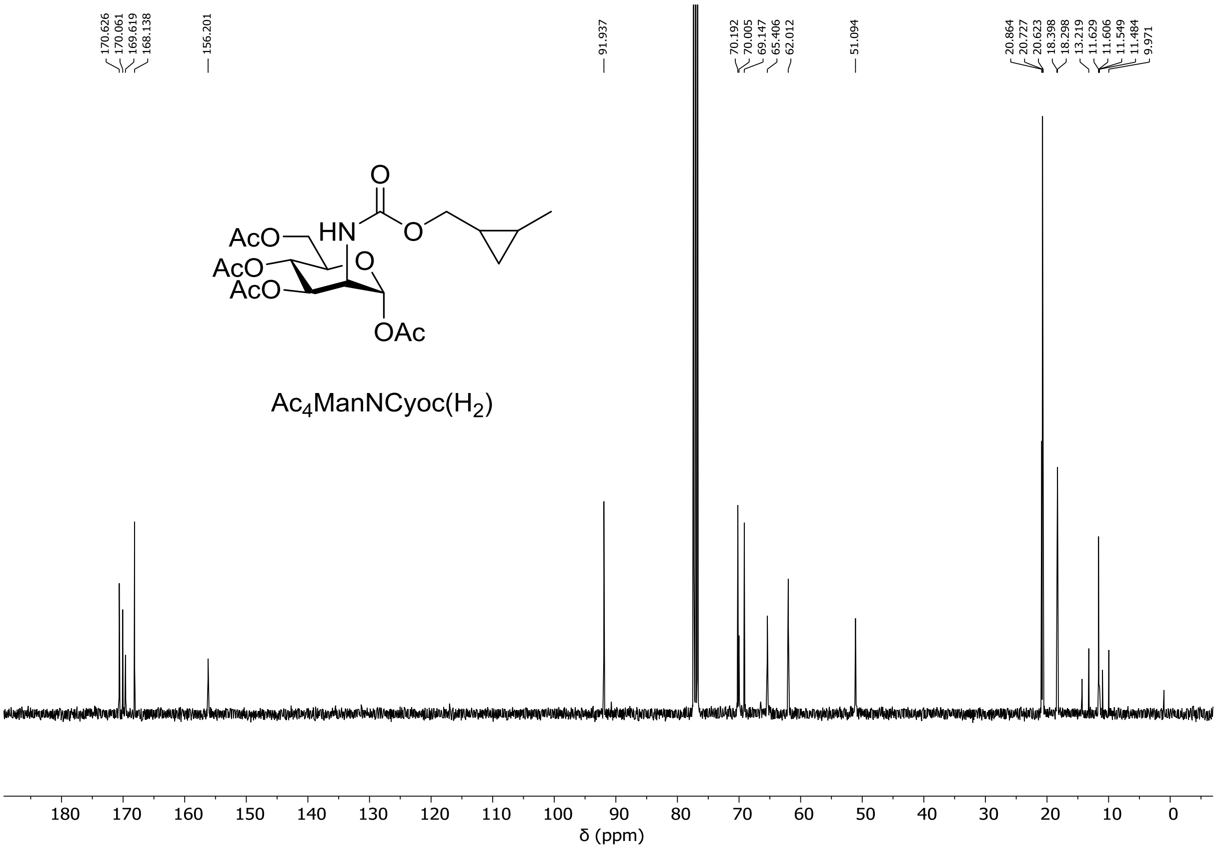

^{13}C NMR spectrum ($CDCl_3$, 101 MHz) of the α-anomer of $Ac_4ManNCyoc(H_2)$.

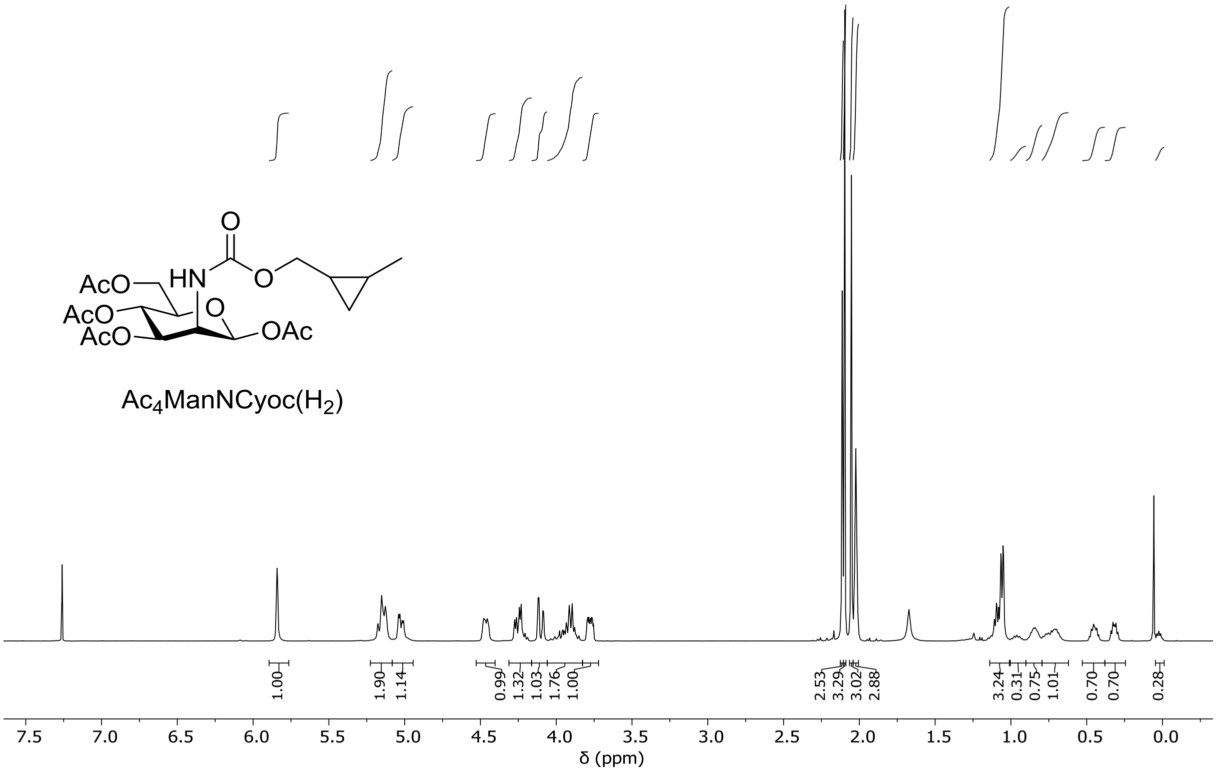

^{1}H NMR spectrum ($CDCl_3$, 400 MHz) of the β-anomer of $Ac_4ManNCyoc(H_2)$.

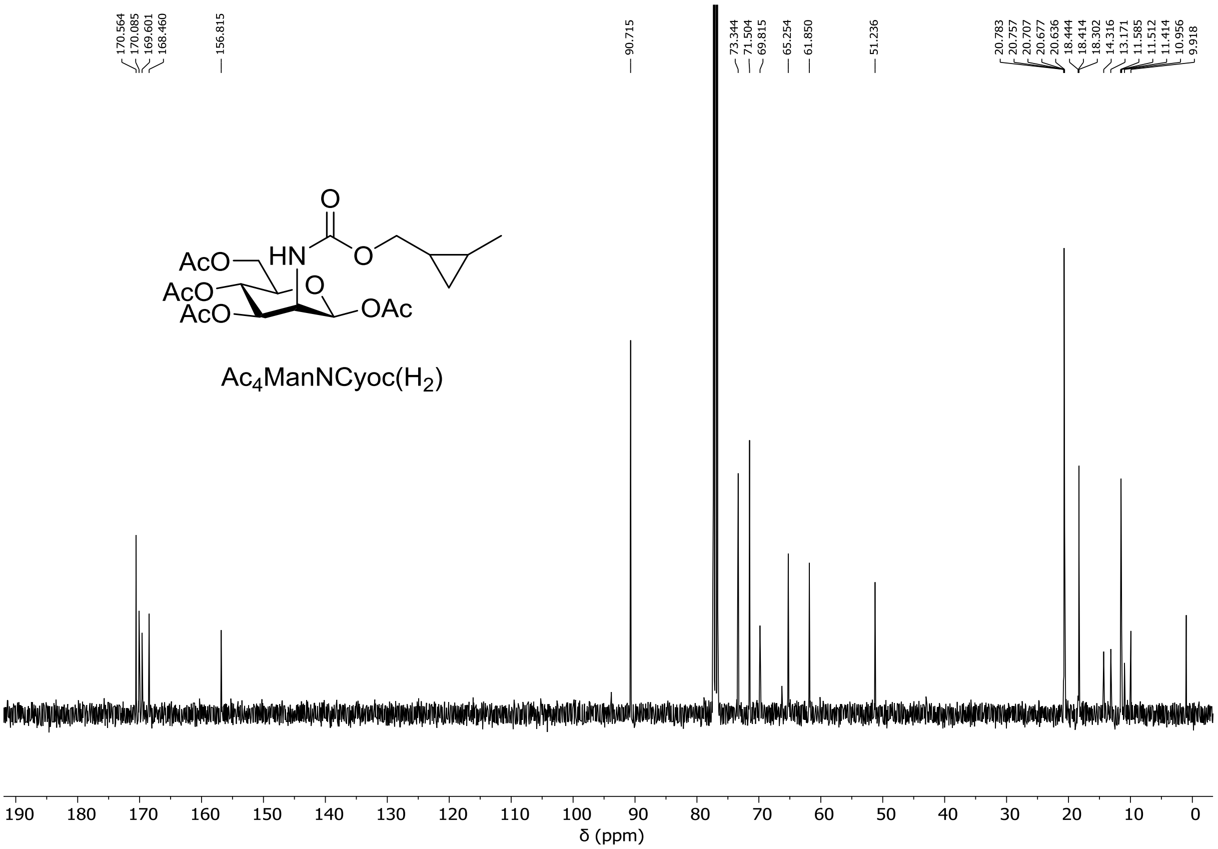

^{13}C NMR spectrum ($CDCl_3$, 101 MHz) of the β-anomer of $Ac_4ManNCyoc(H_2)$.

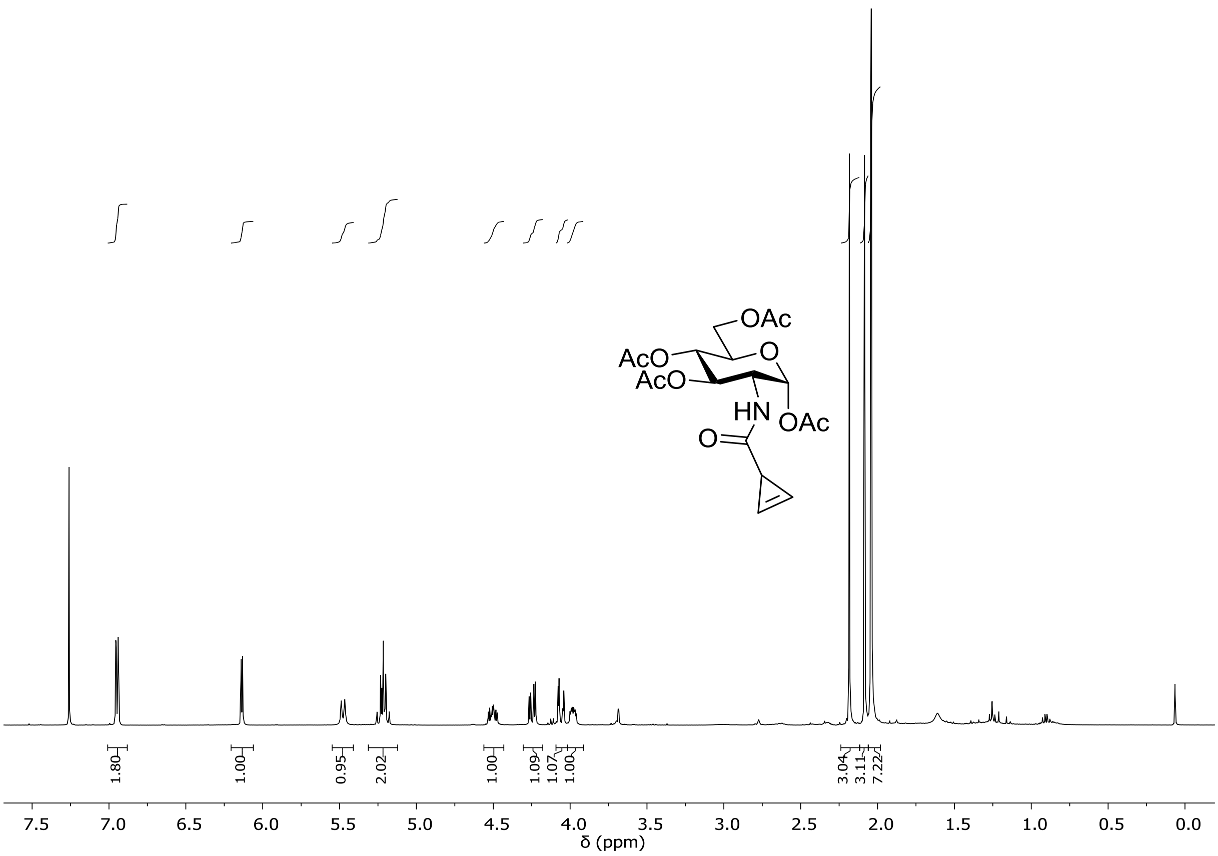

^{1}H NMR spectrum ($CDCl_3$, 400 MHz) of the α-anomer of $Ac_4GlcNCp$.

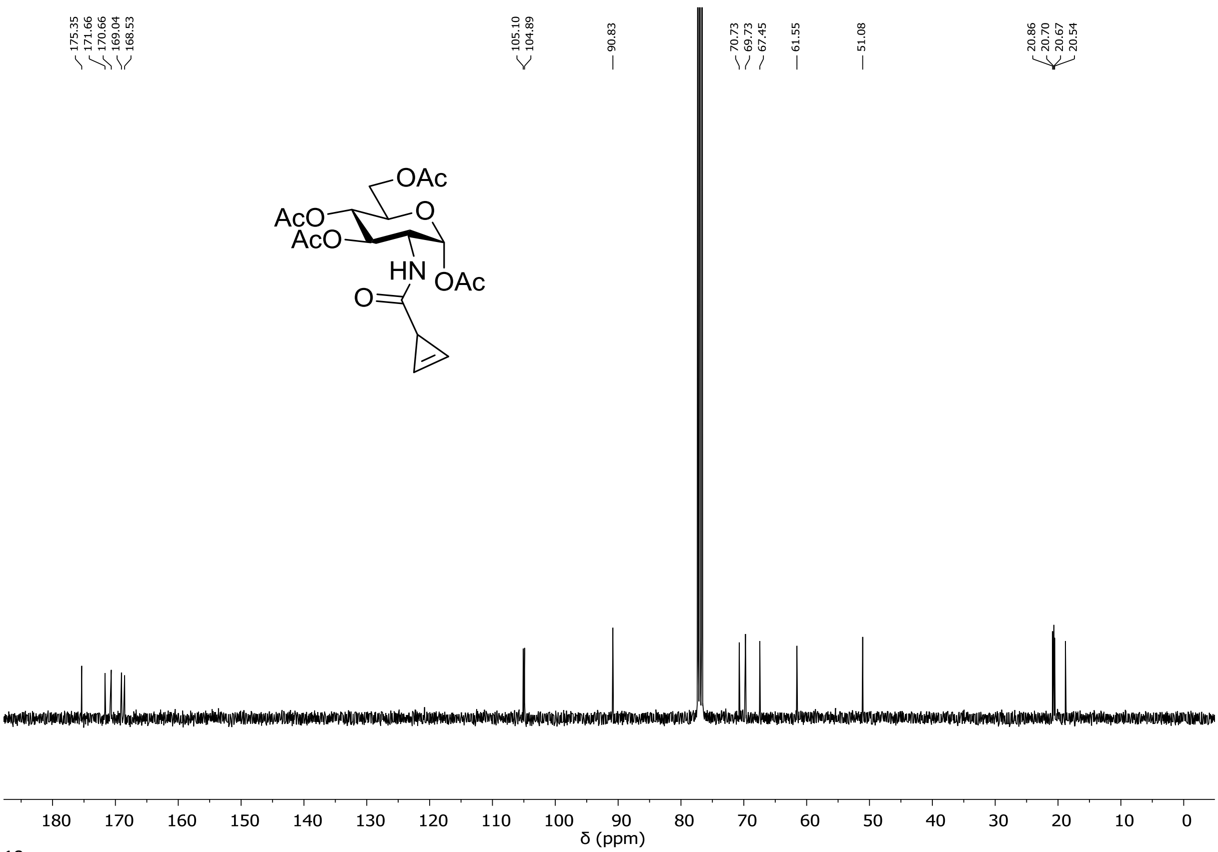

^{13}C NMR spectrum ($CDCl_3$, 101 MHz) of the α-anomer of $Ac_4GlcNCp$.

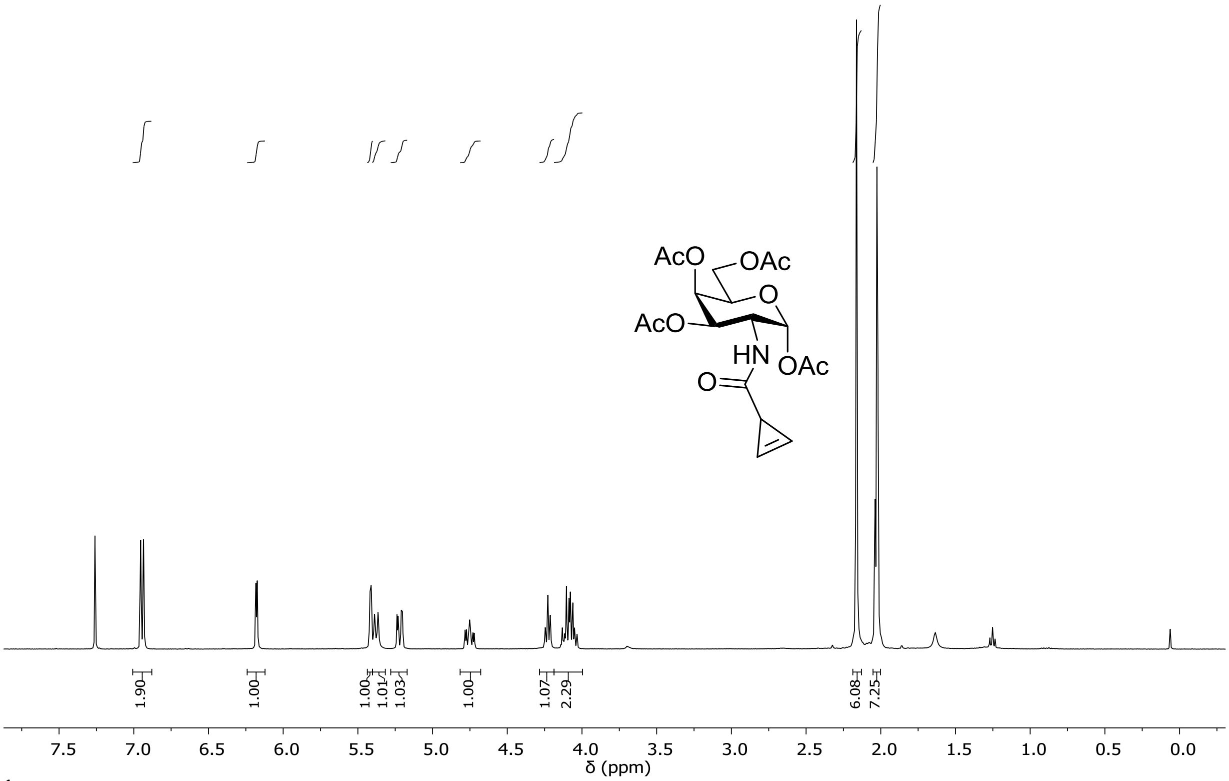

^{1}H NMR spectrum ($CDCl_3$, 400 MHz) of the α-anomer of $Ac_4GalNCp$.

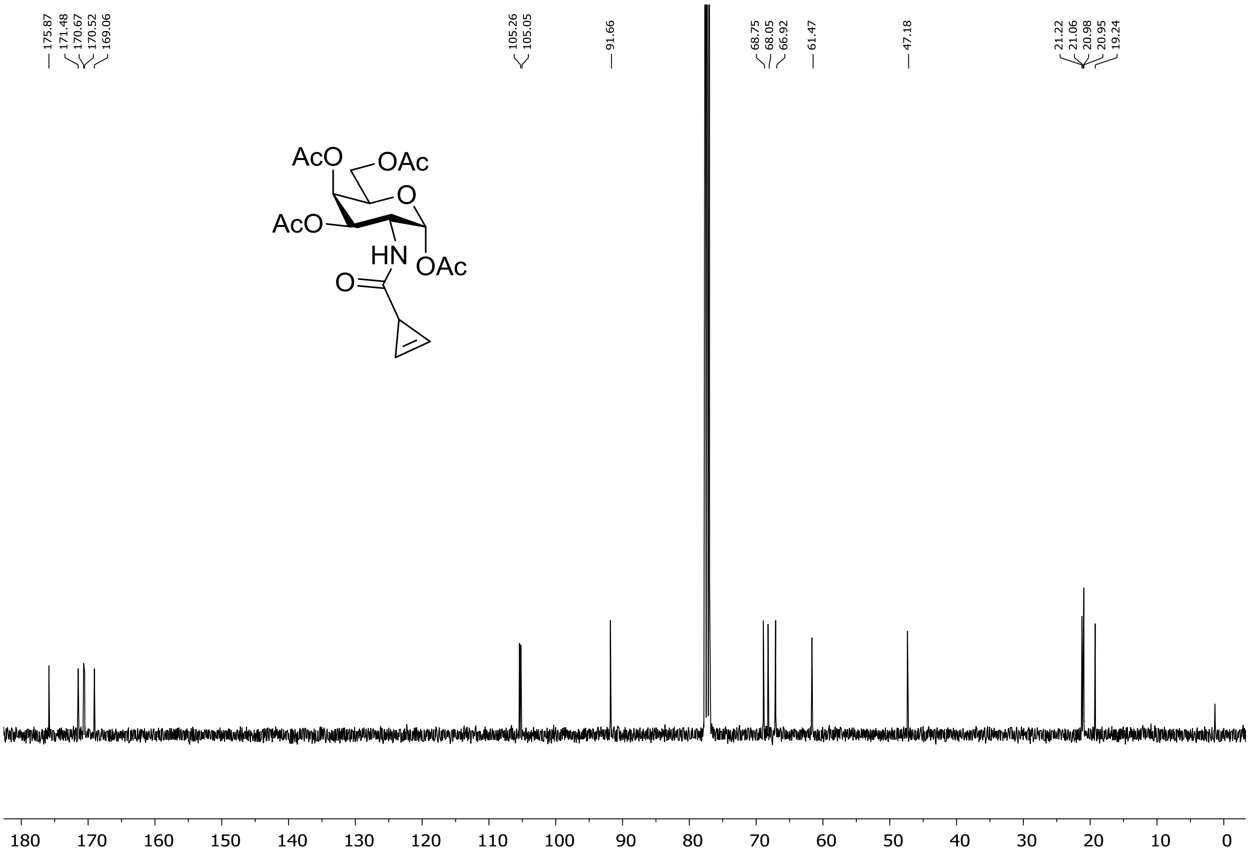

^{13}C NMR spectrum ($CDCl_3$, 101 MHz) of the α-anomer of $Ac_4GalNCp$.

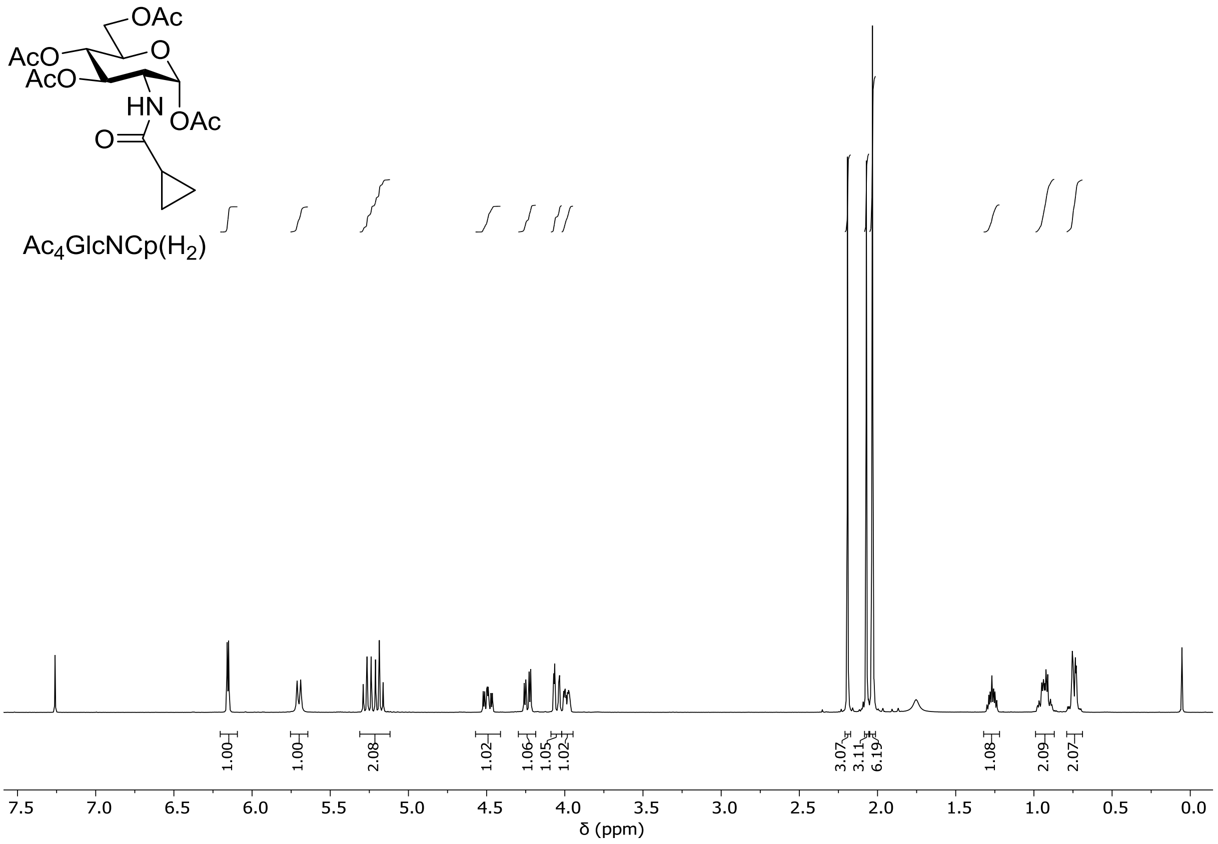

^{1}H NMR spectrum ($CDCl_3$, 400 MHz) of the α-anomer of $Ac_4GlcNCp(H_2)$.

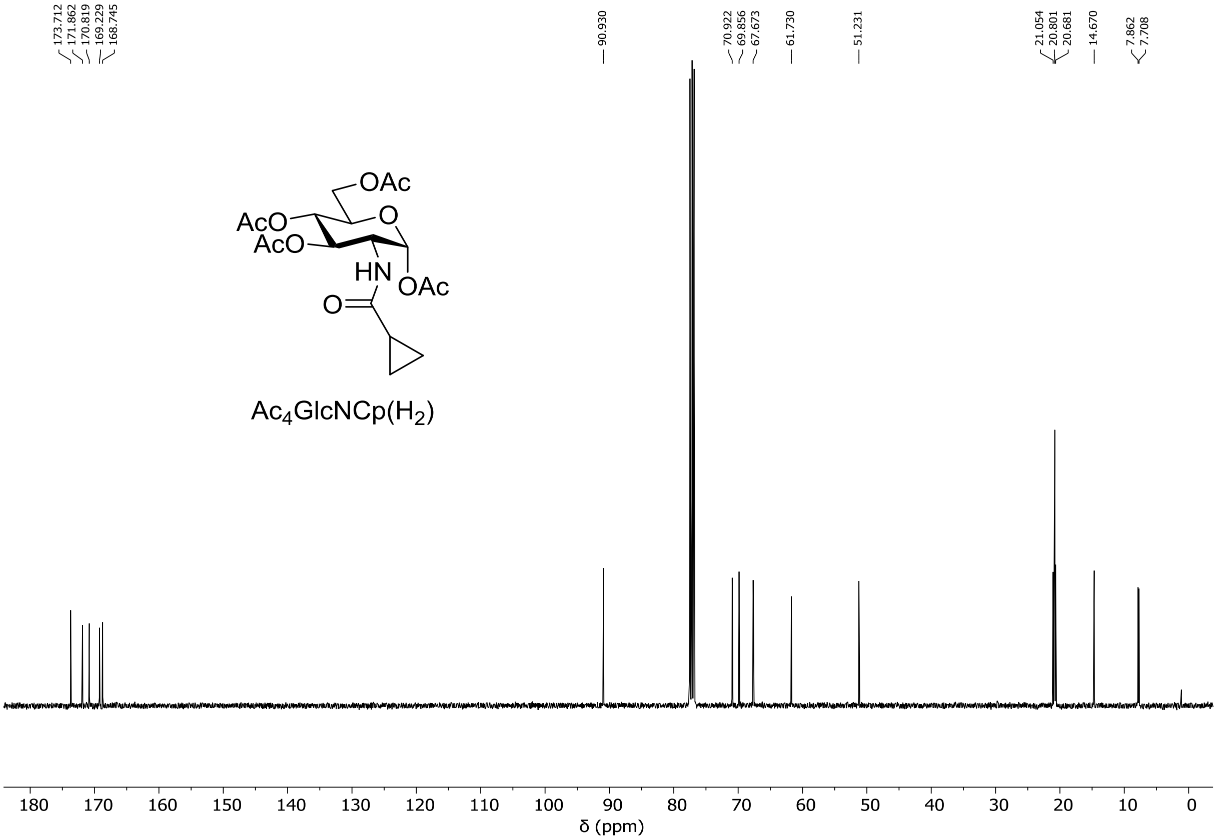

^{13}C NMR spectrum ($CDCl_3$, 101 MHz) of the α-anomer of $Ac_4GlcNCp(H_2)$.

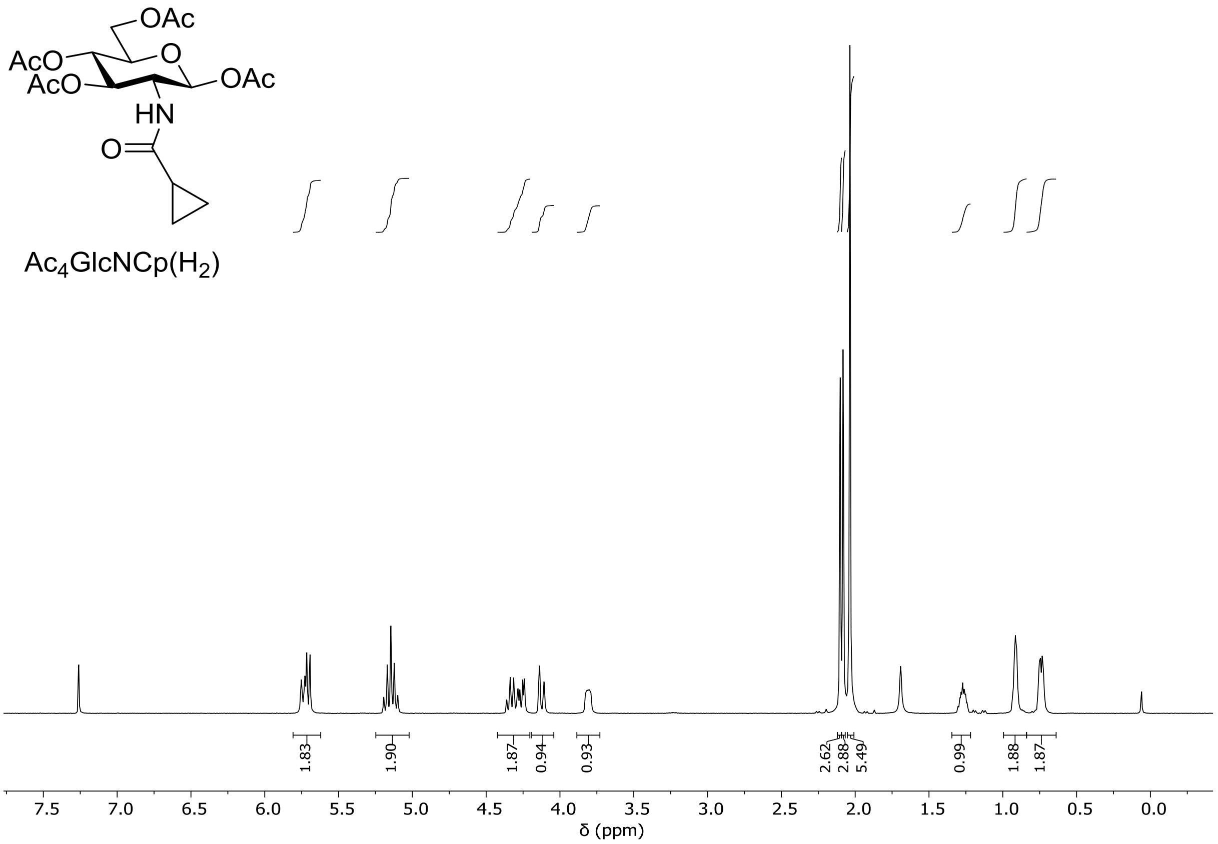

^{1}H NMR spectrum ($CDCl_3$, 400 MHz) of the β-anomer of $Ac_4GlcNCp(H_2)$.

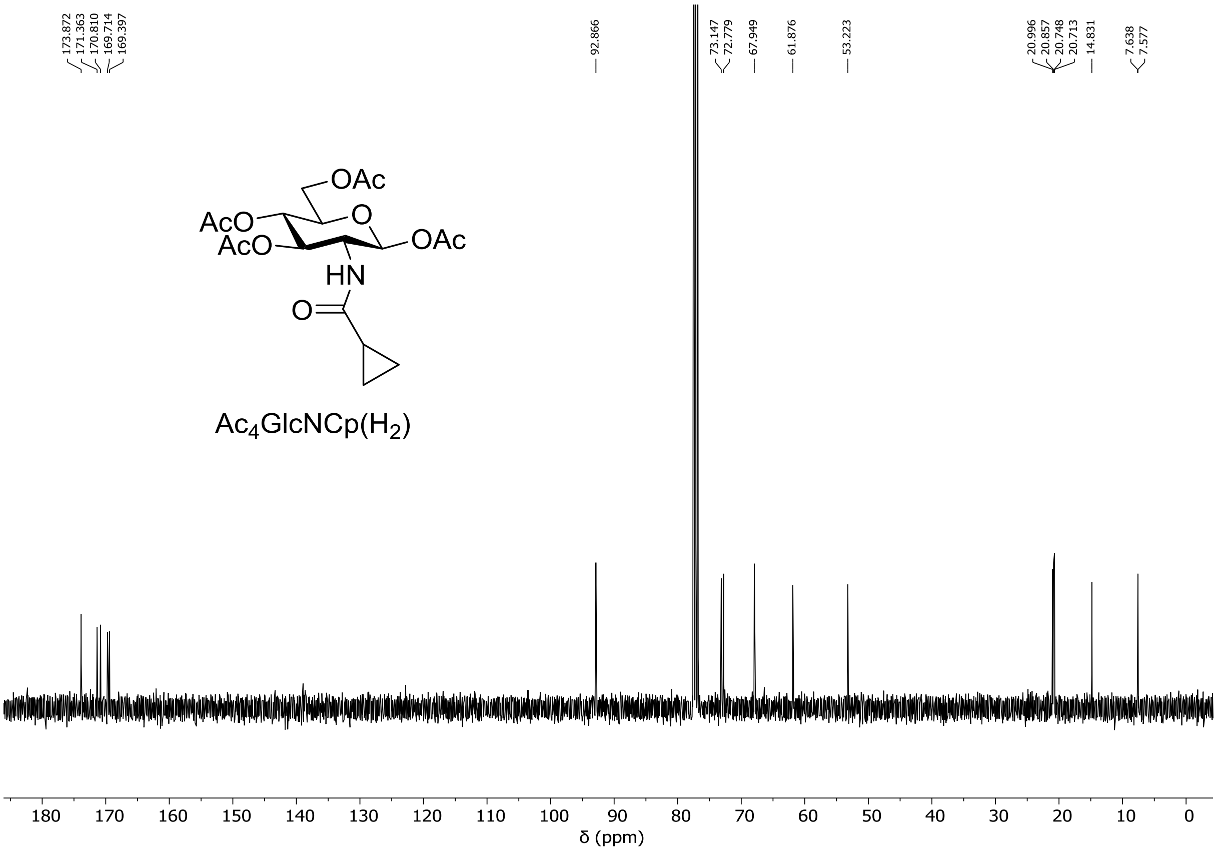

^{13}C NMR spectrum ($CDCl_3$, 101 MHz) of the β-anomer of $Ac_4GlcNCp(H_2)$.

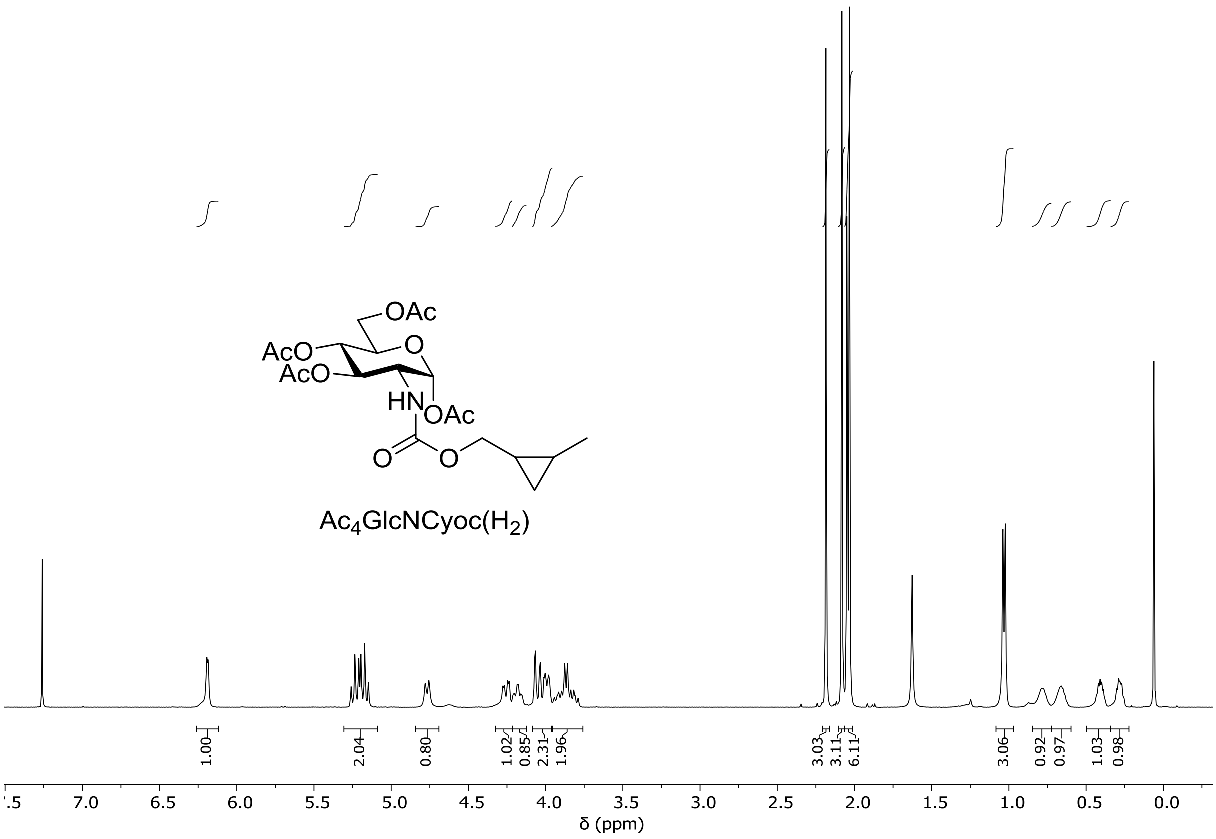

^{1}H NMR spectrum ($CDCl_3$, 400 MHz) of the α-anomer of $Ac_4GlcNCyoc(H_2)$.

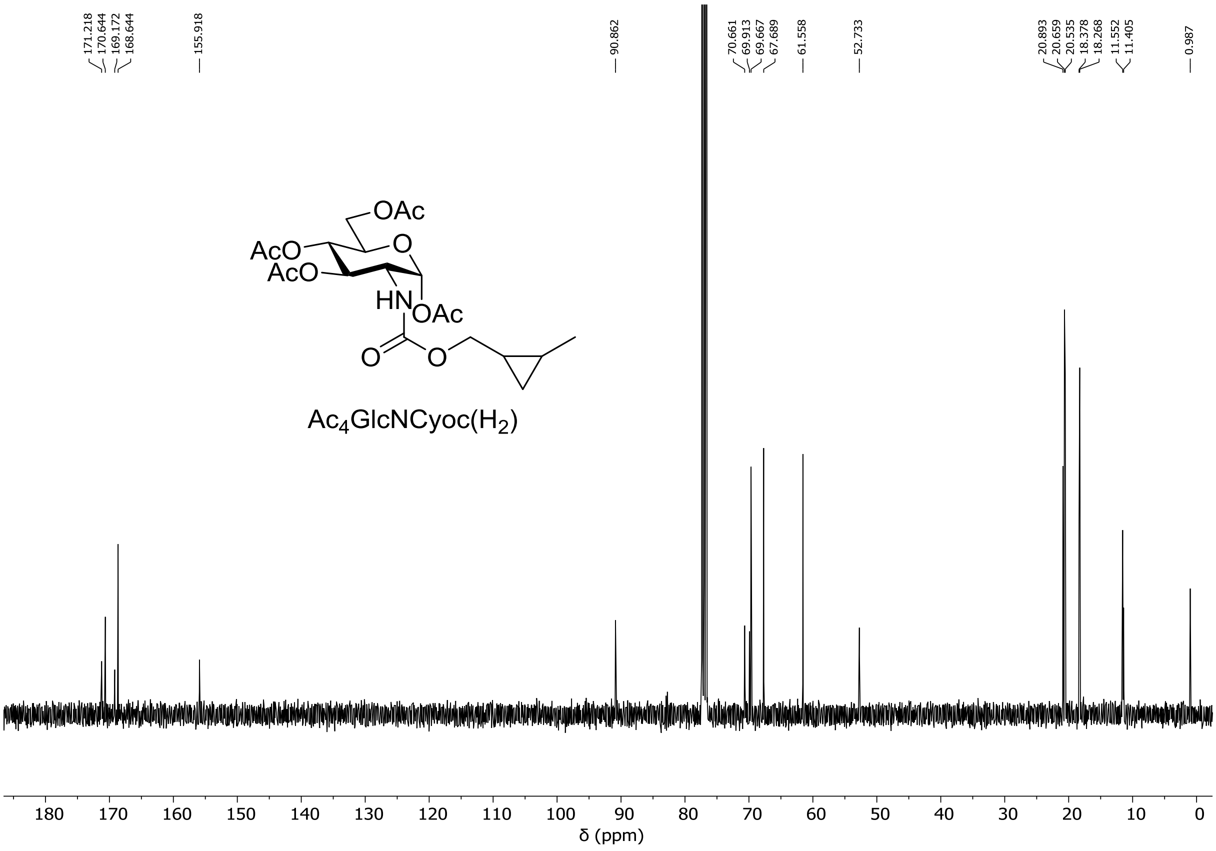

^{13}C NMR spectrum ($CDCl_3$, 101 MHz) of the α-anomer of $Ac_4GlcNCyoc(H_2)$.

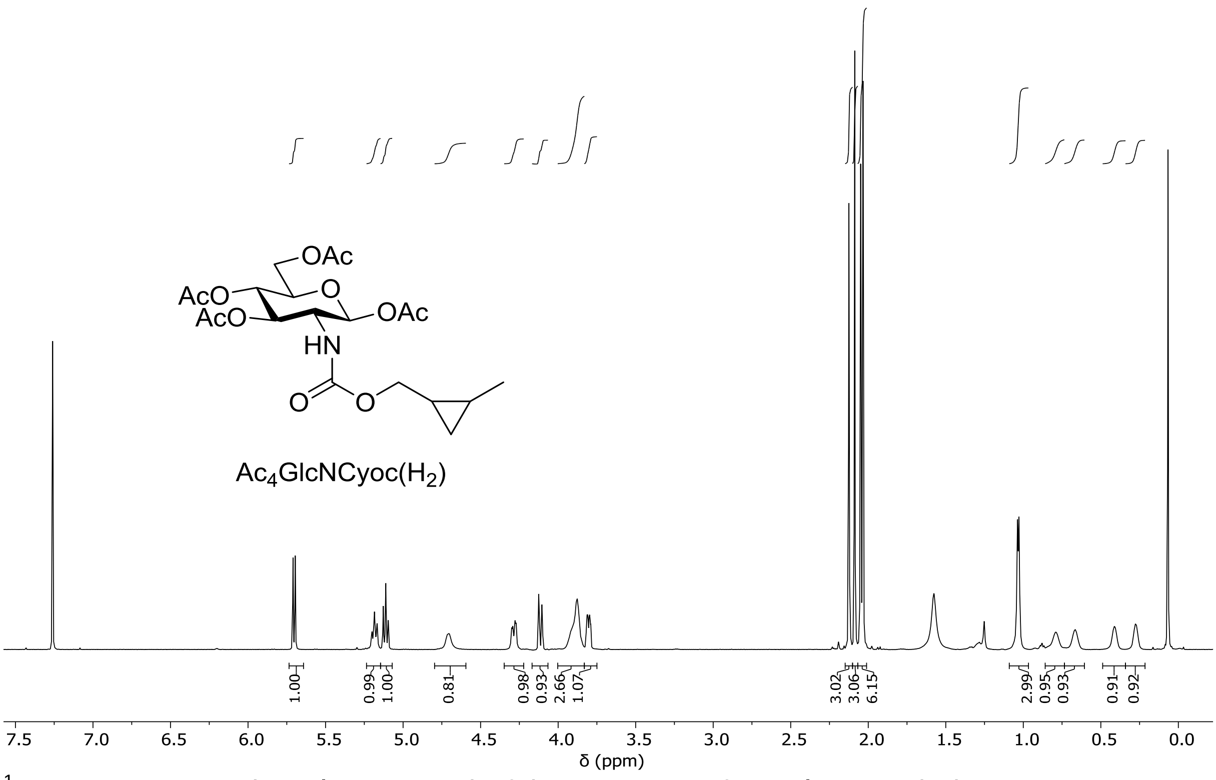

^{1}H NMR spectrum ($CDCl_3$, 600 MHz) of the β-anomer of $Ac_4GlcNCyoc(H_2)$.

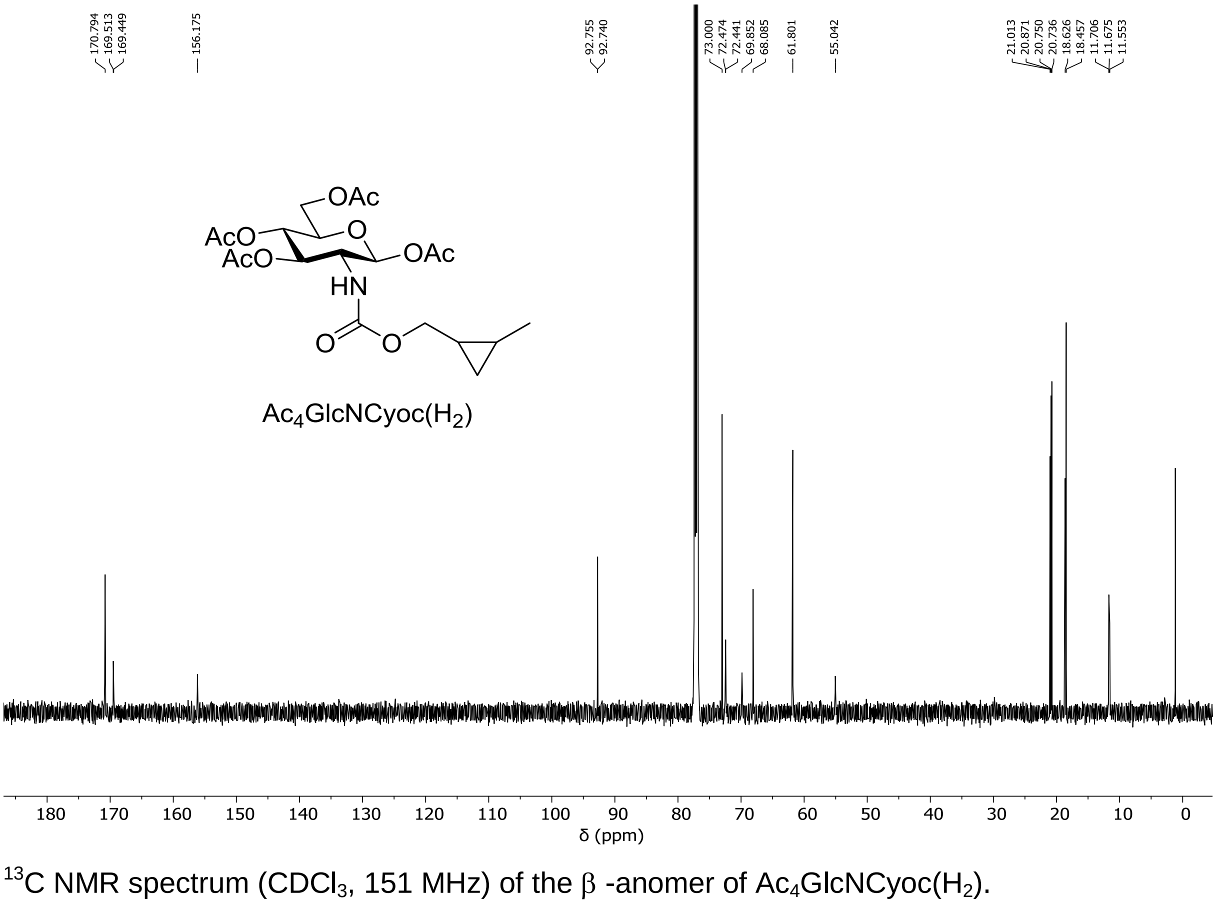

^{13}C NMR spectrum ($CDCl_3$, 151 MHz) of the β -anomer of $Ac_4GlcNCyoc(H_2)$.
